# Scalarane-Type Sesterterpenoids from the Marine Sponge *Lendenfeldia* sp. Alleviate Inflammation in Human Neutrophils

**DOI:** 10.3390/md19100561

**Published:** 2021-09-30

**Authors:** Bo-Rong Peng, Kuei-Hung Lai, Gene-Hsiang Lee, Steve Sheng-Fa Yu, Chang-Yih Duh, Jui-Hsin Su, Li-Guo Zheng, Tsong-Long Hwang, Ping-Jyun Sung

**Affiliations:** 1 Doctoral Degree Program in Marine Biotechnology, National Sun Yat-sen University, Kaohsiung 804201, Taiwan; pengpojung@gmail.com; 2 Department of Planning and Research, National Museum of Marine Biology & Aquarium, Pingtung 944401, Taiwan; x2219@nmmba.gov.tw (J.-H.S.); t0919928409@gmail.com (L.-G.Z.); 3 PhD Program in Clinical Drug, Development of Herbal Medicine, College of Pharmacy, Taipei Medical University, Taipei 110301, Taiwan; kueihunglai@tmu.edu.tw; 4 Graduate Institute of Pharmacognosy, College of Pharmacy, Taipei Medical University, Taipei 110301, Taiwan; 5 Traditional Herbal Medicine Research Center, Taipei Medical University Hospital, Taipei 110301, Taiwan; 6 Instrumentation Center, National Taiwan University, Taipei 106319, Taiwan; ghlee@ntu.edu.tw; 7 Institute of Chemistry, Academia Sinica, Taipei 115201, Taiwan; sfyu@chem.sinica.edu.tw; 8 Department of Marine Biotechnology and Resources, National Sun Yat-sen University, Kaohsiung 804201, Taiwan; yihduh@mail.nsysu.edu.tw; 9 Research Center for Chinese Herbal Medicine, Graduate Institute of Health Industry Technology, College of Human Ecology, Chang Gung University of Science and Technology, Taoyuan 333324, Taiwan; 10 Department of Anaesthesiology, Chang Gung Memorial Hospital, Taoyuan 333423, Taiwan; 11 Graduate Institute of Natural Products, College of Medicine, Chang Gung University, Taoyuan 333323, Taiwan; 12 Department of Chemical Engineering, Ming Chi University of Technology, New Taipei 243303, Taiwan; 13 Chinese Medicine Research and Development Center, China Medical University Hospital, Taichung 404394, Taiwan; 14 Graduate Institute of Natural Products, Kaohsiung Medical University, Kaohsiung 807378, Taiwan; 15 PhD Program in Pharmaceutical Biotechnology, Fu Jen Catholic University, New Taipei 242062, Taiwan

**Keywords:** homoscalarane, *Lendenfeldia*, molecular networking, anti-neutrophilic inflammation

## Abstract

Sponge-derived scalaranes are remarkable sesterterpenoids previously found to exhibit profound inhibitory effects against neutrophilic inflammation. In our current work, we constructed the metabolomic profile of marine sponge *Lendenfeldia* sp. for the first time using a tandem mass spectrometry (MS/MS) molecular networking approach. The results highlighted the rich chemical diversity of these scalaranes, motivating us to conduct further research to discover novel scalaranes targeting neutrophilic inflammation. MS- and NMR-assisted isolation and elucidation led to the discovery of seven new homoscalaranes, lendenfeldaranes K–Q (**1**–**7**), characterized by methylation at C-24, together with five known derivatives, lendenfeldarane B (**8**), 25-nor-24-methyl-12,24-dioxoscalar-16-en-22-oic acid (**9**), 24-methyl-12,24,25-trioxoscalar-16-en-22-oic acid (**10**), felixin B (**11**), and 23-hydroxy-20-methyldeoxoscalarin (**12**). Scalaranes **1**–**4** and **6**–**12** were assayed against superoxide anion generation and elastase release, which represented the neutrophilic inflammatory responses of respiratory burst and degranulation, respectively. The results indicated that **1**–**3** and **6**–**12** exhibited potential anti-inflammatory activities (IC_50_ for superoxide anion scavenging: 0.87~6.57 μM; IC_50_ for elastase release: 1.12~6.97 μM).

## 1. Introduction

*Lendenfeldia* is one of the most common marine sponge genera in tropical and subtropical areas worldwide. It has been studied as a medicinal treasure, and 59 bioactive secondary metabolites in total have been isolated and characterized up to 2020 [1,2,3,4,5,6,7,8,9,10,11,12,13]. In terms of their metabolomic features, the main constituents derived from *Lendenfeldia* sponges are sesterterpenoids, in particular scalaranes classified as 26C-homoscalaranes [1,2,3,8,9,10,11,12,13]. Biological reports of natural products from this sponge revealed pronounced bioactivities, including anti-inflammation [1,9,11], cytotoxicity [2,3,10,12], antimicrobial activity [5,6], α-glucosidase inhibition [7], antifouling activity [8], anti-fungal activity [11], and antiplatelet activity [11]. In addition, research has been performed by our team to illustrate for the first time the anti-inflammatory potential of these homoscalaranes [1]. Such findings encouraged further clarification of metabolomic diversity to discover more anti-inflammatory scalaranes from the marine sponge *Lendenfeldia* sp.

Tandem mass spectrometry (MS/MS) technology is the most commonly-used method for extensive analysis of biological metabolites, because it can extract useful chemical structure information from complex mixtures in addition to molecular weights. In recent years, molecular networking (MN) methods derived from MS/MS technology have been applied in the study of microorganisms, marine organisms, fungi, plants and other products from natural sources [14,15]. MN is a strategy for visualizing MS/MS data, which can present the relevance between chemical structures [16]. Analytical calculation in MN is based on MS^2^ fragmentations, which can symbolize structural characteristics. Fragments with similar MS^2^ fragmentations are connected and clustered together on the chemical molecular network virtual map to highlight the structure and the degree of relevance. In the usual MS/MS technique, the spectra of most molecules cannot be identified due to a lack of references from databases. This novel chemical MN method can provide more meaningful structural correlative information, and represents an excellent tool for comprehensive exploration of metabolic diversity [17].

**Figure 1:**
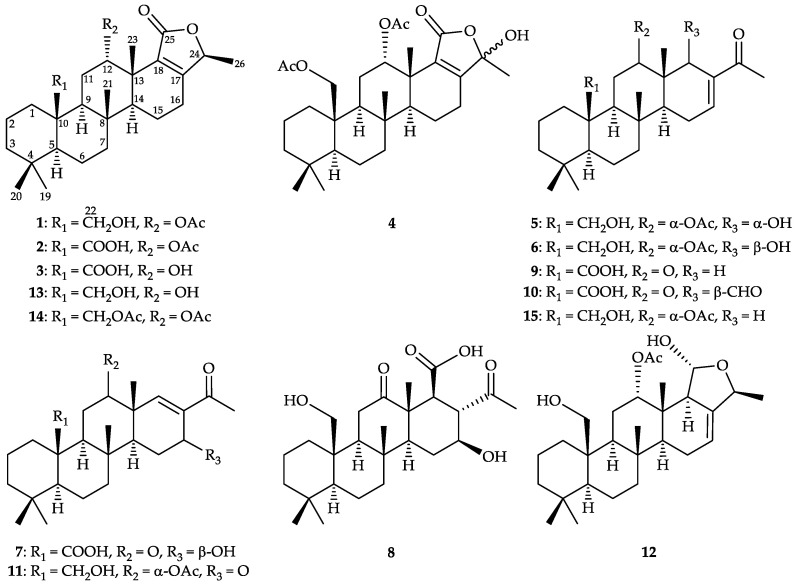
Structures of lendenfeldaranes K–Q (**1**–**7**), lendenfeldarane B (**8**), 25-nor-24-methyl-12,24-dioxoscalar-16-en-22-oic acid (**9**), 24-methyl-12,24,25-trioxoscalar-16-en-22-oic acid (**10**), felixin B (**11**), 23-hydroxy-20-methyldeoxoscalarin (**12**), lendenfeldaranes C (**13**) and D (**14**), and felixin A (**15**).

In the current study, the metabolomic profile of marine sponge *Lendenfeldia* sp. was framed for the first time based on an analysis processed via Global Natural Products Social Molecular Networking (GNPS), a web-based mass spectrometry ecosystem. The visual correlations between compounds and the metabolomic diversity were interpreted. Subsequent fractionation and purification of these scalarane-networked MS^1^ ions resulted in the isolation of seven new 24-homoscalarane-type sesterterpenoids, lendenfeldaranes K–Q (**1**–**7**), and five known analogues, lendenfeldarane B (**8**) [2], 25-nor-24-methyl-12,24-dioxoscalar-16-en-22-oic acid (**9**) [18], 24-methyl-12,24,25-trioxoscalar-16-en-22-oic acid (**10**) [11], felixin B (**11**) [19], and 23-hydroxy-20-methyldeoxoscalarin (**12**) [20] (Figure 1). Anti-neutrophilic assessments of these compounds were performed to examine their potential to inhibit superoxide anion (O_2_^•−^) generation and elastase release.

## 2. Results and Discussion

### 2.1. Exploring the Metabolomic Diversity of Marine Sponge Lendenfeldia *sp.* Using the Molecular Networking (MN) Approach

**Figure 2:**
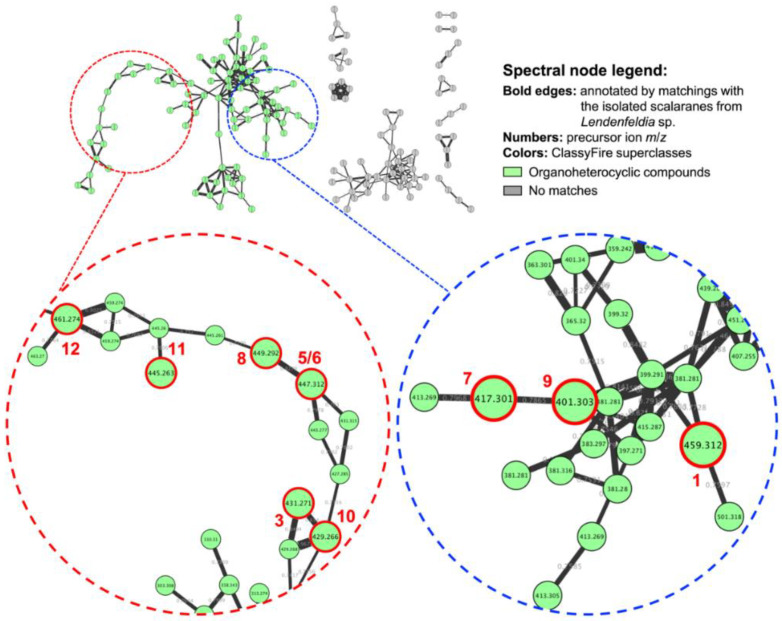
MS/MS molecular networking revealed the structural diversity of scalarane-type sesterterpenoids from marine sponge *Lendenfeldia* sp.

An ethyl acetate extract of freeze-dried *Lendenfeldia* sp. sponge was prepared and subjected to MS/MS analysis using LC-QTOF. The collected MS^2^ data were further processed on the Global Natural Product Social (GNPS) Molecular Networking platform, then the Cytoscape and MolNetEnhancer tools were applied to lay out the metabolomic profile. Manual annotations for these isolated scalaranes were performed according to the similarity of MS^2^ fragmentation using our in-house database. Previously-reported 24-homoscalarane **10** was found to be primarily present in the MN cluster characterizing organoheterocyclic compounds via ClassyFire analysis (Figure 2). In order to investigate the chemical diversity of minor scalarane derivatives, the ethyl acetate extract was further fractionated and purified using normal phase (NP) and reverse phase (RP) column chromatography and analyzed by LC-MS/MS. A series of scalaranes were traced and isolated, including seven new 24-homoscalaranes, lendenfeldaranes K–Q (**1**–**7**), and five known derivatives, lendenfeldarane B (**8**) [2], 25-nor-24-methyl-12,24-dioxoscalar-16-en-22-oic acid (**9**) [18], 24-methyl-12,24,25-trioxoscalar-16-en-22-oic acid (**10**) [11], felixin B (**11**) [19], and 23-hydroxy-20-methyldeoxoscalarin (**12**) [20].

### 2.2. Chemical Identification of 24-Homoscalaranes ***1–12***

**Figure 3:**
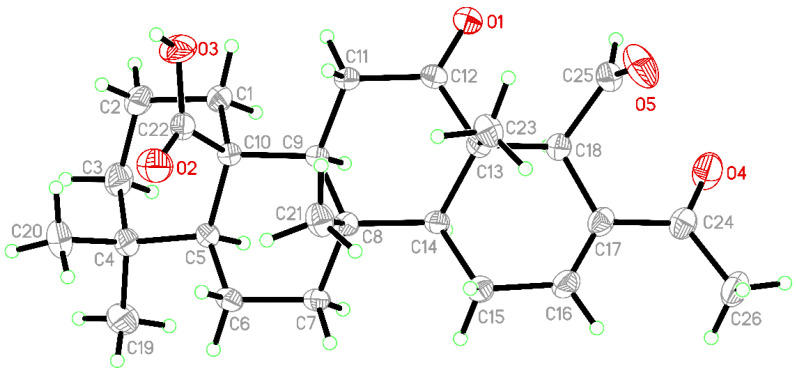
Crystal structure and absolute configuration of **10** by X-ray diffraction.

24-Methyl-12,24,25-trioxoscalar-16-en-22-oic acid (**10**) was first compound isolated from marine sponge *Lendenfeldia* sp. [11], and the structure of this compound was elucidated by spectroscopic and chemical methods. The structure, including the absolute configuration, of 10 was established in this study by single-crystal X-ray diffraction analysis (Flack parameter x = *−*0.05(5)), and an ORTEP diagram (Figure 3) showed the configurations of stereogenic carbons to be (5*S*,8*S*,9*S*,10*R*,13*S*,14*S*,18*S*).

Lendenfeldarane K (**1**) was obtained as an amorphous powder. The positive mode high resolution electrospray ionization mass spectrum [(+)-HRESIMS] peak at *m/z* 481.29259 (calcd. for C_28_H_42_O_5_ + Na, 481.29245) indicated a molecular formula of C_28_H_42_O_5_, implying eight degrees of unsaturation. The IR spectrum of **1** revealed the presence of ester carbonyl (ν_max_ 1738 cm^−1^) and hydroxy (ν_max_ 3466 cm^−1^) groups. The ^1^H NMR spectrum of **1** (Table 1) showed six methyls at δ_H_ 0.77, 0.87, 1.09, 1.19, 1.96 (each 3H × s), and 1.36 (3H, d, *J* = 6.6 Hz), and two oxymethine protons at δ_H_ 4.77 (1H, q, *J* = 6.6 Hz) and 5.50 (1H, dd, *J* = 3.0, 3.0 Hz). The anisochronous signals of geminal protons at δ_H_ 4.03 (1H, d, *J* = 12.0 Hz) and 3.87 (1H, dd, *J* = 12.0, 1.2 Hz) suggested the presence of an oxymethylene group. Analyses of the ^13^C (Table 1) and heteronuclear single quantum coherence (HSQC) spectra of **1** revealed the existence of 28 carbon resonances, including six methyls, nine methylenes (one oxygenated at δ_C_ 62.8), five methines (two oxygenated at δ_C_ 74.0 and 77.6), and eight non-protonated carbons (two olefin carbons at δ_C_ 132.7 and 163.6 and two ester carbonyls at δ_C_ 169.9 and 171.0). The above NMR data accounted for three degrees of unsaturation, implying a pentacyclic structure of **1**.

**Figure 4:**
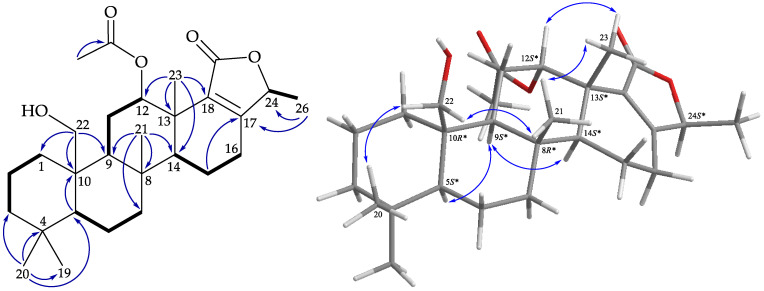
Key COSY (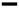), HMBC (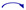), and protons with NOESY (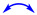) correlations of **1**.

The structure was further illustrated based on the 2D NMR spectra, including ^1^H–^1^H correlation spectroscopy (COSY) and heteronuclear multiple bond coherence (HMBC) spectra (Figure 4). The ^1^H–^1^H COSY spectra suggested five partial structures of consecutive proton spin systems, H_2_-1/H_2_-2/H_2_-3; H-5/H_2_-6/H_2_-7; H-9/H_2_-11/H-12; H-14/H_2_-15/H_2_-16; and H-24/H_3_-26. The HMBC cross-peaks among H_3_-20/C-3, C-4, C-5, C-19; H_3_-21/C-7, C-8, C-9, C-14; H_2_-22/C-1, C-9, C-10; H_3_-23/C-12, C-13, C-14, C-18; H_3_-26/C17, C-24; H-5/C10; and H_2_-15/C-17 connected the partial structures, resulting in the successful establishment of the gross structure of **1**. The above data resembled those of a known 24-homoscalarane, lendendfeldarane C (**13**) [2], except that the hydroxy group at C-12 in **13** (δ_H_ 4.60, 1H, brs/δ_C_ 69.9) was replaced by an acetoxy group in **1** (δ_H_ 5.50, 1H, dd*, J* = 3.0, 3.0 Hz/δ_C_ 74.0). From the findings of previous surveys, all naturally-occurring scalaranes have β-oriented Me-22 and Me-23 at C-10 and C-13, respectively [21]. Using C-10 and C-13 as the anchor points in this carbon skeleton, the orientations of Me-22 and Me-23 were consistent, regardless of the oxidation moieties (oxidation states) of these two methyls (-CH_2_OH, -CH_2_OAc, -COOH, and -CHO).

In the NOESY experiment of **1** (Figure 4), the acetoxy group at C-12 was assigned on the α-face, according to a NOESY correlation between H-12 and H_3_-23. It was found that the NOESY correlations of **1** were similar to those of **13**, suggesting close configurations of these two molecules. Therefore, lendenfeldarane K (**1**) was assigned as having a structure with the same relative stereochemistry as lendenfeldarane C (**13**) owing to the stereogenic carbons that **1** had in common with **13**, and the configurations of the stereogenic centers of **1** were elucidated as (5S*,8R*,9S*,10R*,12S*,13S*,14S*,24S*). Accordingly, the structure of **1** was established.

Additionally, scalaranes **1**–**12** were obtained from the same target organism, *Lendenfeldia* sp., and the absolute configuration of **10** was determined by single-crystal X-ray diffraction analysis. Therefore, it is biogenetically reasonable to conclude that **1**–**9**, **11**, and **12** have the same absolute configurations as **10**, and the stereogenic carbons of **1** were elucidated as (5*S*,8*R*,9*S*,10*R*,12*S*,13*S*,14*S*,24*S*).

**Figure 5:**
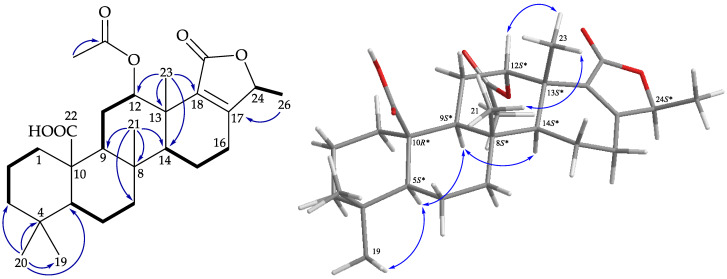
Key COSY (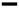), HMBC (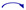), and protons with NOESY (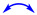) correlations of **2**.

Lendenfeldarane L (**2**) had a molecular formula of C_28_H_40_O_6_ at *m*/*z* 495.27187 (calcd. for C_28_H_40_O_6_ + Na, 495.27171) according to (+)-HRESIMS, corresponding to nine degrees of unsaturation. ^1^H and ^13^C data (Table 1) analyses indicated that **2** was of the 24-homo-scalarane class, which was similar to **1**. The most striking difference between **1** and **2** was the presence of signals assigned to the hydroxymethyl group (δ_H_ 4.03, 1H, d, *J* = 12.0 Hz; 3.87, 1H, d, *J* = 12.0, 1.2 Hz/δ_C_ 62.8, CH_2_-22) at C-10 in **1** being replaced by a carboxylic acid (δ_C_ 178.8) in **2**. Interpretation of the 2D NMR spectroscopic data of **2** confirmed the above elucidation, and thus established the planar structure (Figure 5). The correlations from the NOESY experiment of **2** indicated configurations of the stereogenic centers in core rings A–E of **2** that were identical to those of **1** (Figure 5). Therefore, the configurations of the stereogenic carbons of **2** were elucidated as (5*S*,8*S*,9*S*,10*R*,12*S*,13*S*,14*S*,24*S*). Accordingly, the structure of lendenfeldarane L (**2**) was established.

**Figure 6:**
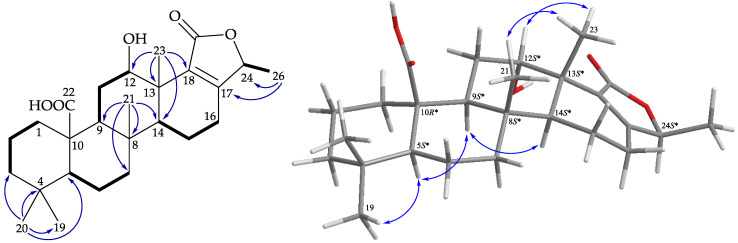
Key COSY (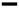), HMBC (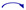), and protons with NOESY (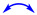) correlations of **3**.

Compound **3** was isolated as an amorphous powder, and the molecular formula was determined by (+)-HRESIMS as C_26_H_38_O_5_ at *m*/*z* 453.26085 (calcd. for C_26_H_38_O_5_ + Na, 453.26115). Comparison of the ^1^H and ^13^C NMR data of **3** (Table 2) with those of **2**, the chemical shifts of CH-12 in **2** (δ_H_ 5.56, dd, *J* = 2.8, 2.8 Hz/δ_C_ 73.6) being shifted up-field in **3** (δ_H_ 4.65, br s/δ_C_ 69.7), along with missing acetyl signals, suggesting that the 12-acetoxy group in **2** was replaced by a hydroxy group in **3**. Interpretation of the 2D NMR spectroscopic data of **3** confirmed the above elucidation, and thus established the planar structure (Figure 6). The configurations of the stereogenic centers in **3** were assigned as (5*S*,8*S*,9*S*,10*R*,12*S*,13*S*,14*S*,24*S*), the same as those in **2**, according to the NOESY spectrum (Figure 6). Thus, the structure of **3** was elucidated, and the compound was named lendenfeldarane M.

**Figure 7:**
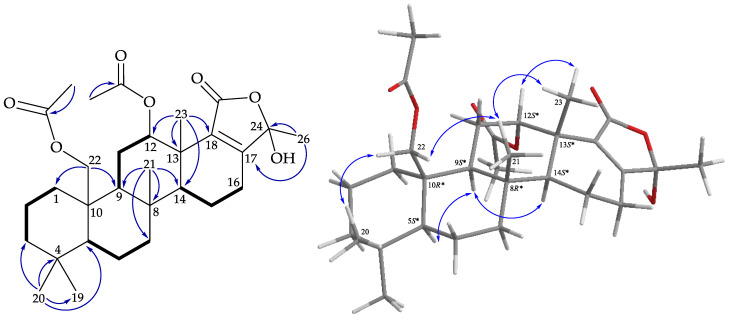
Key COSY (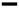), HMBC (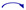), and protons with NOESY (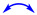) correlations of **4**.

Compound **4** was isolated as an amorphous powder, which was determined to have a molecular formula of C_30_H_44_O_7_ by (+)-HRESIMS at *m*/*z* 539.29763 (calcd. for C_30_H_44_O_7_ + Na, 539.29792), requiring nine degrees of unsaturation. Analysis of the 1D NMR data (Table 2) indicated that compound **4** was an analogue of a known 24-homoscalarane, lendenfeldarane D (**14**) (Figure 1) [2]. The main difference was the presence of an additional hydroxy group at C-24 in **4**, which was supported by MS data, less shielding of C-24 (from δ_C_ 77.7 to 103.4), and a combination of HMBC cross-peaks from H_3_-26 (δ_H_ 1.59, s) to C-24 (δ_C_ 103.4) and C-17 (δ_C_ 161.6) (Figure 7). The configuration of **4** was established by comparing the NOESY correlations (Figure 7) to those of **14**. The NOESY interactions of H_2_-22 with H_3_-20 and H_3_-21; and H_3_-23 with H-12 and H_3_-21, revealed the β-orientations of H_3_-20, H_3_-21, H_2_-22, H_3_-23, and H-12. H-5 showed an interaction with H-9, and H-9 was correlated with H-14, suggesting the α-orientations of H-5, H-9, and H-14. According to the above analyses, the structure of **4** was determined and the stereogenic centers were assigned as (5*S*,8*R*,9*S*,10*R*,12*S*,13*S*,14*S*). This compound was named lendenfeldarane N, although the stereochemistry of C-24 in **4** was not determined at this stage owing to the lack of a NOESY correlation between H_3_-24 and any protons.

**Figure 8:**
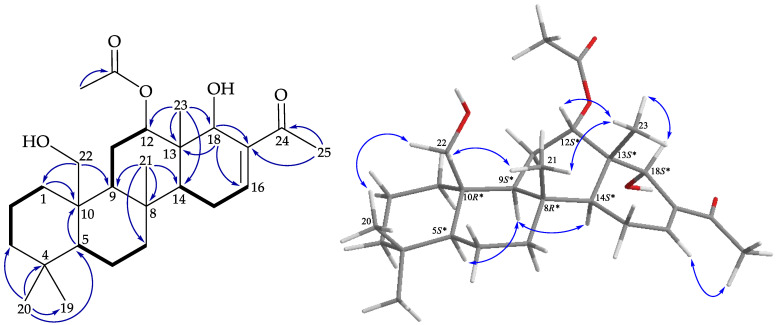
Key COSY (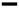), HMBC (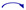), and protons with NOESY (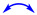) correlations of **5**.

Compound **5** was isolated as an amorphous powder, with the molecular formula C_27_H_42_O_5_ according to (+)-HRESIMS at *m*/*z* 469.29237 (calcd. for C_27_H_42_O_5_ + Na, 469.29245), corresponding to seven degrees of unsaturation. Based on the ^1^H and ^13^C NMR spectra (Table 3), **5** was found to possess an acetoxy (δ_H_ 2.14, 3H × s; δ_C_ 169.2, C; 22.0, CH_3_) and a ketonic carbonyl (δ_C_ 197.8) group. Additional unsaturated functionality was indicated by ^13^C resonances at δ_C_ 143.6 (CH) and 132.2 (C), suggesting the presence of a trisubstituted olefin. Thus, from the above data, three degrees of unsaturation were accounted for, and **5** was identified as a tetracyclic scalarane analogue. The NMR data of **5** resembled those of felixin A (**15**) [19], except for an additional oxymethine signal (δ_C_ 70.9; δ_H_ 4.38, 1H, s; CH-18). A hydroxy group substitution at C-18 was deduced from HMBC cross-peaks between H_3_-23/C-12, C-13, C-14, C-18 and H-18/C-13, C-16, C-17 (Figure 8). The correlations obtained from the NOESY experiment of **5** (Figure 8) showed that the configurations of the stereogenic centers in the core rings A–C in **5** were identical to those of **1**. The NOESY experiment showed correlations of H_3_-23 with H-12, H-18, and H_3_-21, suggesting the β-orientations of H-12 and H-18, and the stereogenic carbons were assigned as (5*S*,8*R*,9*S*,10*R*,12*S*,13*S*,14*S*,18*S*). According to the above analyses, the structure of **5** was determined, and the compound was named lendenfeldarane O.

**Figure 9:**
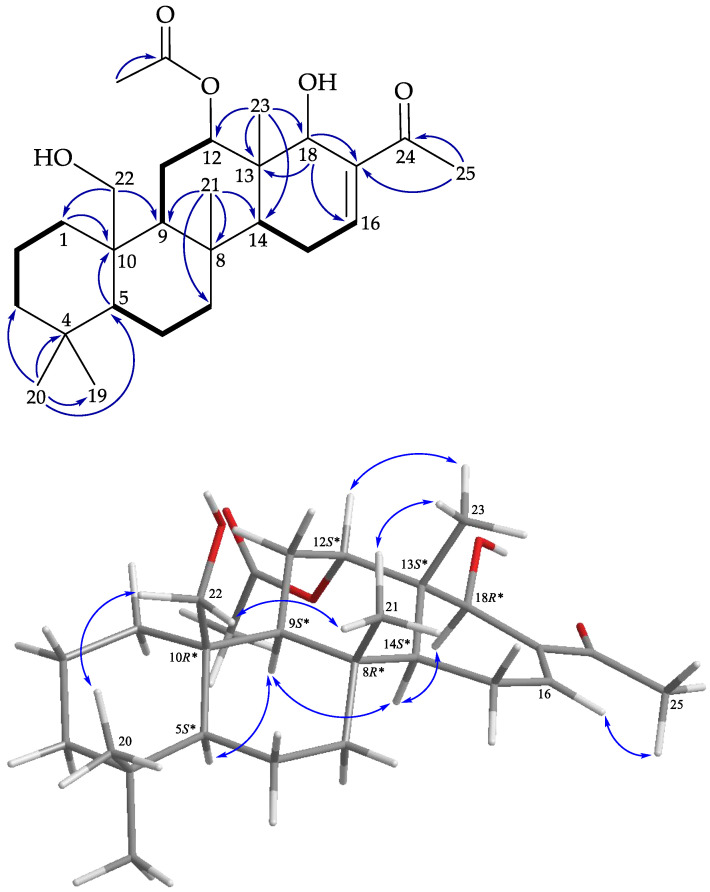
Key COSY (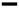), HMBC (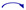), and protons with NOESY (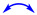) correlations of **6**.

Lendenfeldarane P (**6**) had a molecular formula of C_27_O_42_O_5_ at *m*/*z* 469.29237 (calcd. for C_27_O_42_O_5_ + Na, 469.29245), the same as that of **5**. The gross structure of **6** was established by interpretation of 1D and 2D NMR data, especially by analysis of the COSY and HMBC correlations (Figure 9). The ^1^H and ^13^C NMR data (Table 3) of **6** were found to be similar to those of **5**, except for C-17 and CH-18 resonating at δ_C_ 132.2 and 70.9 in **5**, and δ_C_ 140.0 and 69.7 in **6**, respectively, revealing that **6** was the 18R* isomer of **5**. The NOESY experiment of **6** (Figure 9) showed a NOESY correlation from H-14 to H-18, which suggested the α-orientation of H-18. According to the above analyses, the structure of lendenfeldarane P (**6**) was determined, and the stereogenic carbons of this compound were assigned as (5*S*,8*R*,9*S*,10*R*,12*S*,13*S*,14*S*,18*R*).

**Figure 10:**
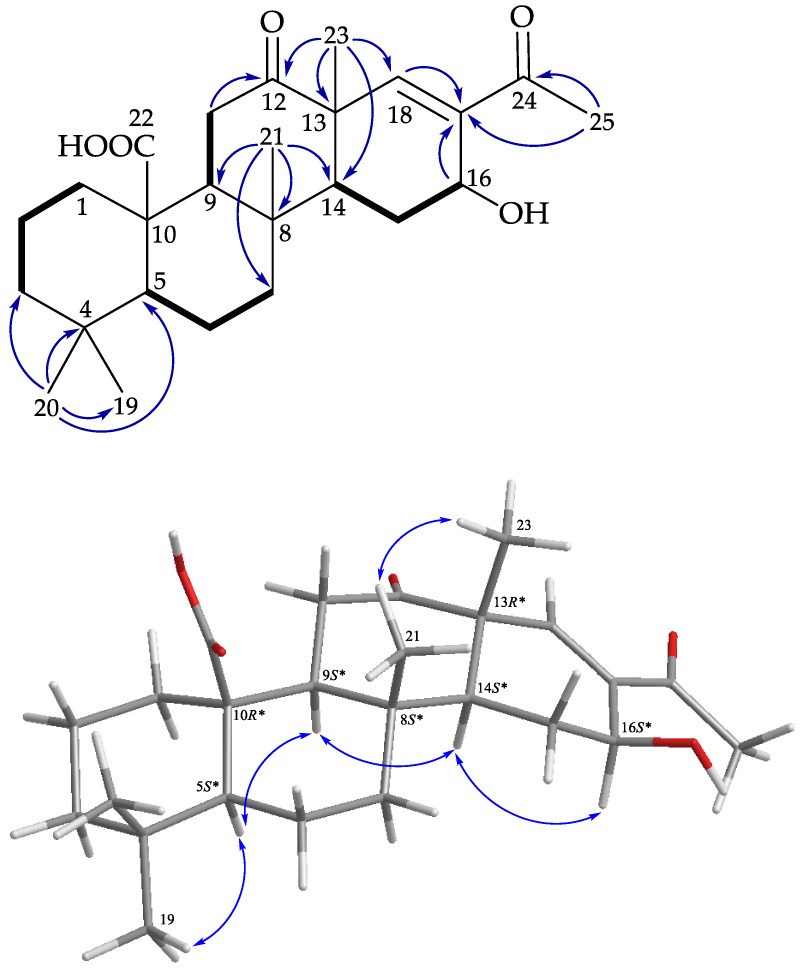
Key COSY (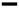), HMBC (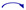), and protons with NOESY (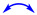) correlations of **7**.

Compound **7** was found to have the molecular formula C_25_H_36_O_5_, as deduced from a (+)-HRESIMS peak at *m*/*z* 439.24547 (calcd. for C_25_H_36_O_5_ + Na, 439.24550), revealing eight degrees of unsaturation. The ^13^C and distortionless enhancement by polarization transfer (DEPT) spectra (Table 4) showed 25 carbon signals, which were classified as five methyls, seven sp^3^ methylenes, four sp^3^ methines, four sp^3^ non-protonated carbons, one sp^2^ methine, and four sp^2^ non-protonated carbons. Based on the ^1^H and ^13^C spectra (Table 4), four degrees of unsaturation were accounted for, and the remaining four degrees were attributed to a tetracyclic ring. The consecutive COSY correlations (Figure 10) of H_2_-1/H_2_-2/H_2_-3, H-5/H_2_-6/H_2_-7, H-9/H_2_-11, and H-14/H_2_-15/H-16, in conjunction with HMBC correlations (Figure 10) from H_3_-20/C-3, C-4, C-5, C-19; H_3_-21/C-7, C-8, C-9, C-14; H_3_-23/C-12, C-13, C-14, C-18; H_2_-11/C-12; H-16/C-17; H-18/C-17; and H_3_-25/C-17, C-24, established **7** as a 6/6/6/6 tetracyclic nor-24-homoscalarane, bearing a carboxylic acid at C-10, a hydroxy group at C-16, an acetyl group at C-17, and a ketonic group at C-12. The NOESY interactions of H_3_-21 and H_3_-23 revealed the β-orientations of H_3_-21 and H_3_-23. H-5 showed interactions with H_3_-19 and H-9, H-9 showed an interaction with H-14, and H-14 showed an interaction with H-16, suggesting the α-orientations of H-5, H-9, H-14, and H-16. According to the above analyses, the stereogenic carbons of this compound were assigned as (5*S*,8*S*,9*S*,10*R*,13*R*,14*S*,16*S*); the structure of **7** was determined, and the compound was named lendenfeldarane Q.

### 2.3. Assessments of O_2_^•−^ Generation and Elastase Release in fMLF-Activated Human Neutrophils

Neutrophils can be induced by *N*-formyl-methionyl-leucyl-phenylalanine (fMLF), and pathogen-associated molecular patterns (PAMPs) lead to a series of inflammatory responses such as respiratory burst (O_2_^•−^ generation) and degranulation (elastase release) [22]. In order to evaluate the anti-inflammatory activities of scalaranes, all isolates (except for **5** owing to an amount limitation) were assayed in fMLF-induced human neutrophils (Table 5). Compound **1** exhibited the most significant activity against both O_2_^•−^ accumulation (IC_50_ = 0.87 μM) and elastase release (IC_50_ = 1.12 μM), while compound **4** was not active at a concentration of 10 μM. Similarly, compounds **2**, **6**, **8**, **9**, and **10** showed potential anti-inflammatory effects (IC_50_ 1.11~2.78 μM) in both assays. In addition, compounds **3**, **7**, **11**, and **12** selectively inhibited either superoxide anion generation or elastase release. Compound **3**, a 12-*O*-deacetyl derivative of **2**, was found to exhibit a much weakened anti-inflammatory activity in comparison with **2**, indicating that the bulky acetate at C-12 significantly enhanced the anti-inflammatory activity.

## 3. Materials and Methods

### 3.1. General Experimental Procedures

Optical rotation spectra were recorded on a JASCO P-1010 polarimeter (Jasco, Tokyo, Japan). A Waters SYNAPT G2 system (Waters, Milford, MA, USA) was utilized to collect MS^2^ data. Liquid chromatography was carried out using a Waters Acquity UPLC BEH C18 (1.7 µm, 2.1 mm × 150 mm) column (Waters, Milford, MA, USA). IR spectra were obtained with a Thermo Scientific Nicolet iS5 FT-IR (Thermo Scientific, Waltham, MA, USA) spectrophotometer. NMR spectra were obtained on JEOL ECZ 400S or 600R NMR spectrometers (Jeol, Tokyo, Japan), using the residual CHCl_3_ (*δ*_H_ 7.26 ppm) and CDCl_3_ (δ_C_ 77.0 ppm) signals as the internal standards for ^1^H and ^13^C NMR, respectively. The coupling constants (*J*) are presented in Hz. ESIMS and HRESIMS data were collected on a Bruker 7 Tesla solariX FTMS system (Bruker, Bremen, Germany). TLC was performed on plates coated with Kieselgel 60 F_254_ (0.25 mm, Merck, Darmstadt, Germany) and/or RP-18 F_254S_ (0.25 mm, Merck, Darmstadt, Germany) and then visualized by spraying with 10% H_2_SO_4_ and heating on a hot plate. Silica gel 60 (40~63 and 63~200 μm, Merck, Darmstadt, Germany) was used for column chromatography. Normal-phase HPLC (NP-HPLC) was performed using a system comprising a pump (L-7110, Hitachi, Tokyo, Japan), an injection port (Rheodyne, 7725, Rohnert Park, CA, USA), and a preparative normal-phase column (YMC-Pack SIL, SIL-06, 250 × 20 mm, D. S-5 μm; Sigma-Aldrich, St. Louis, MO, USA). Reverse-phase HPLC (RP-HPLC) was performed using a system comprising a pump (L-2130, Hitachi, Tokyo, Japan), a photodiode array detector (L-2455, Hitachi, Tokyo, Japan), an injection port (Rheodyne, 7725), and a reverse-phase column (Luna 5 μm, C18(2) 100Å AXIA Packed, 250 × 21.2 mm; Phenomenex, Torrance, CA, USA).

### 3.2. Animal Material and Isolation of Compounds

The specimen of *Lendenfeldia* sp. was collected by hand via self-contained underwater breathing apparatus (SCUBA) diving off the coast of Southern Taiwan in April 2019. A voucher specimen was deposited at the National Museum of Marine Biology & Aquarium, Taiwan (specimen No. 2019-04-SP). Taxonomic identification was performed by Prof. Yusheng M. Huang from the National Penghu University of Science and Technology, Taiwan. *Lendenfeldia* sp. (2.9 kg fresh weight) was collected and freeze-dried. The sponge material (213 g, dry weight) was minced and extracted exhaustively with a mixture of CH_2_Cl_2_:MeOH (1:1, 1L × 6). The extract was partitioned between EtOAc and H_2_O, then the EtOAc layer (7.9 g) was subjected to column chromatography on silica gel, and eluted with a gradient solvent system of *n*-hexane, *n*-hexane and EtOAc mixtures of increasing polarity, pure acetone, and finally pure methanol as eluents to yield 14 sub-fractions A–N. Fraction H was chromatographed on C18 silica gel and eluted using a mixture of MeOH/H_2_O (1:1 → pure MeOH) to afford six sub-fractions H1–H6. Fraction H4 was subjected to NP-HPLC with an isocratic solvent system of an *n*-hexane/acetone mixture (5:1; flow rate = 3.0 mL/min) to afford 10 sub-fractions H4A–H4J, including **7** (5.1 mg). Fraction H4G was separated by RP-HPLC using an isocratic solvent system of a MeOH/H_2_O mixture (4:1; flow rate = 5.0 mL/min) to afford **2** (0.7 mg) and **3** (1.5 mg). Fraction H5 was separated by RP-HPLC using an isocratic solvent system of a MeOH/H_2_O mixture (4:1; flow rate = 5.0 mL/min) to afford 13 sub-fractions H5A–H5M, including **1** (1.3 mg), **4** (4.8 mg) and **6** (2.2 mg). Fraction H5M was separated by RP-HPLC using an isocratic solvent system of a MeOH/H_2_O mixture (17:3; flow rate = 5.0 mL/min) to afford **5** (0.5 mg) and **12** (2.0 mg). Fraction I was chromatographed on silica gel and eluted using *n*-hexane/acetone (8:1–pure acetone) to afford eight sub-fractions I1–I8. Fraction I5 was separated by NP-HPLC using an isocratic solvent system of *n*-hexane/acetone (5:2; flow rate = 3.0 mL/min) to afford 10 sub-fractions I5A–I5J, including **10** (57.0 mg). Fraction I5C was separated by RP-HPLC using an isocratic solvent system of MeOH/H_2_O (4:1; flow rate = 5 mL/min) to afford **8** (2.5 mg), **9** (0.8 mg), and **11** (7.8 mg).

Lendenfeldarane K (**1**): Amorphous powder; [α]${}_{D}^{25}$ +66 (*c* 0.24, CHCl_3_); IR (ATR) ν_max_ 3466, 1738 cm^−1^; ^1^H (600 MHz, CDCl_3_) and ^13^C (150 MHz, CDCl_3_) NMR spectroscopic data, Table 1; ESIMS *m/z* 481 [M + Na]^+^; HRESIMS *m*/*z* 481.29259 [M + Na]^+^ (calcd. for C_28_H_42_O_5_ + Na, 481.29245).

Lendenfeldarane L (**2**): Amorphous powder; [α]${}_{D}^{25}$ +42 (*c* 0.04, CHCl_3_); IR (ATR) ν_max_ 3575~2350 (broad), 1736, 1701, 1673 cm^−1^; ^1^H (400 MHz, CDCl_3_) and ^13^C (100 MHz, CDCl_3_) NMR spectroscopic data, Table 1; ESIMS *m/z* 495 [M + Na]^+^; HRESIMS *m*/*z* 495.27187 [M + Na]^+^ (calcd. for C_28_H_40_O_6_ + Na, 495.27171).

Lendenfeldarane M (**3**): Amorphous powder; [α]${}_{D}^{25}$ +76 (*c* 0.04, CHCl_3_); IR (ATR) ν_max_ 3593~2404 (broad), 3439, 1727, 1700 cm^−1^; ^1^H (400 MHz, CDCl_3_) and ^13^C (100 MHz, CDCl_3_) NMR spectroscopic data, Table 2; ESIMS *m/z* 453 [M + Na]^+^; HRESIMS *m*/*z* 453.26085 [M + Na]^+^ (calcd. for C_26_H_38_O_5_ + Na, 453.26115).

Lendenfeldarane N (**4**): Amorphous powder; [α]${}_{D}^{25}$ +83 (*c* 0.05, CHCl_3_); IR (ATR) ν_max_ 3421, 1737 cm^−1^; ^1^H (400 MHz, CDCl_3_) and ^13^C (100 MHz, CDCl_3_) NMR spectroscopic data, Table 2; ESIMS *m/z* 539 [M + Na]^+^; HRESIMS *m*/*z* 539.29763 [M + Na]^+^ (calcd. for C_30_H_44_O_7_ + Na, 539.29792).

Lendenfeldarane O (**5**): Amorphous powder; [α]${}_{D}^{25}$ +17 (*c* 0.03, CHCl_3_); IR (ATR) ν_max_ 3508, 1715, 1667 cm^−1^; ^1^H (600 MHz, CDCl_3_) and ^13^C (150 MHz, CDCl_3_) NMR spectroscopic data, Table 3; ESIMS *m/z* 469 [M + Na]^+^; HRESIMS *m*/*z* 469.29237 [M + Na]^+^ (calcd. for C_27_H_42_O_5_ + Na, 469.29245).

Lendenfeldarane P (**6**): Amorphous powder; [α]${}_{D}^{25}$ +36 (*c* 0.11, CHCl_3_); IR (ATR) ν_max_ 3447, 1714, 1644 cm^−1^; ^1^H (600 MHz, CDCl_3_) and ^13^C (150 MHz, CDCl_3_) NMR spectroscopic data, Table 3; ESIMS *m/z* 469 [M + Na]^+^; HRESIMS *m*/*z* 469.29237 [M + Na]^+^ (calcd. for C_27_H_42_O_5_ + Na, 469.29245).

Lendenfeldarane Q (**7**): Amorphous powder; [α]${}_{D}^{25}$ +58 (*c* 0.26, CHCl_3_); IR (ATR) ν_max_ 3695~2473 (broad), 3408, 1703, 1661 cm^−1^; ^1^H (400 MHz, CDCl_3_) and ^13^C (100 MHz, CDCl_3_) NMR spectroscopic data, Table 4; ESIMS *m/z* 439 [M + Na]^+^; HRESIMS *m*/*z* 439.24547 [M + Na]^+^ (calcd. for C_25_H_36_O_5_ + Na, 439.24550).

### 3.3. X-ray Crystallographic Analysis of 24-Methyl-12,24,25-trioxoscalar-16-en-22-oic Acid (***10***)

Crystallographic data of 10 were obtained at 200(2) K on a Bruker D8 VENTURE single-crystal XRD system equipped with Oxford Cryostream 800^+^ with Cu Kα radiation (wavelength = 1.54178 Å). These data for the structure of 24-methyl-12,24,25-trioxoscalar-16-en-22-oic acid (**10**) were deposited with the Cambridge Crystallographic Data Center under supplementary publication number CCDC 1988979 at 9 March 2020 and can be obtained free of charge via http://www.ccdc.cam.ac.uk/conts/retrieving.html (accessed on 20 July 2021).

Crystallographic data of 10*:* Suitable colorless prisms of 10 were obtained from a solution of MeOH. The crystal (0.271 × 0.126 × 0.123 mm^3^) belongs to the monoclinic system, space group *P*2_1_ (#4), with *a* = 7.6278(3) Å, *b* = 10.3477(4) Å, *c* = 14.45181(5) Å, *α* = 90°, *β* = 103.9600(9)°, *γ* = 90°, *V* = 1107.47(7) Å^3^, *Z* = 2, *D*_calcd_ = 1.285 Mg/m^3^, and λ (Cu Kα) = 1.54178 Å. The total number of independent reflections measured was 4481, of which 4445 were observed [R(*int*) = 0.0224]. Completeness to *θ* = 67.679°: 99.8%, absorption correction: semi-empirical from equivalents, max. and min. transmission: 0.7539 and 0.5033. The structure was solved by direct methods and refined by a full-matrix least-squares procedure on *F^2^*. Final *R* indices [*I* > 2σ(*I*)]: *R1* = 0.0317, w*R2* = 0.0866. The absolute configuration was determined by Flack parameter x = −0.05(5) [23].

### 3.4. MS/MS Fragmentations Collection Using Ultra-Performance Liquid Chromatography Quadrupole Time-of-Flight (UPLC-QTOF) Mass Spectrometry

A Waters SYNAPT G2 LC/Q-TOF (Waters Corporation, Milford, MA, USA) system was utilized to collect MS^2^ data. Liquid chromatography was carried out using a Waters Acquity UPLC BEH C18 (1.7 µm, 2.1 mm × 150 mm) column (Waters). The mobile phase was prepared by mixing a MeCN (A, containing 0.1% formic acid) and water (W, containing 0.1% formic acid) gradient sequence as follows: 0–1 min, 5% A; 1–16 min, 5–99.5% A; 16–26 min, 99.5% A; 26–26.1 min, 99.5–5% A; 26.1–28 min, 5% A. The flow rate was fixed at 0.4 mL/min, and the column temperature was maintained at 40 °C. To prepare the sample, 4-mg extracts were dissolved in 1 mL of methanol (4000 ppm) and filtered through a 0.22 μm membrane filter prior to loading into the LC column. The sample injection was implemented automatically with a 5 μL volume per injection. The MS^1^ and MS^2^ data were collected within the range of *m/z* 100–2000. The automated data-dependent acquisition (DDA) mode was executed in the MS^2^ scans, and non-targeted selections of 5 precursor ions were fragmented with ramping of the collision energy from 10–50 V. The acquired MS data were processed using Waters MassFragment software (MassLynx4.1, Waters, Milford, MA, USA).

### 3.5. Chromatographic and Spectral Preprocessing Using MZmine

Raw MS/MS data files were imported into MZmine 2.33 (MZmine 2.33, Whitehead Institute for Biomedical Research, Cambridge, MA, USA). Mass detection was performed with the noise level at 200 (for MS scans) and 20 (for MS/MS scans). MS chromatograms were built with ions showing a minimum time span of 0.02 min, minimum height of 5000, and *m/z* tolerance of 0.002 (or 5.0 ppm), then missing data points were filled using the peak extender module with a minimum height of 1000. Chromatograms were deisotoped using the isotopic peaks grouper algorithm with an *m/z* tolerance of 0.002 (or 10.0 ppm) and a t*_R_* tolerance of 0.1 min, and then aligned together into a peak table in the join aligner module. Peaks without any MS/MS scans were removed by the GNPS filter module, then gap-filled with the peak finder module.

### 3.6. GNPS Molecular Networking

MS/MS molecular networking was performed using the GNPS web platform (https://gnps.ucsd.edu) at 25 June 2021. MS/MS spectra were window-filtered by choosing the top 3 peaks in the ±50 Da window throughout the spectrum. A network was then created, in which edges were filtered to have a cosine score above 0.70 and more than four matched peaks. The spectra in the network were annotated based on the experimental MS^2^ fragmentations of isolated scalaranes. The library spectra were filtered in the same manner as the input data. The molecular network was visualized and presented using Cytoscape 3.8.2 (Cytoscape 3.8.2, NRNB, San Diego, La Jolla, CA, USA).

### 3.7. Preparation of Human Neutrophils

Blood was acquired from human donors (20~30 years old) by venipuncture under the approval and supervision of the Institutional Review Board (IRB) at Chang Gung Memorial Hospital. Neutrophils were purified utilizing a protocol of dextran sedimentation, hypotonic lysis, and Ficoll Hypaque gradient of erythrocytes according to previous reported methods [22]. Isolated human neutrophils were suspended in a calcium (Ca^2+^)-free HBSS buffer at pH 7.4 and examined by the trypan blue exclusion method (>98% viable cells). Then, neutrophil assessments were performed in HBSS containing 1 mM CaCl_2_ at 37 °C.

### 3.8. Measurement of Superoxide Anion (O_2_^•−^) Generation

O_2_^•−^ generation was assessed using superoxidase dismutase (SOD) inhibitable reduction of ferricytochrome *c* [22]. After supplementation with ferricytochrome *c* (0.6 mg/mL), neutrophils (6 × 10^5^ cells/mL) were equilibrated at 37 °C and incubated for 5 min before treatment with pure compounds or DMSO (0.1%, control). Since cytochalasin B (CB) can convert neutrophils from phagocytic into secretory cells and facilitate respiratory burst and degranulation through disaggregation of intracellular actin network [24], it was then added (1 μg/mL) to magnify the reaction and the mixture was left for 3 min after activation with 0.1 μM fMLF. Absorbance changes with reduction of ferricytochrome *c* were monitored continuously at 550 nm using a spectrophotometer (U-3010, Hitachi, Tokyo, Japan).

### 3.9. Measurement of Elastase Release

Degranulation of azurophilic granules was evaluated using an elastase release assay and performed using MeO-Suc-Ala-Ala-Pro-Val-p-nitroanilide as the elastase substrate [1]. In brief, neutrophils (6 × 10^5^ cells/mL) were equilibrated at 37 °C after supplementation with MeO-Suc-Ala-Ala-Pro-Val-p-nitroanilide (100 μM), then incubated for 5 min before treatment with pure compounds. CB (0.5 g/mL) was added to magnify the reaction, followed by the addition of fMLF (0.1 μM) to induce cell activation. The variations in absorbance at 405 nm were monitored continuously to assess elastase release.

### 3.10. Statistics

The results were expressed as the mean ± standard deviation (SD). Comparison in each experiment was performed using an unpaired Student’s *t*-test, and a *p* value of less than 0.05 was considered statistically significant.

## 4. Conclusions

The current study revealed the MN-based metabolomic profile of marine sponge *Lendenfeldia* sp. for the first time, which indicated a splendid diversity of scalarane-type sesterterpenoids. Subsequent isolation of compounds of this type led to the identification of seven new 24-homoscalaranes, lendenfeldaranes K–Q (**1**–**7**), together with five known derivatives. Anti-neutrophilic assessments of these isolates not only revealed their great anti-inflammatory potential towards activated neutrophils, but also highlighted the bioactivity-relevant structural importance of the functional group at C-12 in the 24-homoscalarane analogues. These results indicate a great potential of this class of compounds for further development as anti-neutrophilic agents, especially the accomplishment in the synthetic protocol of a scalarane-type sesterterpenoid (16-deacetoxy-12-*epi*-scalarafuranacetate) [25], which could give sustainable supply for future industrial development.

## Figures and Tables

**Table 1:** ^1^H and ^13^C NMR data for 24-homoscalaranes **1** and **2**.

	1		2	
Position	δ_H_ (*J* in Hz) ^a^	δ_C_ Mult. ^b^	δ_H_ (*J* in Hz) ^c^	δ_C_ Mult. ^d^
1	2.12 m; 0.50 ddd (13.2, 13.2, 2.4)	34.3, CH_2_	2.49 m; 0.67 ddd (12.8, 12.8, 3.2)	38.4, CH_2_
2	1.54 m	18.3, CH_2_	1.60 m	18.6, CH_2_
3	1.42 m; 1.15 m	41.7, CH_2_	1.39 m; 1.14 m	42.2, CH_2_
4		33.0, C		33.3, C
5	0.96 m	57.0, CH	1.01 m	56.4, CH
6	1.94 m; 1.57 m	17.0, CH_2_	1.93 m; 1.52 m	16.9, CH_2_
7	1.93 m; 1.07 m	42.0, CH_2_	1.94 m; 1.05 ddd (12.8, 12.8, 2.8)	41.4, CH_2_
8		37.5, C		37.7, C
9	1.25 m	53.4, CH	1.45 m	52.4, CH
10		41.8, C		47.6, C
11	2.33 m; 2.17 m	23.9, CH_2_	2.30 m; 1.83 m	22.7, CH_2_
12	5.50 dd (3.0, 3.0)	74.0, CH	5.56 dd (2.8, 2.8)	73.6, CH
13		38.4, C		38.2, C
14	1.56 m	51.2, CH	1.51 m	50.9, CH
15	2.35 m	24.0, CH_2_	2.34 m; 2.20 m	23.9, CH_2_
16	1.57 m; 1.44 m	17.9, CH_2_	1.48 m	20.2, CH_2_
17		163.6, C		163.7, C
18		132.7, C		132.5, C
19	0.87 s	33.8, CH_3_	0.92 s	33.8, CH_3_
20	0.77 s	21.8, CH_3_	0.86 s	22.4, CH_3_
21	1.09 s	16.3, CH_3_	0.85 s	14.4, CH_3_
22	4.03 d (12.0); 3.87 dd (12.0, 1.2)	62.8, CH_2_		178.8, C
23	1.19 s	21.1, CH_3_	1.13 s	21.2, CH_3_
24	4.77 q (6.6)	77.6, CH	4.77 q (6.8)	77.7, CH
25		171.0, C		170.9, C
26	1.36 d (6.6)	18.7, CH_3_	1.35 d (6.8)	18.6, CH_3_
OAc-12		169.9, C		169.8, C
	1.96 s	21.2, CH_3_	1.96 s	21.2, CH_3_

^a^ 600 MHz in CDCl_3_, ^b^ 150 MHz in CDCl_3_, ^c^ 400 MHz, CDCl_3_, ^d^ 100 MHz, CDCl_3_.

**Table 2:** ^1^H and ^13^C NMR data for 24-homoscalaranes **3** and **4**.

	3		4	
Position	δ_H_ (*J* in Hz) ^a^	δ_C_ Mult. ^b^	δ_H_ (*J* in Hz) ^a^	δ_C_ Mult. ^b^
1	2.52 m; 0.94 m	38.3, CH_2_	2.01 m; 0.51 ddd (12.4, 12.4, 4.4)	34.7, CH_2_
2	2.30 m; 1.60 m	16.6, CH_2_	1.58 m	18.2, CH_2_
3	1.41 m; 1.16 m	42.2, CH_2_	1.44 m; 1.13 m	41.5, CH_2_
4		33.4, C		33.0, C
5	1.12 m	56.1, CH	1.01 m	57.1, CH
6	1.91 m; 1.54 m	18.6, CH_2_	1.58 m; 1.45 m	18.0, CH_2_
7	1.92 m; 1.10 m	41.3, CH_2_	1.93 m; 1.11 m	41.8, CH_2_
8		37.8, C		37.4, C
9	1.77 m	51.5, CH	1.31 m	53.2, CH
10		47.8, C		40.1, C
11	1.99 m; 1.89 m	25.8, CH_2_	2.20 m; 2.03 m	23.3, CH_2_
12	4.65 br s	69.7, CH	5.49 br s	73.9, CH
13		40.1, C		38.5, C
14	1.55 m	49.8, CH	1.53 m	51.3, CH
15	2.26 m	24.3, CH_2_	2.48 m; 2.35 m	22.8, CH_2_
16	1.53 m; 1.42 m	20.2, CH_2_	1.42 m	18.1, CH_2_
17		165.4, C		161.6, C
18		133.1, C		134.3, C
19	0.92 s	33.7, CH_3_	0.88 s	33.7, CH_3_
20	0.88 s	22.4, CH_3_	0.83 s	21.8, CH_3_
21	0.85 s	14.3, CH_3_	0.97 s	16.4, CH_3_
22		179.3, C	4.58 d (12.0); 4.14 d (12.0)	64.7, CH_2_
23	1.09 s	21.5, CH_3_	1.21 s	21.1, CH_3_
24	4.78 q (6.8)	78.6, CH		103.4, C
25		172.5, C		178.1, C
26	1.36 d (6.8)	18.4, CH_3_	1.59 s	24.3, CH_3_
OAc-12				169.6, C
			1.95 s	21.2, CH_3_
OAc-22				171.0, C
			2.06 s	21.2, CH_3_

^a^ 400 MHz in CDCl_3_, ^b^ 100 MHz in CDCl_3_.

**Table 3:** ^1^H and ^13^C NMR data for 24-homoscalaranes **5** and **6**.

	5		6	
Position	δ_H_ (*J* in Hz) ^a^	δ_C_ Mult. ^b^	δ_H_ (*J* in Hz) ^a^	δ_C_ Mult. ^b^
1	2.11 m; 0.53 ddd (14.4, 14.4, 4.8)	34.3, CH_2_	2.12 m; 0.53 ddd (13.2, 13.2, 2.4)	34.4, CH_2_
2	1.54 m; 1.39 m	18.0, CH_2_	1.53 m	17.8, CH_2_
3	1.44 m; 1.20 m	41.7, CH_2_	1.45 m; 1.18 m	41.7, CH_2_
4		33.0, C		33.0, C
5	1.02 dd (12.6, 2.4)	56.7, CH	0.98 dd (12.6, 2.4)	56.9, CH
6	1.47 m	18.2, CH_2_	1.55 m; 1.48 m	18.4, CH_2_
7	1.82 ddd (12.6, 3.0, 3.0); 1.20 m	41.9, CH_2_	1.80 ddd (12.6, 3.0, 3.0); 1.06 m	41.9, CH_2_
8		37.2, C		37.4, C
9	1.35 br d (12.6)	53.0, CH	1.34 m	52.5, CH
10		41.7, C		41.7, C
11	2.20 m; 1.98 m	26.0, CH_2_	2.10 m; 2.00 m	25.0, CH_2_
12	4.92 dd (3.0, 3.0)	79.7, CH	5.01 dd (3.6, 2.4)	73.6, CH
13		39.2, C		40.1, C
14	2.23 m	42.0, CH	1.52 m	47.8, CH
15	1.25 m	29.6, CH_2_	2.26 m	24.3, CH_2_
16	6.99 br s	143.6, CH	6.88 br s	141.8, CH
17		132.2, C		140.0, C
18	4.38 s	70.9, CH	4.57 s	69.7, CH
19	0.89 s	33.8, CH_3_	0.87 s	33.8, CH_3_
20	0.77 s	22.0, CH_3_	0.77 s	21.9, CH_3_
21	1.11 s	15.5, CH_3_	1.15 s	15.8, CH_3_
22	4.05 d (10.8); 3.91 dd (10.8, 4.8)	62.9, CH_2_	4.04 d (12.0); 3.89 dd (12.0, 1.2)	63.0, CH_2_
23	0.70 s	19.7, CH_3_	0.94 s	12.6, CH_3_
24		197.8, C		202.2, C
25	2.32 s	24.8, CH_3_	2.32 s	26.1, CH_3_
OAc-12		169.2, C		170.1, C
	2.14 s	22.0, CH_3_	2.11 s	21.5, CH_3_
18-OH			4.11 d (1.8)	

^a^ 600 MHz in CDCl_3_, ^b^ 150 MHz in CDCl_3_.

**Table 4:** ^1^H and ^13^C NMR data for 24-homoscalarane **7**.

Position	δ_H_ (*J* in Hz) ^a^	δ_C_ Mult. ^b^
1	2.47 m; 0.91 m	38.3, CH_2_
2	1.54 m	20.1, CH_2_
3	1.40 m; 1.16 m	42.0, CH_2_
4		33.4, C
5	1.06 m	56.2, CH
6	2.31 m; 1.65 m	18.4, CH_2_
7	2.00 m; 1.13 m	40.6, CH_2_
8		37.3, C
9	1.61 m	58.5, CH
10		48.7, C
11	2.87 dd (14.4, 14.4); 2.67 dd (14.4, 2.8)	36.5, CH_2_
12		210.3, C
13		50.3, C
14	1.70 m	48.7, CH
15	1.96 m; 1.73 m	25.1, CH_2_
16	4.57 dd (4.0, 1.6)	63.2, CH
17		137.7, C
18	7.40 s	147.2, CH
19	0.91 s	33.7, CH_3_
20	0.88 s	22.5, CH_3_
21	1.03 s	14.3, CH_3_
22		179.3, C
23	1.20 s	19.8, CH_3_
24		201.8, C
25	2.36 s	25.7, CH_3_

^a^ 400 MHz in CDCl_3_, ^b^ 100 MHz in CDCl_3_.

**Table 5:** Anti-inflammatory activities of isolated compounds **1**–**4** and **6**–**12**.

Compound	Superoxide Anion generation		Elastase Release	
	IC_50_ (μM) ^a^	Inh %	IC_50_ (μM) ^a^	Inh %
**1**	0.87 ± 0.14	98.90 ± 0.79 ***	1.12 ± 0.37	101.69 ± 2.91 ***
**2**	1.11 ± 0.10	101.77 ± 1.05 ***	1.65 ± 0.31	91.46 ± 5.24 ***
**3**	6.57 ± 0.67	70.78 ± 5.80 ***		48.78 ± 3.17 ***
**4**		34.63 ± 6.30 **		42.52 ± 5.88 **
**6**	1.75 ± 0.02	97.57 ± 1.42 ***	1.59 ± 0.41	102.08 ± 0.44 ***
**7**	6.25 ± 1.17	71.74 ± 8.89 ***		45.82 ± 6.54 **
**8**	1.47 ± 0.24	101.35 ± 0.77 ***	2.78 ± 0.78	101.83 ± 2.28 ***
**9**	1.50 ± 0.08	97.27 ± 1.59 ***	1.74 ± 0.15	101.80 ± 3.06 ***
**10**	2.83 ± 0.63	98.94 ± 0.60 ***	1.66 ± 0.09	93.47 ± 4.73 ***
**11**	6.33 ± 0.89	69.61 ± 6.49 ***		48.06 ± 5.55 ***
**12**		44.48 ± 6.95 **	6.97 ± 1.01	101.69 ± 8.13 ***

Percentage of inhibition (Inh %) at 10 μM. Results are presented as the mean ± S.E.M. (*n* = 3~5). ** *p* < 0.01, *** *p* < 0.001 compared with the control (DMSO). ^a^ Concentration necessary for 50% inhibition (IC_50_).

## Data Availability

The data presented in this study are available in article and Supplementary Materials.

## Supplementary Materials

The following are available online at https://www.mdpi.com/article/10.3390/md19100561/s1, Figures S1–S66: IR, ESIMS, HRESIMS, 1D-, and 2D-NMR spectra of compounds **1**–**7**.
