# Supplementary material for: Scalarane-Type Sesterterpenoids from the Marine Sponge Lendenfeldia sp. Alleviate Inflammation in Human Neutrophils
Source: Mar Drugs. 2021 Sep 30;19(10):561. doi: 10.3390/md19100561 (PMC8541400; doi:10.3390/md19100561)
Supplement: Supplementary file 1 [file marinedrugs-19-00561-s001.zip › marinedrugs-1393136-supplementary.pdf]

Supplementary Materials

Scalarane-type sesterterpenoids from the marine sponge *Lendenfeldia* sp. alleviate inflammation in human neutrophils

Bo-Rong Peng [1,2,†], Kuei-Hung Lai [3,4,5,†], Gene-Hsiang Lee [6], Steve Sheng-Fa Yu [7], Chang-Yih Duh [8], Jui-Hsin Su [2,8], Li-Guo Zheng [2], Tsong-Long Hwang [9,10,11,12,*], Ping-Jyun Sung [2,8,13,14,15,]**

1 Doctoral Degree Program in Marine Biotechnology, National Sun Yat-sen University, Kaohsiung, 804201, Taiwan;
2 National Museum of Marine Biology & Aquarium, Pingtung 944401, Taiwan
3 PhD Program in Clinical Drug Development of Herbal Medicine, College of Pharmacy, Taipei Medical University, Taipei 110301, Taiwan
4 Graduate Institute of Pharmacognosy, College of Pharmacy, Taipei Medical University, Taipei 110301,Taiwan
5 Traditional Herbal Medicine Research Center, Taipei Medical University Hospital, Taipei 110301, Taiwan
6 Instrumentation Center, National Taiwan University, Taipei 106319, Taiwan; ghlee@ntu.edu.tw
7 Institute of Chemistry, Academia Sinica, Taipei 115201, Taiwan; sfyu@chem.sinica.edu.tw
8 Department of Marine Biotechnology and Resources, National Sun Yat-sen University, Kaohsiung 804201, Taiwan;
9 Research Center for Chinese Herbal Medicine; Graduate Institute of Health Industry Technology, College of Human Ecology, Chang Gung University of Science and Technology, Taoyuan 333324, Taiwan
10 Department of Anaesthesiology, Chang Gung Memorial Hospital, Taoyuan 333423, Taiwan
11 Graduate Institute of Natural Products, College of Medicine, Chang Gung University, Taoyuan 333323, Taiwan
12 Department of Chemical Engineering, Ming Chi University of Technology, New Taipei City 243303, Taiwan
13 Chinese Medicine Research and Development Center, China Medical University Hospital, Taichung 404394, Taiwan
14 Graduate Institute of Natural Products, Kaohsiung Medical University, Kaohsiung 807378, Taiwan
15 PhD Program in Pharmaceutical Biotechnology, Fu Jen Catholic University, New Taipei 242062, Taiwan

† These authors contributed equally to this work

* Correspondence: htl@mail.cgu.edu.tw (T.-L.H.); pjsung@nmmba.gov.tw (P.-J.S.); Tel: +886-3-211-8800 (ext. 5523) (T.-L.H.); +886-8-882-5037 (P.-J.S.)

Table of Contents

Figure S1. IR spectrum of compound **1**........ 6

Figure S2. ESIMS spectrum of compound **1**........ 6

Figure S3. HRESIMS spectrum of compound **1**........ 7

Figure S4. ^{1}H NMR spectrum (600 MHz) of compound **1** in $CDCl_3$........ 7

Figure S5. ^{13}C NMR spectrum (150 MHz) of compound **1** in $CDCl_3$........ 8

Figure S6. HSQC spectrum of compound **1** in $CDCl_3$........ 8

Figure S7. HMBC spectrum of compound **1** in $CDCl_3$........ 9

Figure S8. ^{1}H-^{1}H COSY spectrum of compound **1** in $CDCl_3$........ 9

Figure S9. NOESY spectrum of compound **1** in $CDCl_3$........ 10

Figure S10. IR spectrum of compound **2**........ 10

Figure S11. ESIMS spectrum of compound **2**........ 11

Figure S12. HRESIMS spectrum of compound **2**........ 11

Figure S13. ^{1}H NMR spectrum (400 MHz) of compound **2** in $CDCl_3$........ 12

Figure S14. ^{13}C NMR spectrum (100 MHz) of compound **2** in $CDCl_3$........ 12

Figure S15. DEPT spectrum of compound **2** in $CDCl_3$........ 13

Figure S16. HSQC spectrum of compound **2** in $CDCl_3$........ 13

Figure S17. HMBC spectrum of compound **2** in $CDCl_3$........ 14

Figure S18. ^{1}H-^{1}H COSY spectrum of compound **2** in $CDCl_3$........ 14

Figure S19. NOESY spectrum of compound **2** in $CDCl_3$........ 15

Figure S20. IR spectrum of compound **3**. 15

Figure S21. ESIMS spectrum of compound **3**. 16

Figure S22. HRESIMS spectrum of compound **3**. 16

Figure S23. ^{1}H NMR spectrum (400 MHz) of compound **3** in $CDCl_3$. 17

Figure S24. ^{13}C NMR spectrum (100 MHz) of compound **3** in $CDCl_3$. 17

Figure S25. DEPT spectrum of compound **3** in $CDCl_3$. 18

Figure S26. HSQC spectrum of compound **3** in $CDCl_3$. 18

Figure S27. HMBC spectrum of compound **3** in $CDCl_3$. 19

Figure S28. ^{1}H-^{1}H COSY spectrum of compound **3** in $CDCl_3$. 19

Figure S29. NOESY spectrum of compound **3** in $CDCl_3$ 20

Figure S30. IR spectrum of compound **4**. 20

Figure S31. ESIMS spectrum of compound **4**. 21

Figure S32. HRESIMS spectrum of compound **4**. 21

Figure S33. ^{1}H NMR spectrum (400 MHz) of compound **4** in $CDCl_3$. 22

Figure S34. ^{13}C NMR spectrum (100 MHz) of compound **4** in $CDCl_3$. 22

Figure S35. HSQC spectrum of compound **4** in $CDCl_3$. 23

Figure S36. HMBC spectrum of compound **4** in $CDCl_3$. 23

Figure S37. ^{1}H-^{1}H COSY spectrum of compound **4** in $CDCl_3$. 24

Figure S38. NOESY spectrum of compound **4** in $CDCl_3$ 24

Figure S39. IR spectrum of compound **5**. 25

Figure S40. ESIMS spectrum of compound **5**. 25

Figure S41. HRESIMS spectrum of compound **5**. 26

Figure S42. ^{1}H NMR spectrum (600 MHz) of compound **5** in $CDCl_3$. 26

Figure S43. ^{13}C NMR spectrum (150 MHz) of compound **5** in $CDCl_3$. 27

Figure S44. HSQC spectrum of compound **5** in $CDCl_3$. 27

Figure S45. HMBC spectrum of compound **5** in $CDCl_3$. 28

Figure S46. ^{1}H-^{1}H COSY spectrum of compound **5** in $CDCl_3$. 28

Figure S47. NOESY spectrum of compound **5** in $CDCl_3$ 29

Figure S48. IR spectrum of compound **6.** 29

Figure S49. ESIMS spectrum of compound **6**. 30

Figure S50. HRESIMS spectrum of compound **6**. 30

Figure S51. ^{1}H NMR spectrum (600 MHz) of compound **6** in $CDCl_3$. 31

Figure S52. ^{13}C NMR spectrum (150 MHz) of compound **6** in $CDCl_3$. 31

Figure S53. HSQC spectrum of compound **6** in $CDCl_3$. 32

Figure S54. HMBC spectrum of compound **6** in $CDCl_3$. 32

Figure S55. ^{1}H-^{1}H COSY spectrum of compound **6** in $CDCl_3$. 33

Figure S56. NOESY spectrum of compound **6** in $CDCl_3$ 33

Figure S57. IR spectrum of compound **7.** 34

Figure S58. ESIMS spectrum of compound **7**. 34

Figure S59. HRESIMS spectrum of compound **7**. 35

Figure S60. 1H NMR spectrum (400 MHz) of compound **7** in CDCl3. 35

Figure S61. ^{13}C NMR spectrum (100 MHz) of compound **7** in $CDCl_3$. 36

Figure S62. DEPT spectrum of compound **7** in $CDCl_3$.......... 36

Figure S63. HSQC spectrum of compound **7** in $CDCl_3$.......... 37

Figure S64. HMBC spectrum of compound **7** in $CDCl_3$.......... 37

Figure S65. ^1H-^1H COSY spectrum of compound **7** in $CDCl_3$. 38

Figure S66. NOESY spectrum of compound **7** in $CDCl_3$ 38

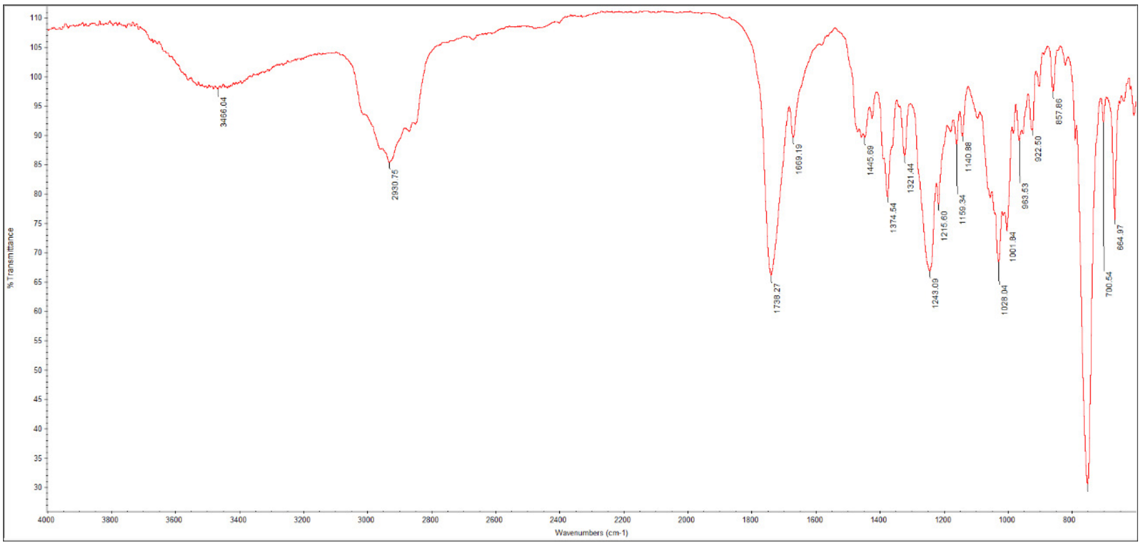

Figure S1. IR spectrum of compound **1**.

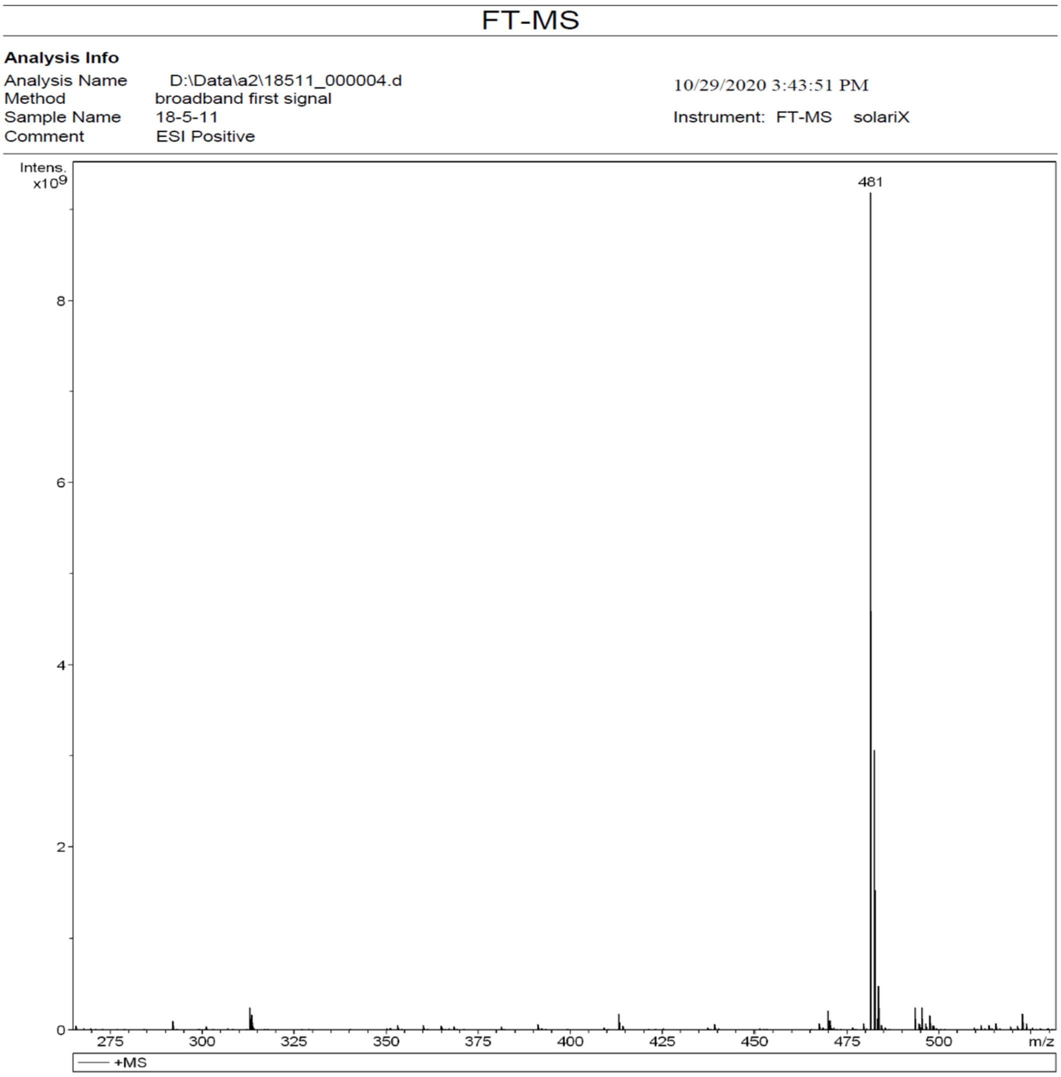

Figure S2. ESIMS spectrum of compound **1**.

Mass Spectrum SmartFormula Report

Analysis Info

Analysis Name	D:\Data\a2\18511_000003.d	10/29/2020 3:42:39 PM
Method	broadband first signal	Operator: YU HSIAO-CHING
Sample Name	18-5-11	Instrument: BRUKER FT-MS solariX
Comment	ESI Positive	

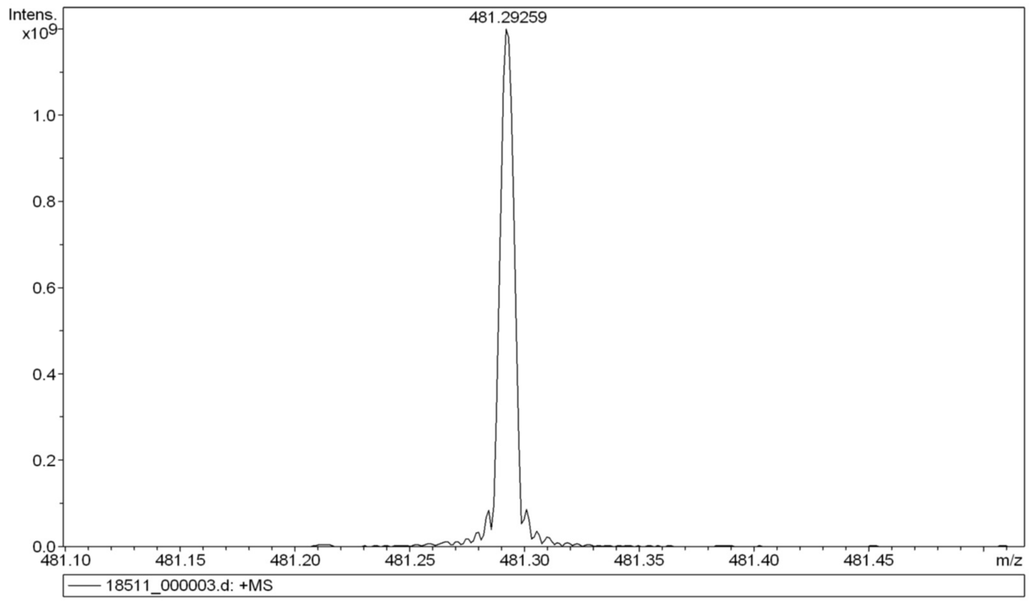

Meas. m/z	#	Formula	Score	m/z	err [mDa]	err [ppm]	mSigma	rdb	e¯ Conf	N-Rule
481.29259	1	C 28 H 42 Na O 5	100.00	481.29245	-0.14	-0.30	21.5	7.5	even	ok

Figure S3. HRESIMS spectrum of compound **1**.

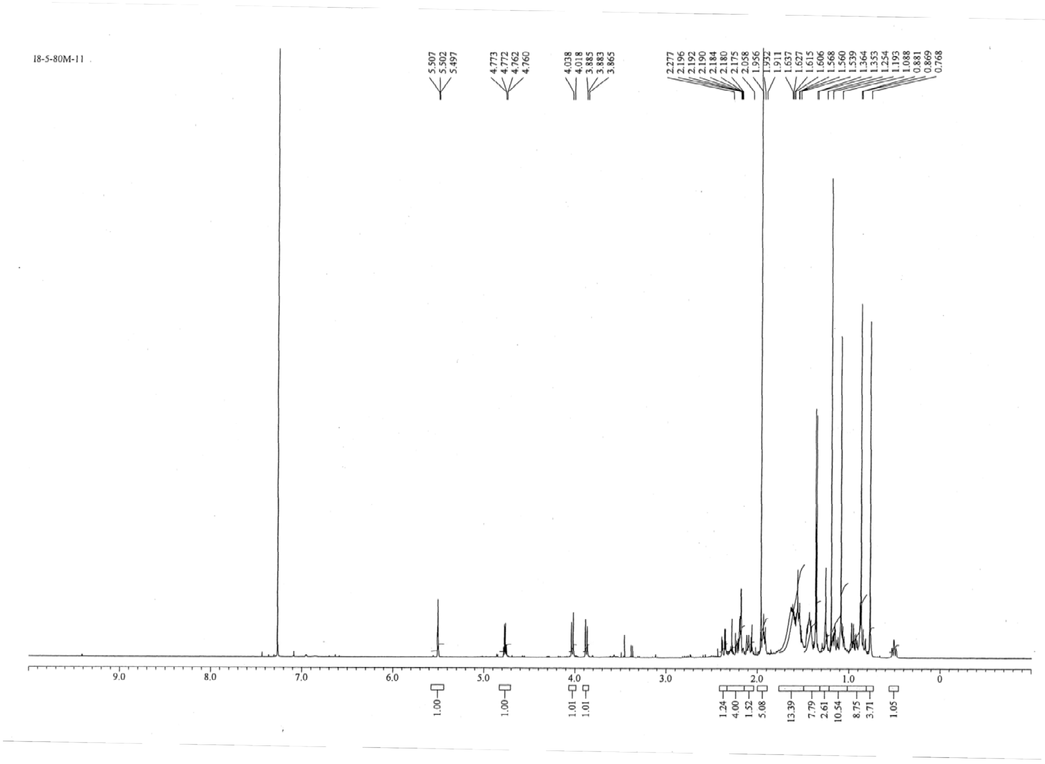

Figure S4. ^{1}H NMR spectrum (600 MHz) of compound **1** in $CDCl_3$.

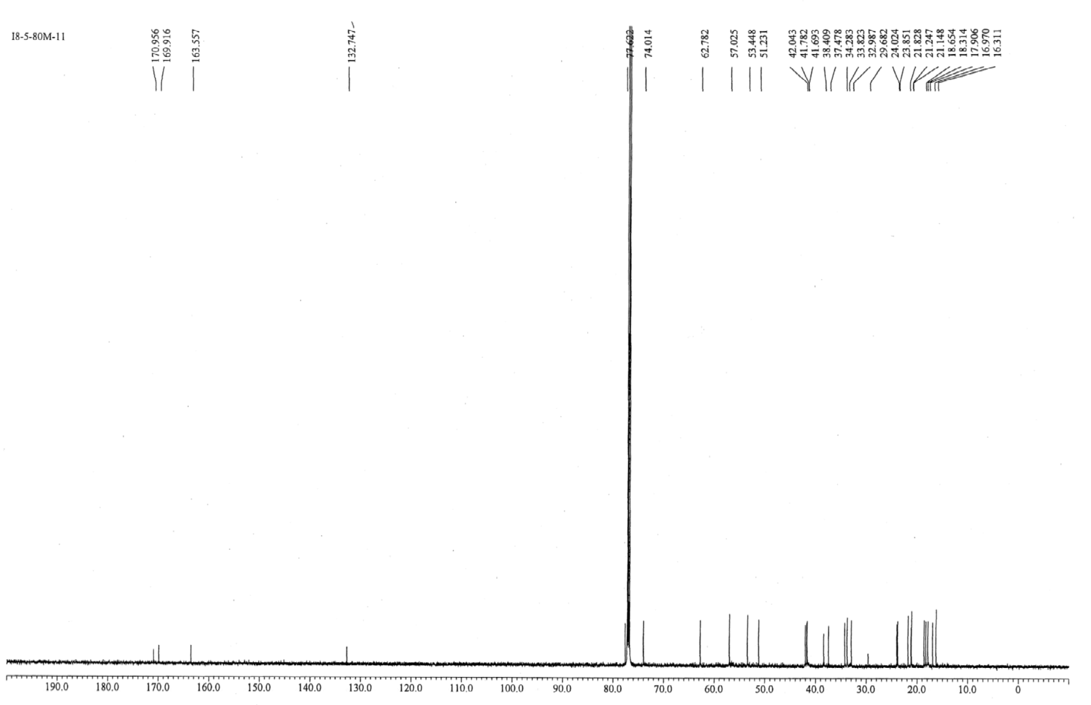

Figure S5. ^{13}C NMR spectrum (150 MHz) of compound **1** in $CDCl_3$.

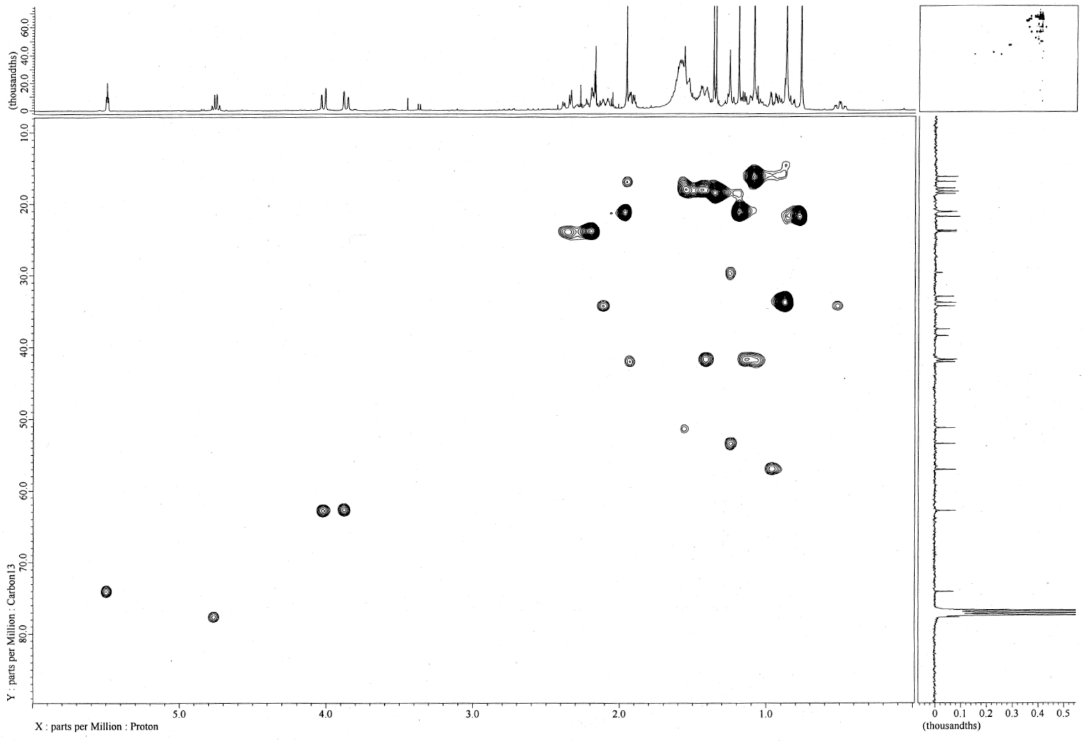

Figure S6. HSQC spectrum of compound **1** in $CDCl_3$.

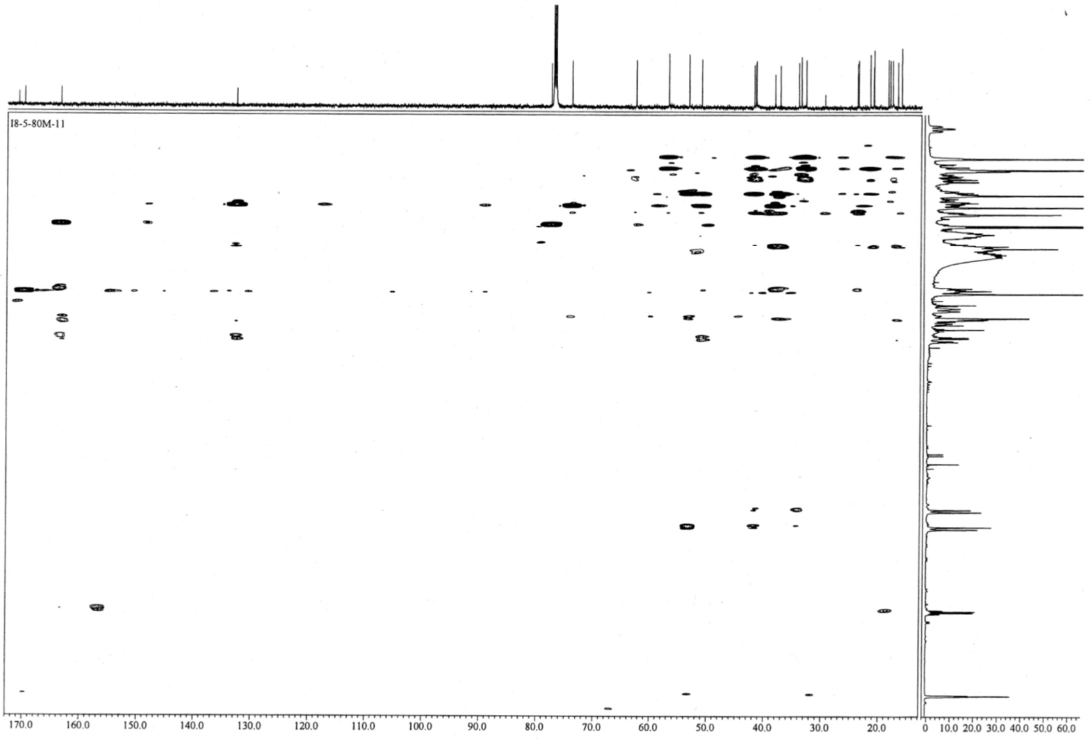

Figure S7. HMBC spectrum of compound **1** in $CDCl_3$.

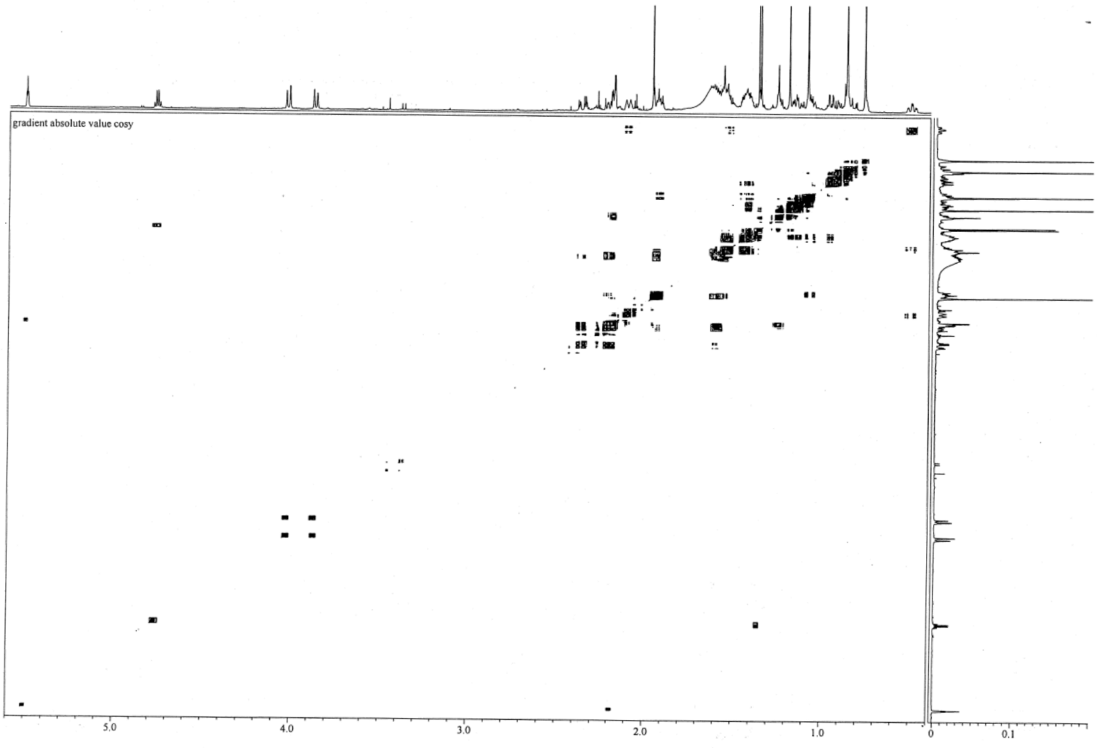

Figure S8. ^1H-^1H COSY spectrum of compound **1** in $CDCl_3$.

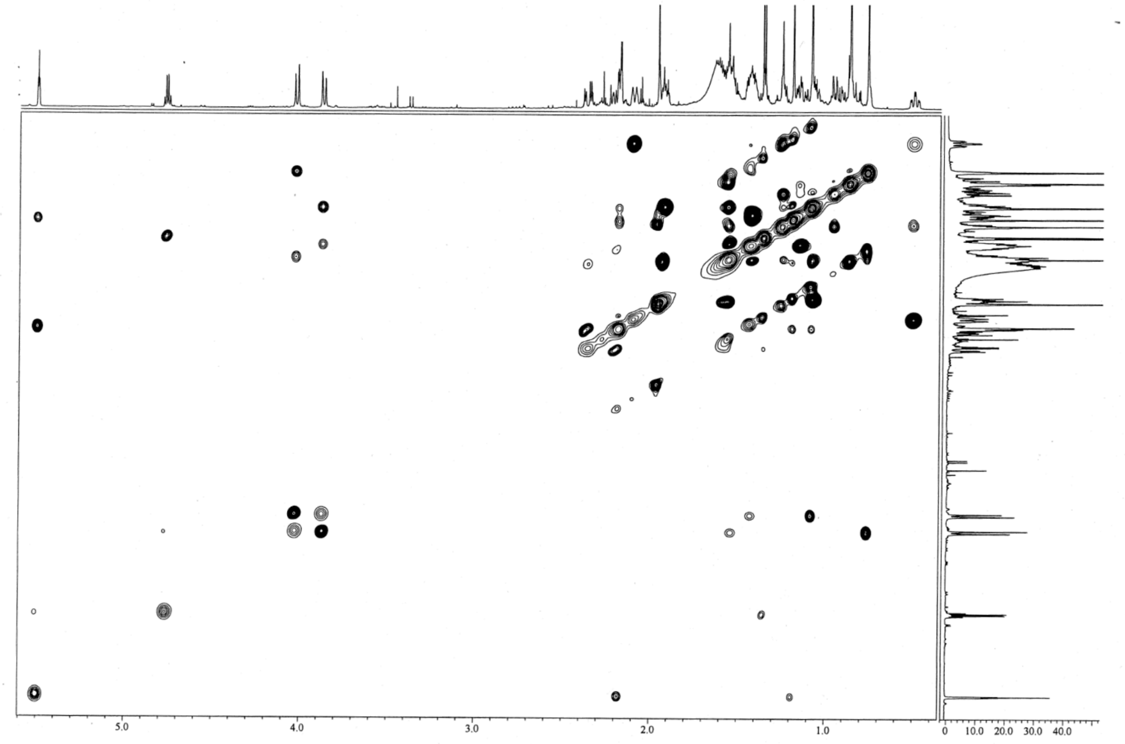

Figure S9. NOESY spectrum of compound **1** in $CDCl_3$

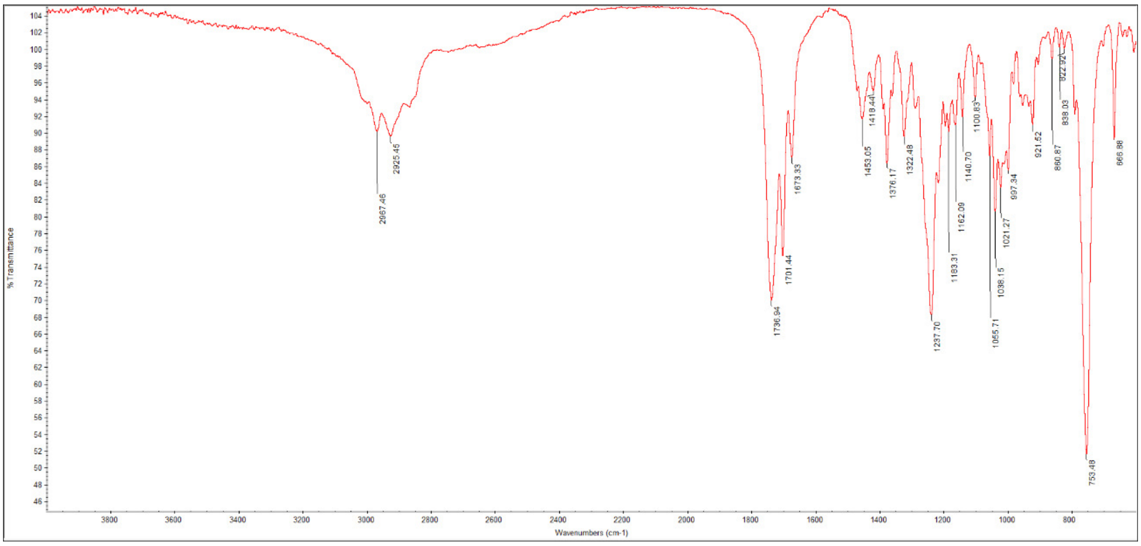

Figure S10. IR spectrum of compound **2**.

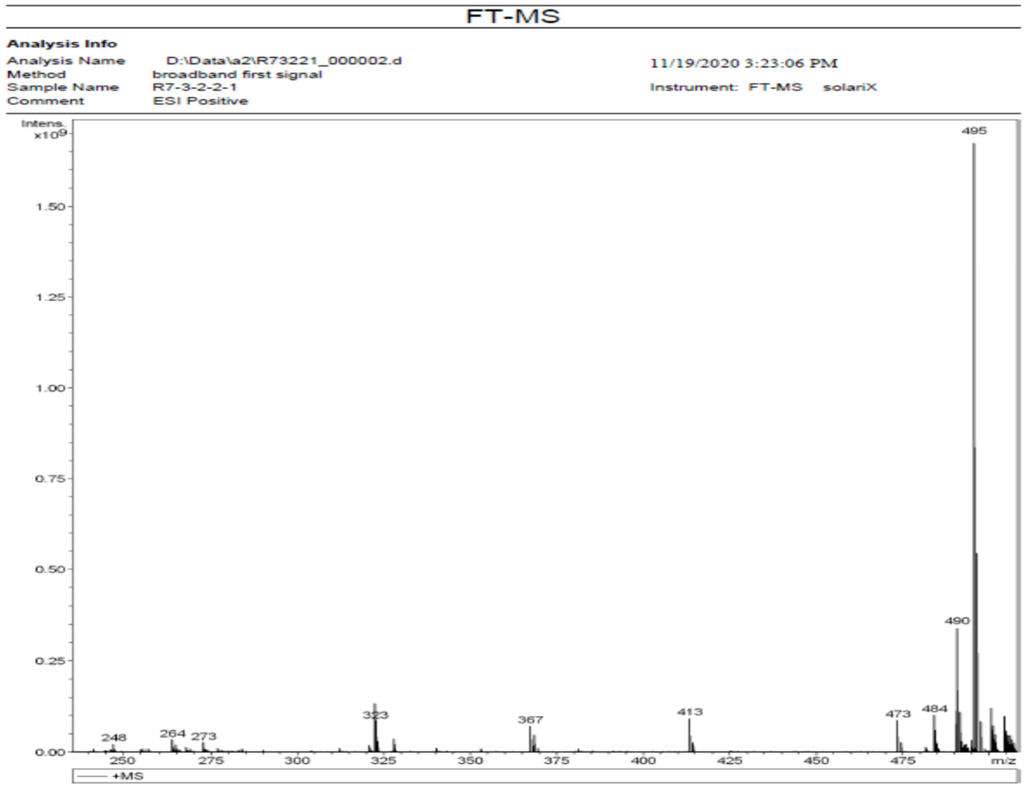

Figure S11. ESIMS spectrum of compound **2**.

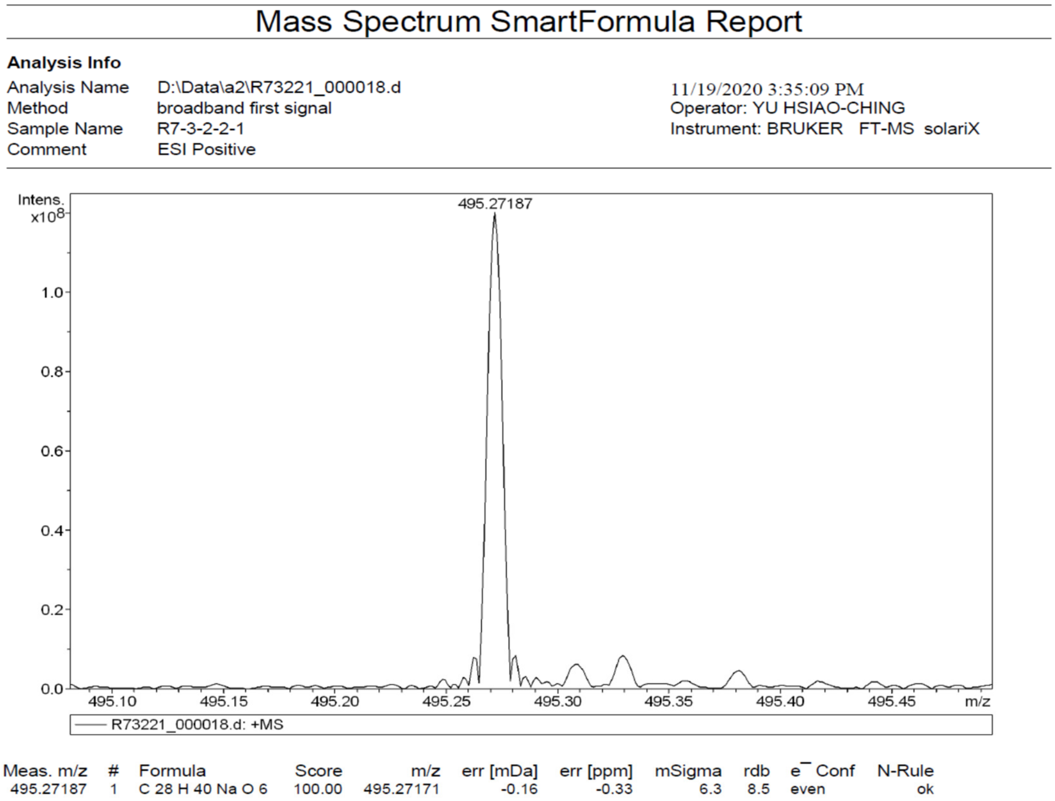

Figure S12. HRESIMS spectrum of compound **2**.

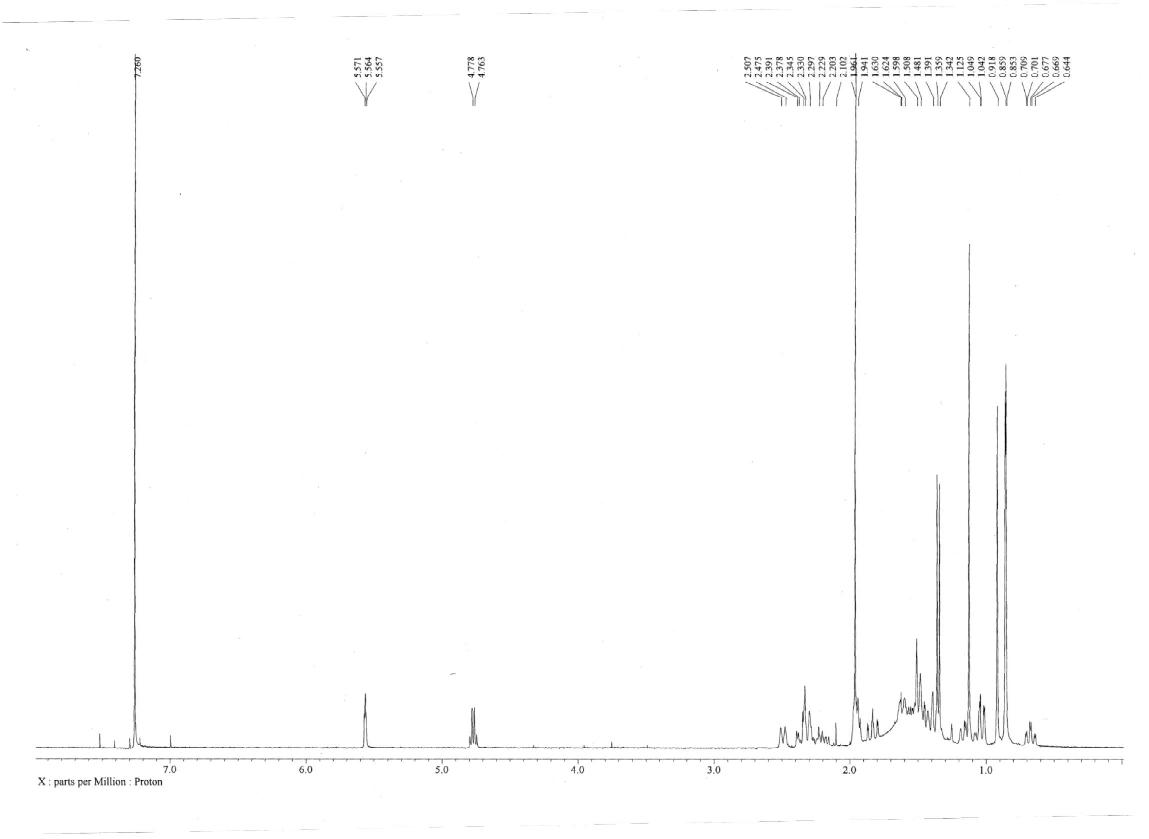

Figure S13. ^{1}H NMR spectrum (400 MHz) of compound **2** in $CDCl_3$.

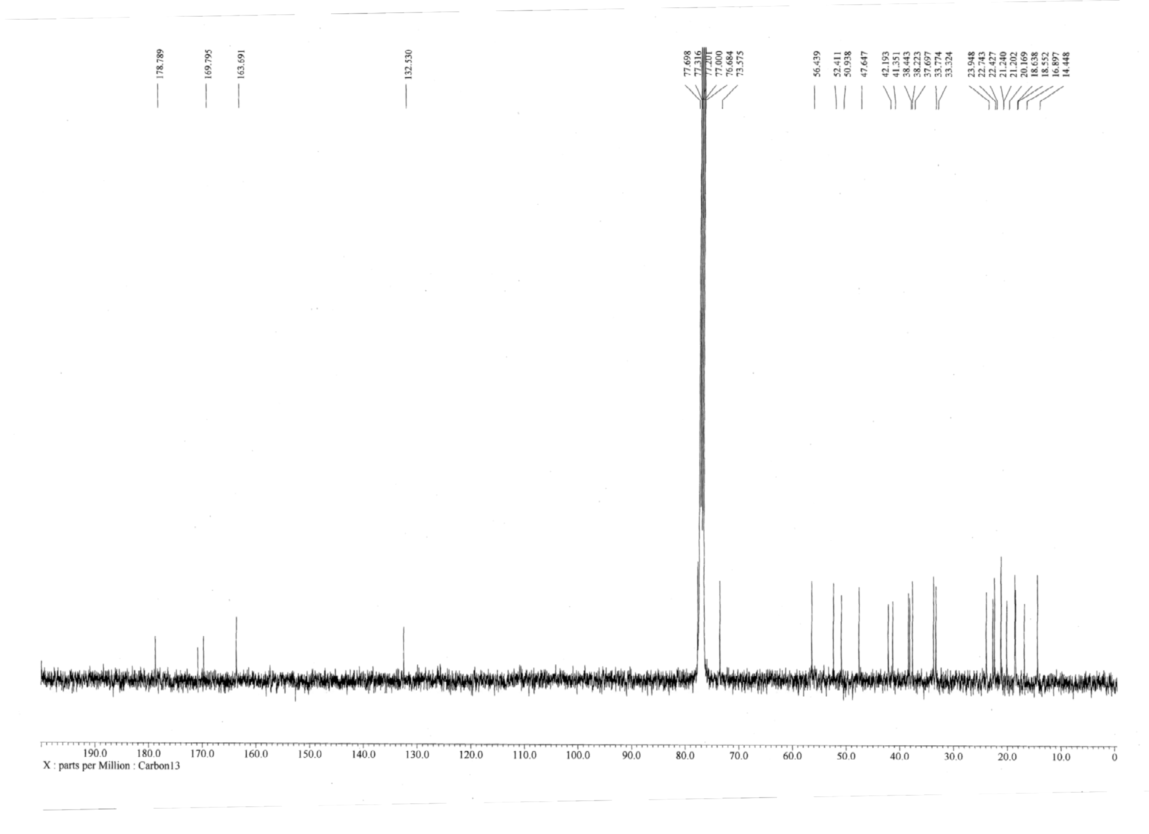

Figure S14. ^{13}C NMR spectrum (100 MHz) of compound **2** in $CDCl_3$.

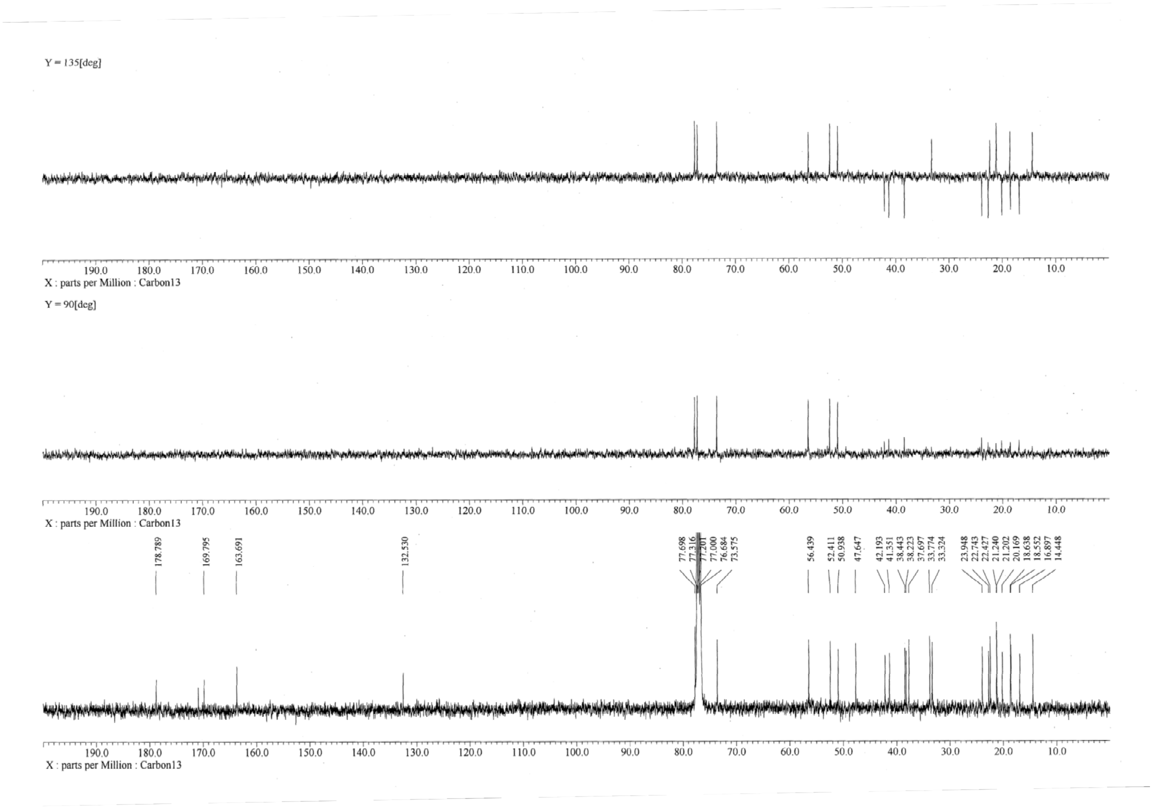

Figure S15. DEPT spectrum of compound **2** in $CDCl_3$.

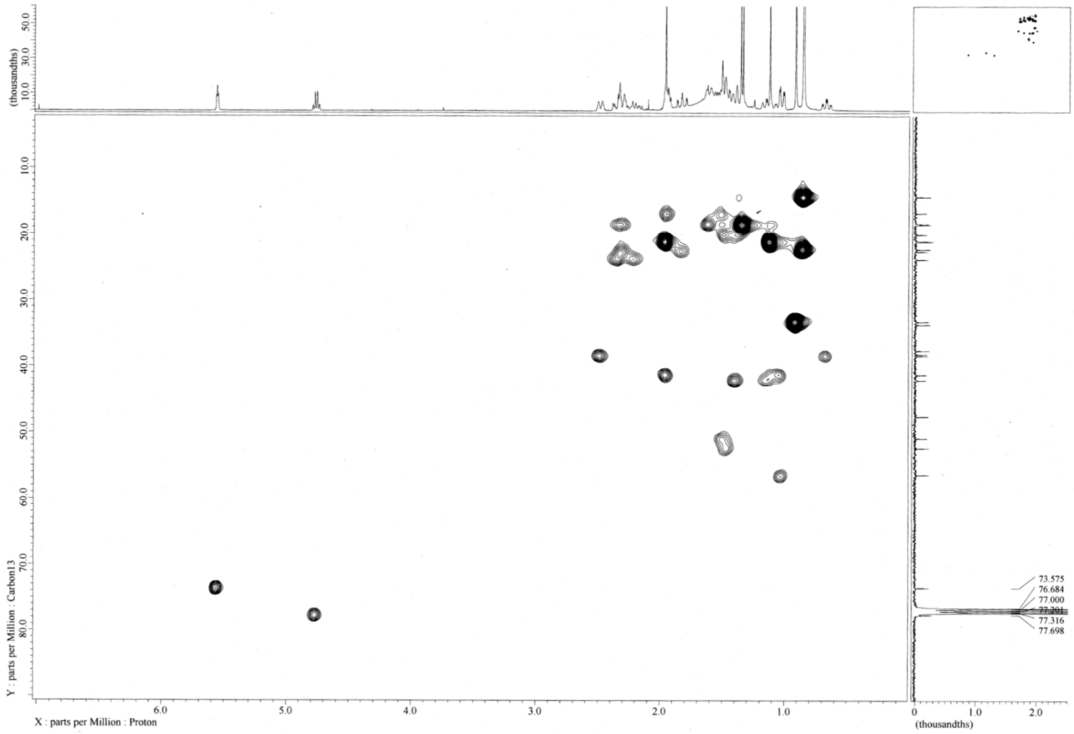

Figure S16. HSQC spectrum of compound **2** in $CDCl_3$.

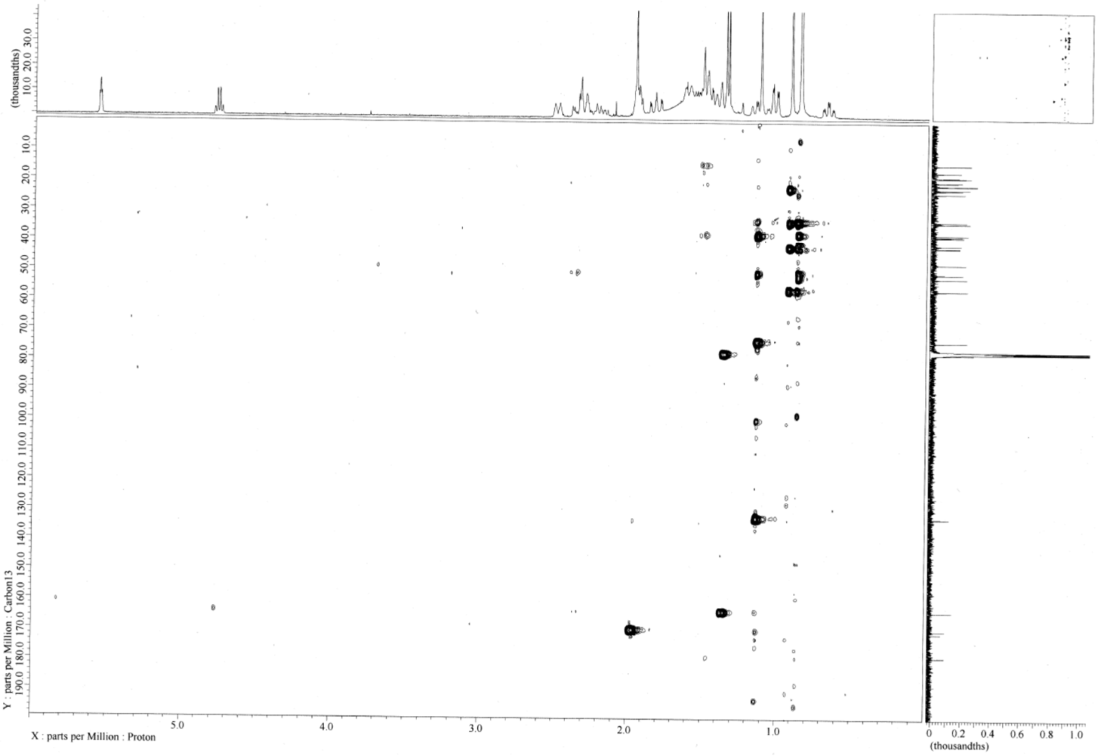

Figure S17. HMBC spectrum of compound **2** in $CDCl_3$.

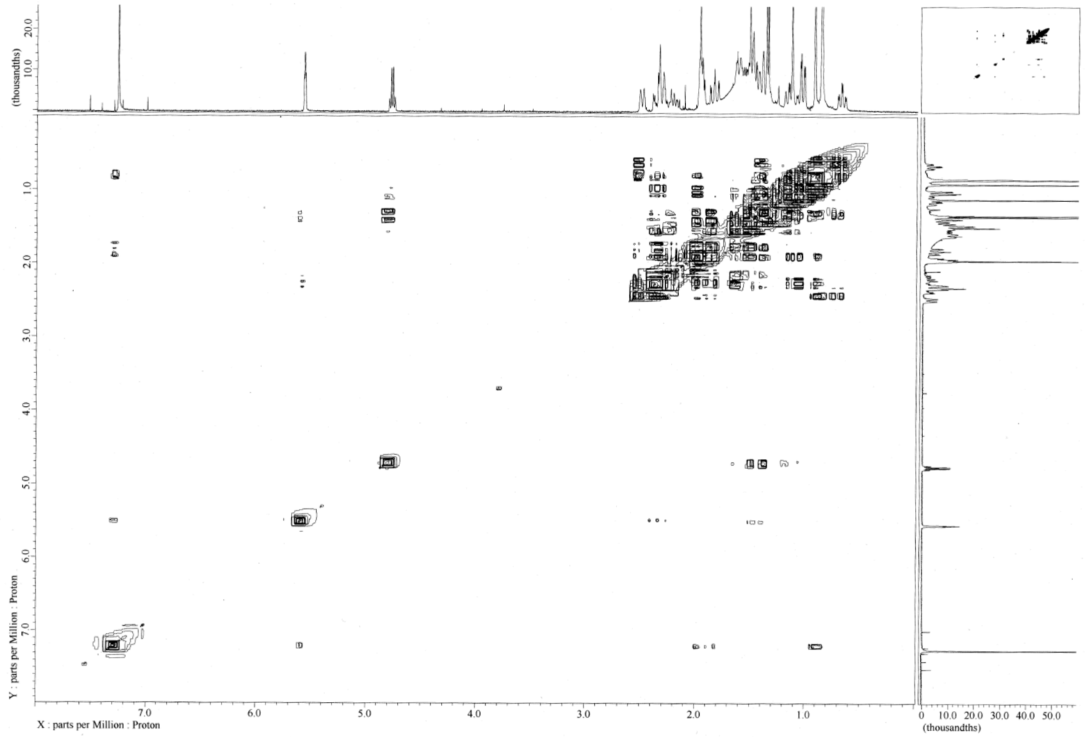

Figure S18. ^1H-^1H COSY spectrum of compound **2** in $CDCl_3$.

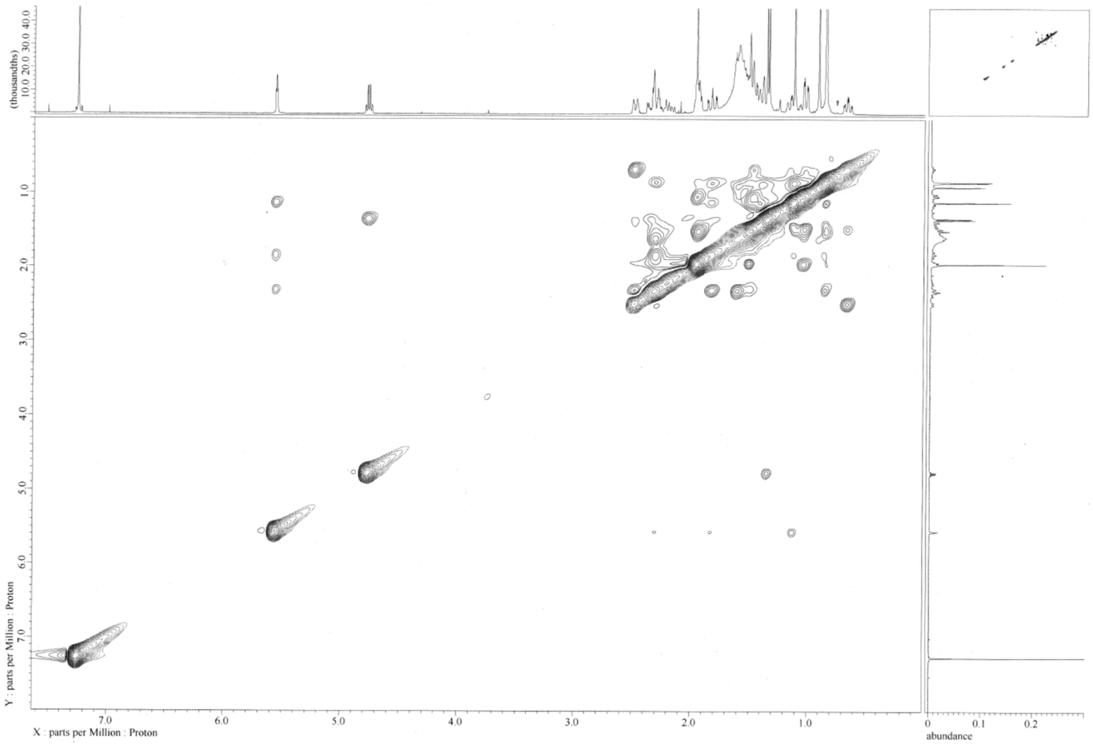

Figure S19. NOESY spectrum of compound **2** in $CDCl_3$

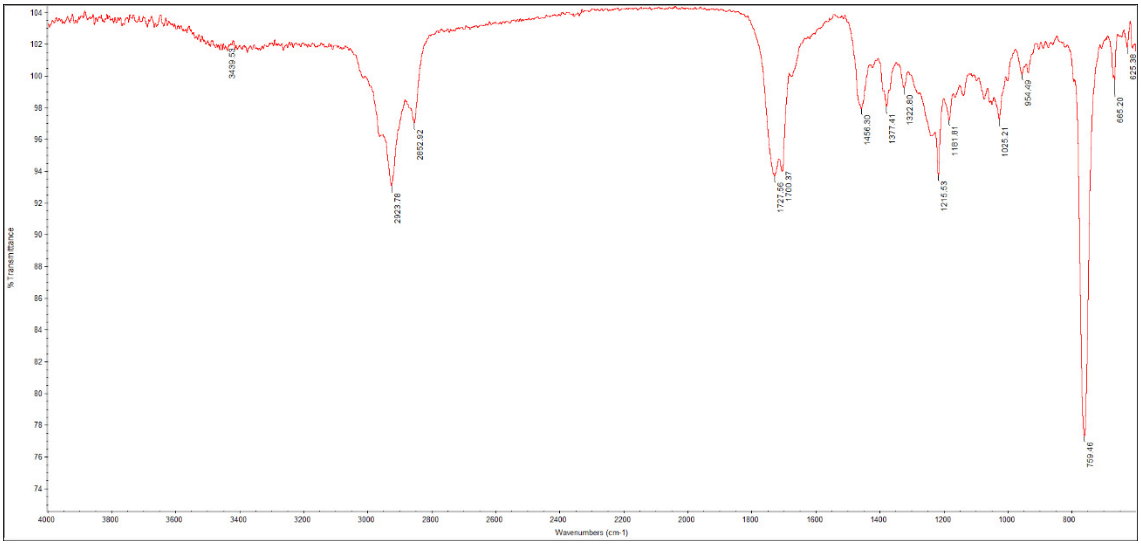

Figure S20. IR spectrum of compound **3**.

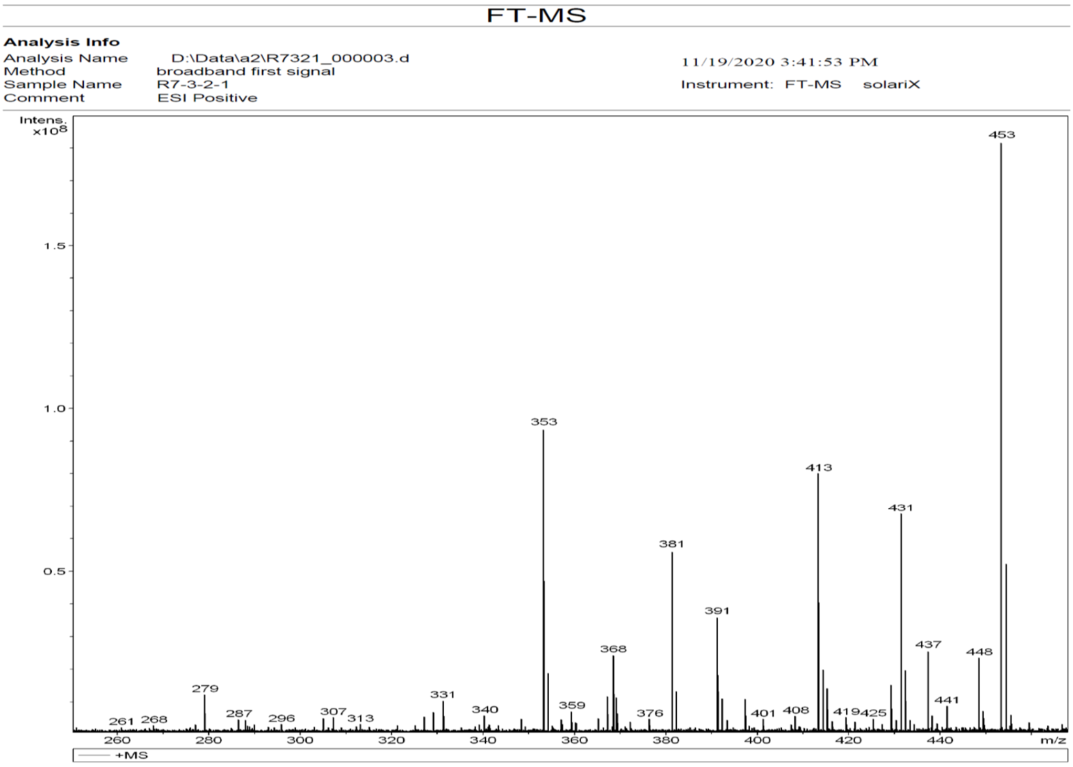

Figure S21. ESIMS spectrum of compound **3**.

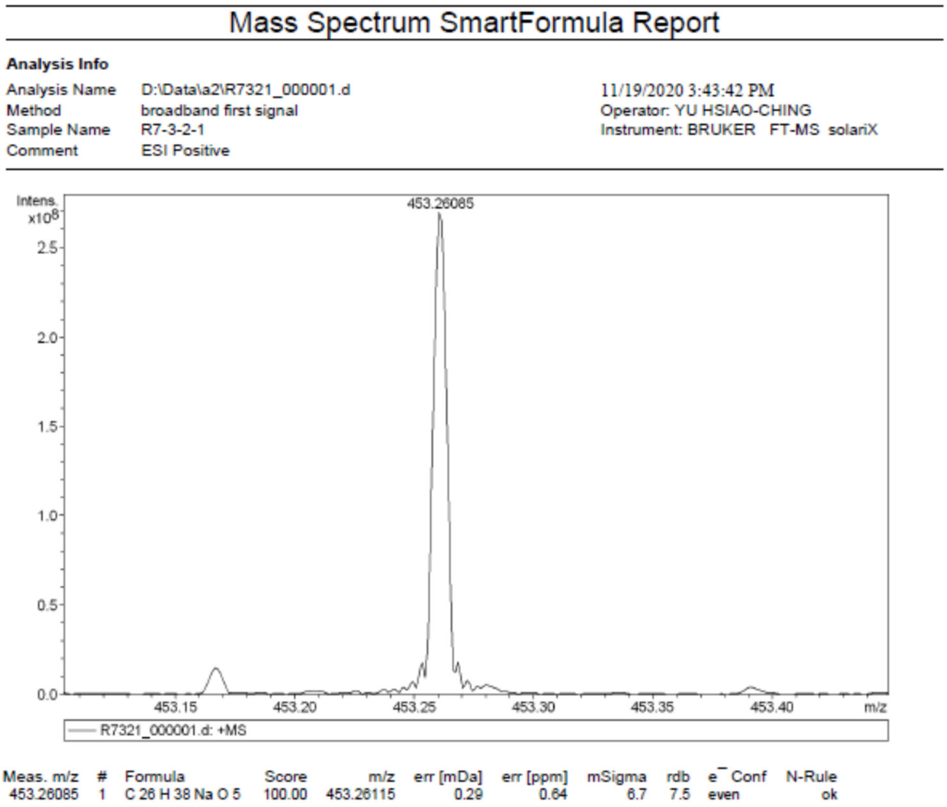

Figure S22. HRESIMS spectrum of compound **3**.

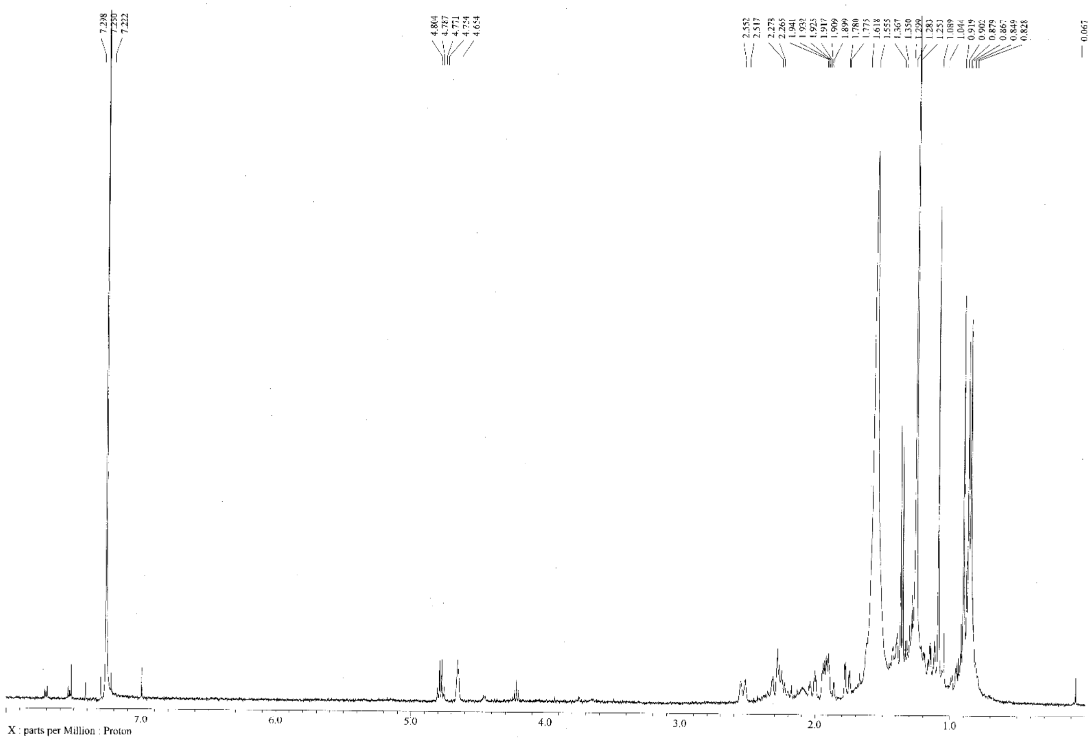

Figure S23. ^{1}H NMR spectrum (400 MHz) of compound **3** in $CDCl_3$.

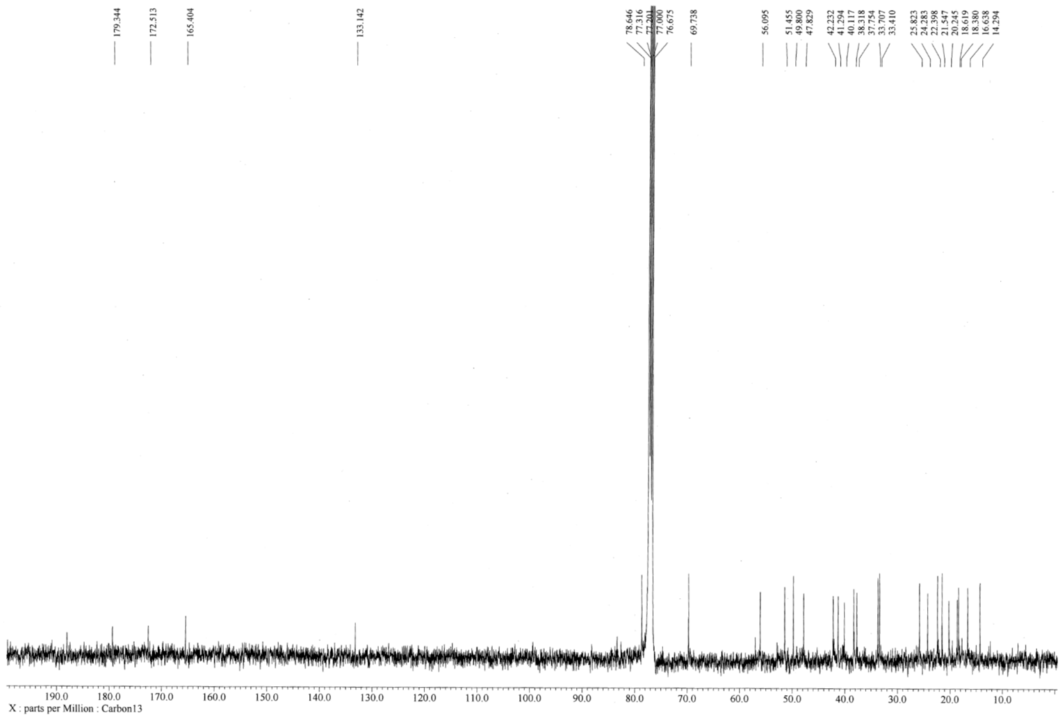

Figure S24. ^{13}C NMR spectrum (100 MHz) of compound **3** in $CDCl_3$.

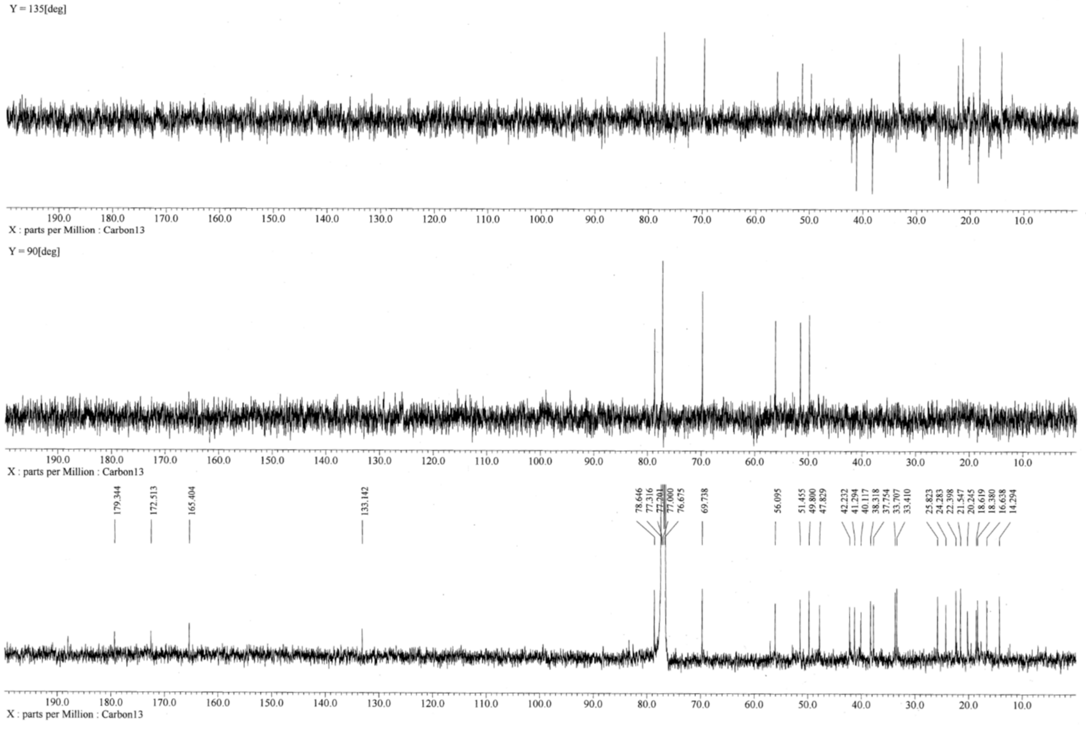

Figure S25. DEPT spectrum of compound **3** in $CDCl_3$.

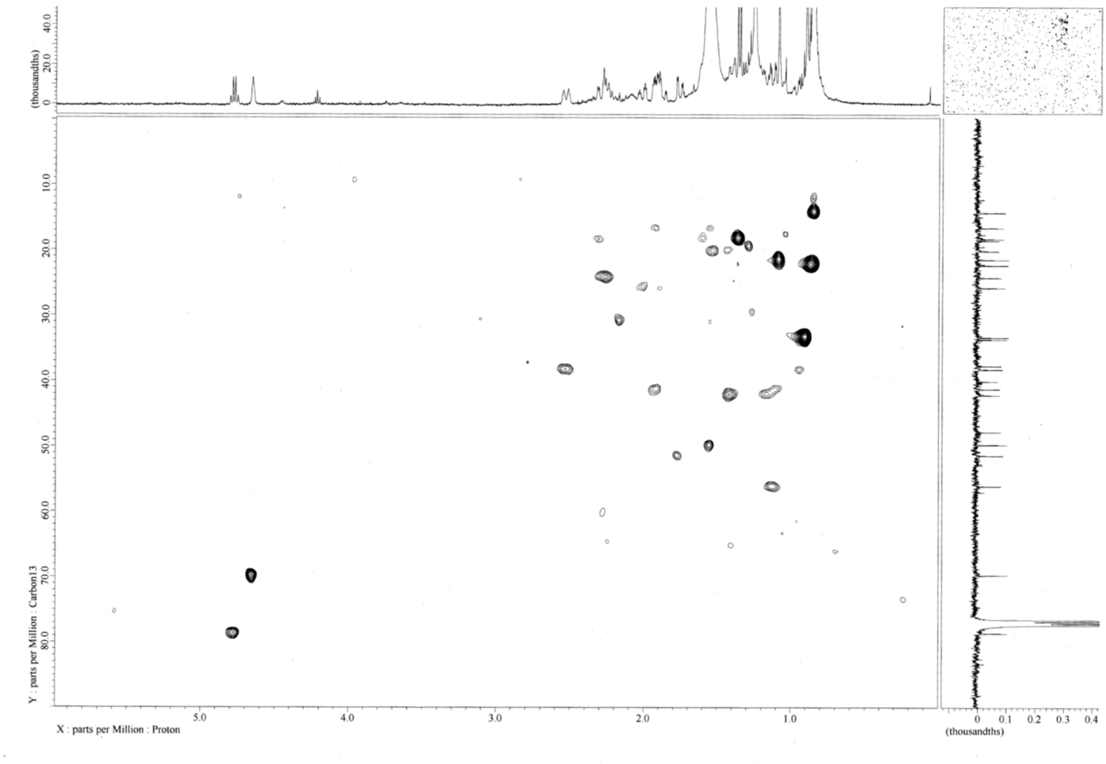

Figure S26. HSQC spectrum of compound **3** in $CDCl_3$.

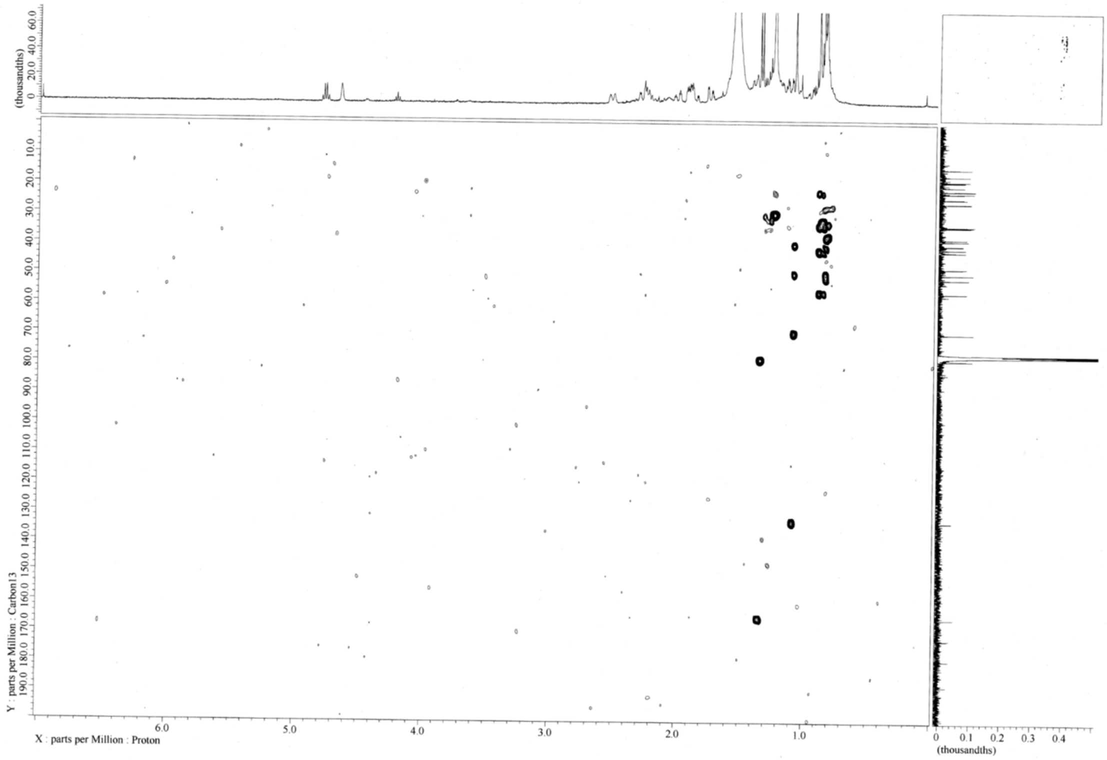

Figure S27. HMBC spectrum of compound **3** in $CDCl_3$.

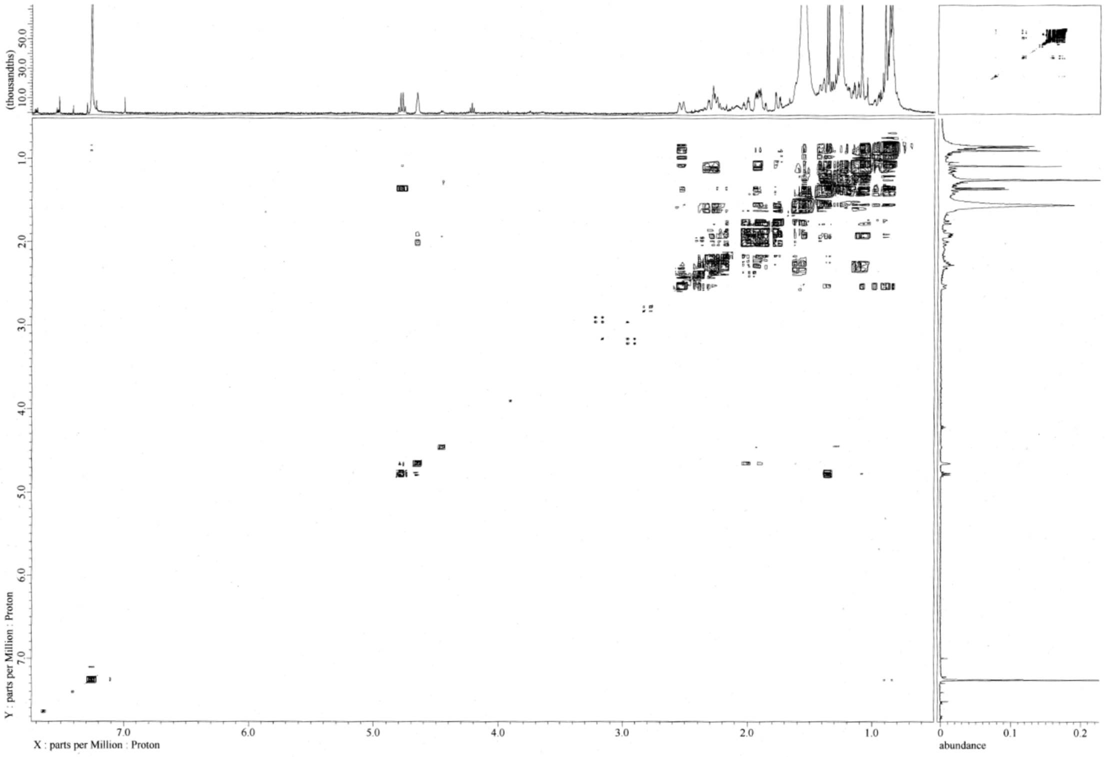

Figure S28. ^1H-^1H COSY spectrum of compound **3** in $CDCl_3$.

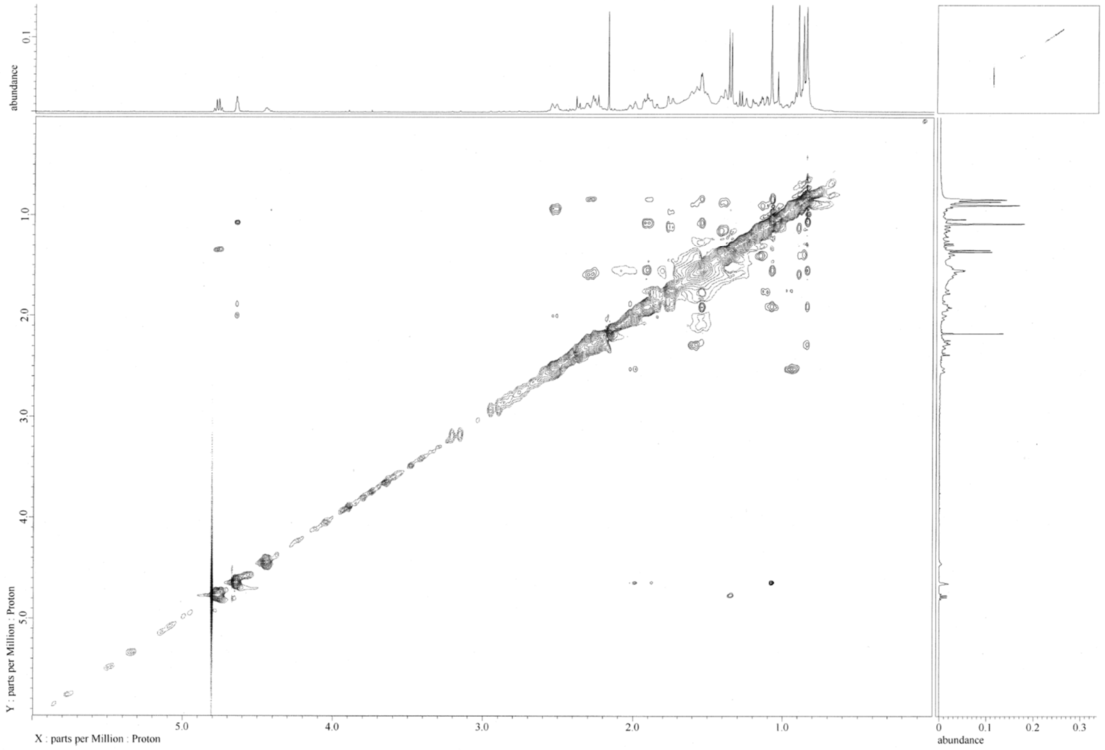

Figure S29. NOESY spectrum of compound **3** in $CDCl_3$

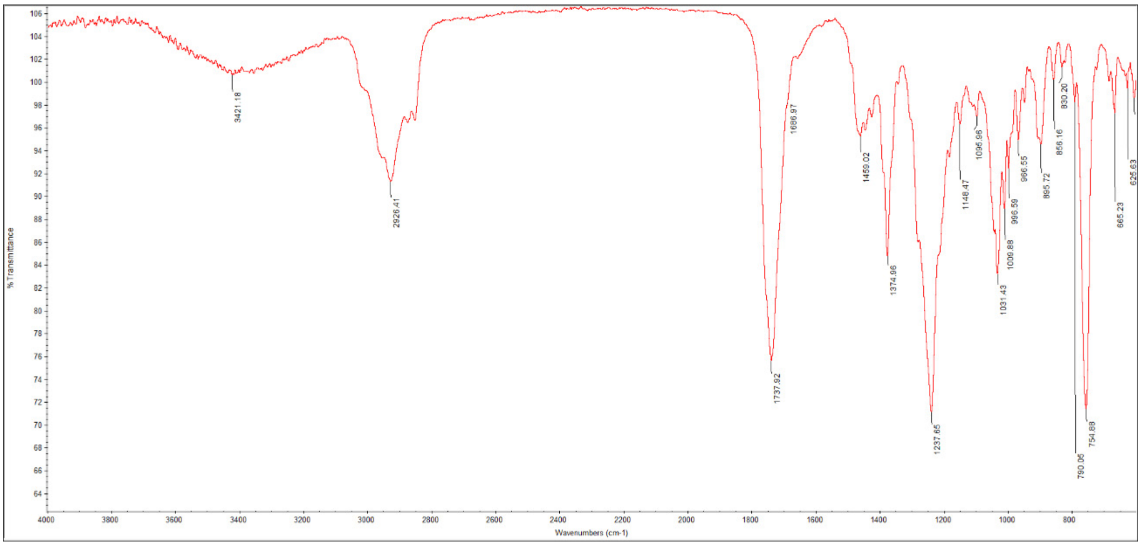

Figure S30. IR spectrum of compound **4**.

FT-MS

Analysis Info

Analysis Name	D:\Data\a2\185132_000001.d	10/29/2020 4:05:54 PM
Method	broadband first signal	Instrument: FT-MS solariX
Sample Name	18-5-13-2	
Comment	ESI Positive	

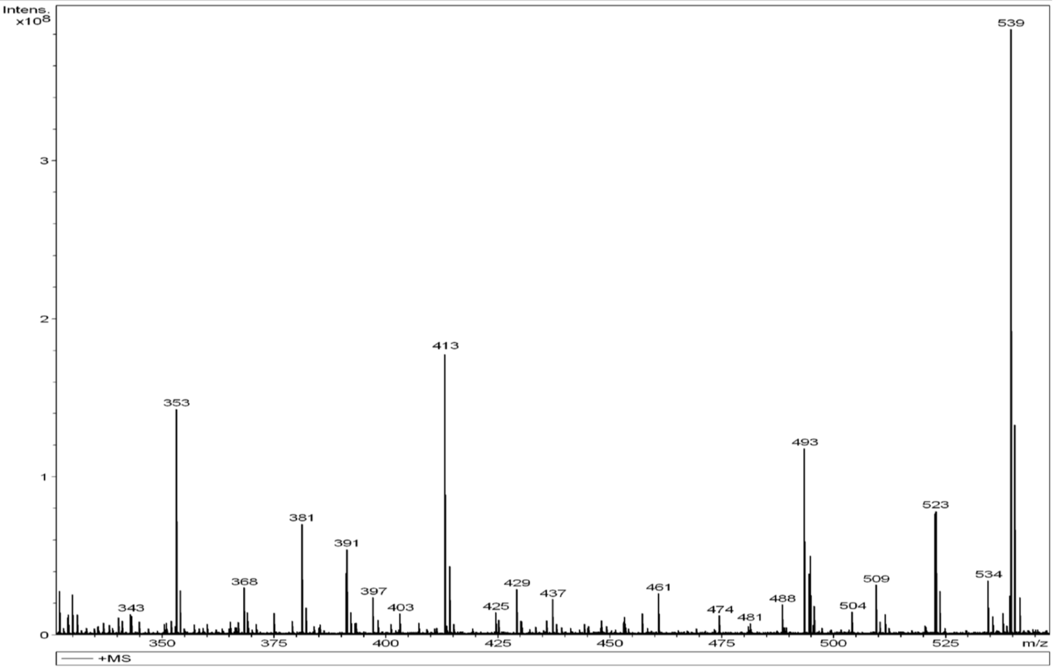

Figure S31. ESIMS spectrum of compound **4**.

Mass Spectrum SmartFormula Report

Analysis Info

Analysis Name	D:\Data\a2\185132_000002.d	10/29/2020 4:05:08 PM
Method	broadband first signal	Operator: YU HSIAO-CHING
Sample Name	18-5-13-2	Instrument: BRUKER FT-MS solariX
Comment	ESI Positive	

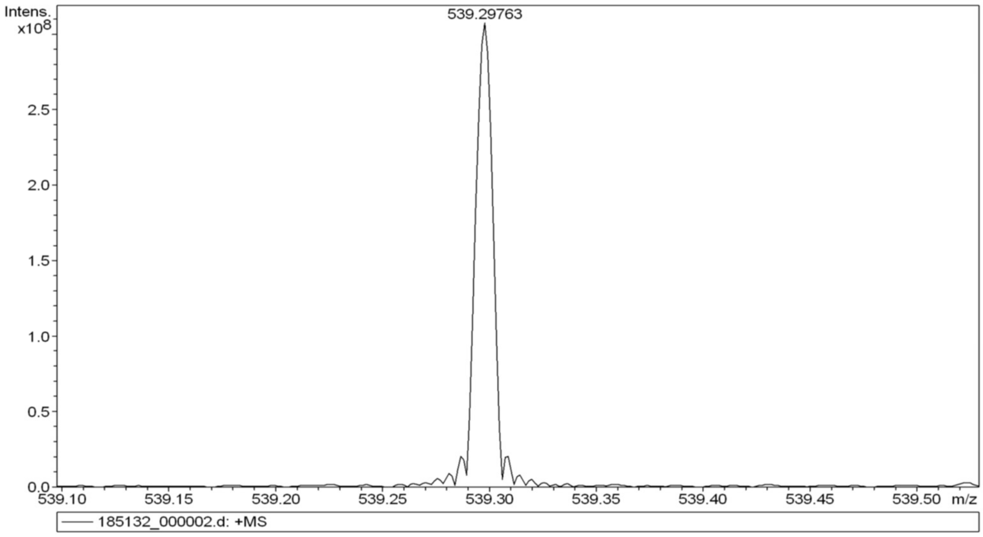

Meas. m/z	#	Formula	Score	m/z	err [mDa]	err [ppm]	mSigma	rdb	e⁻ Conf	N-Rule
539.29763	1	C 30 H 44 Na O 7	100.00	539.29792	0.30	0.55	22.2	8.5	even	ok

Figure S32. HRESIMS spectrum of compound **4**.

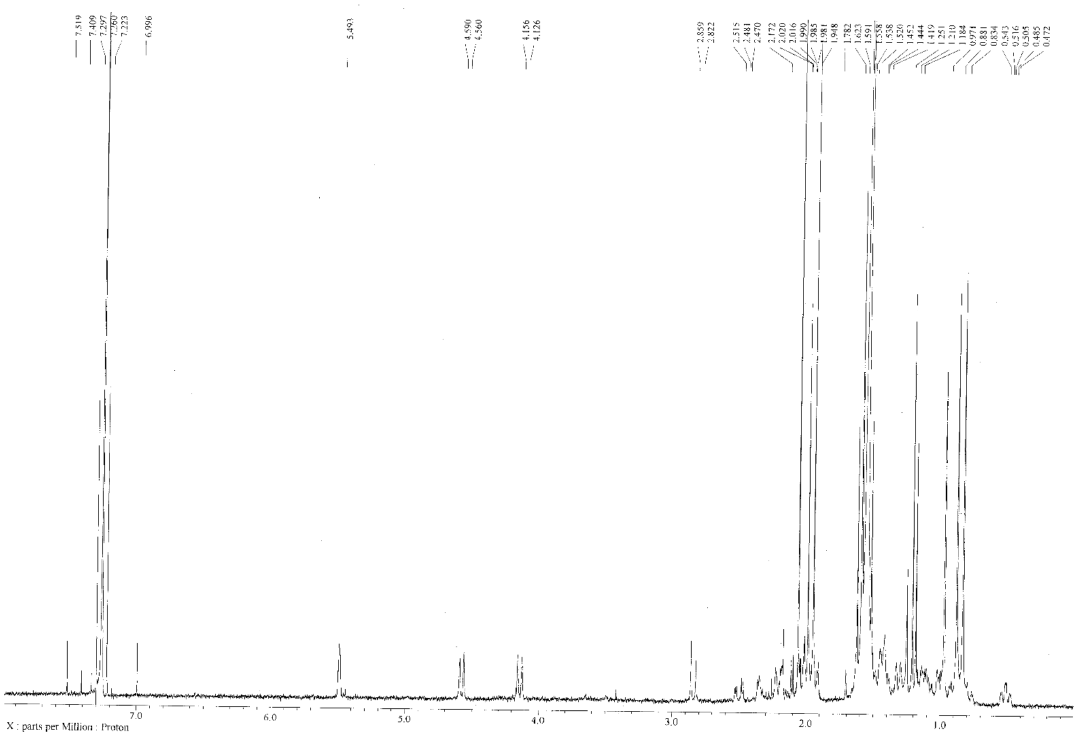

Figure S33. ^{1}H NMR spectrum (400 MHz) of compound **4** in $CDCl_3$.

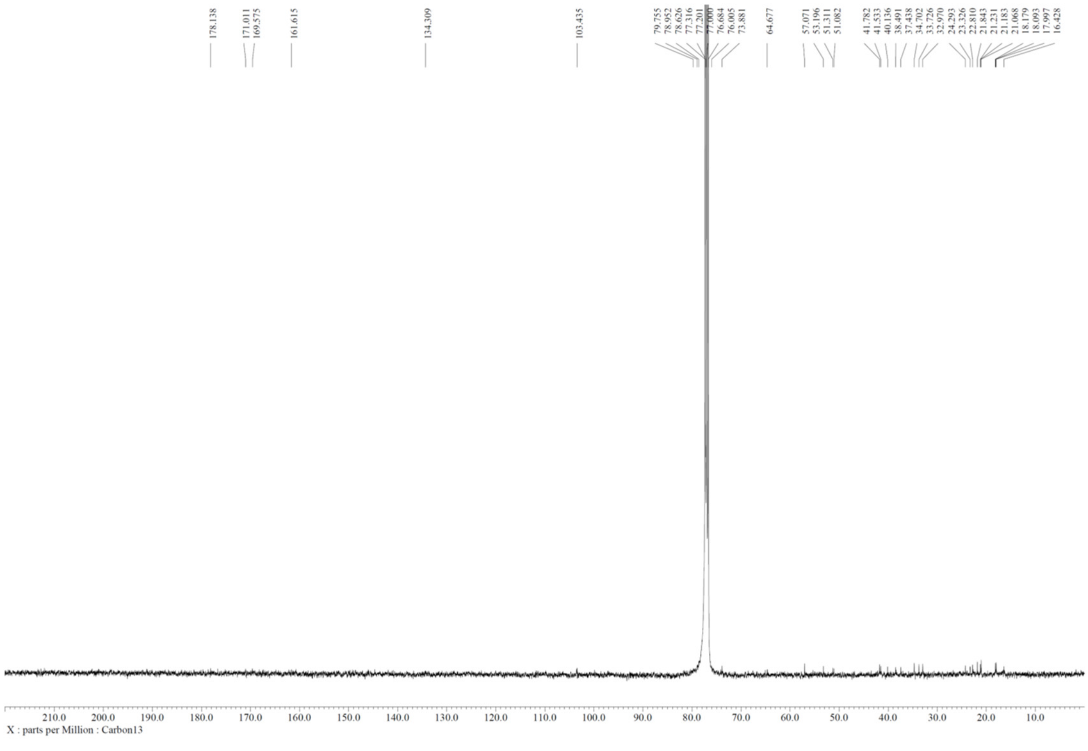

Figure S34. ^{13}C NMR spectrum (100 MHz) of compound **4** in $CDCl_3$.

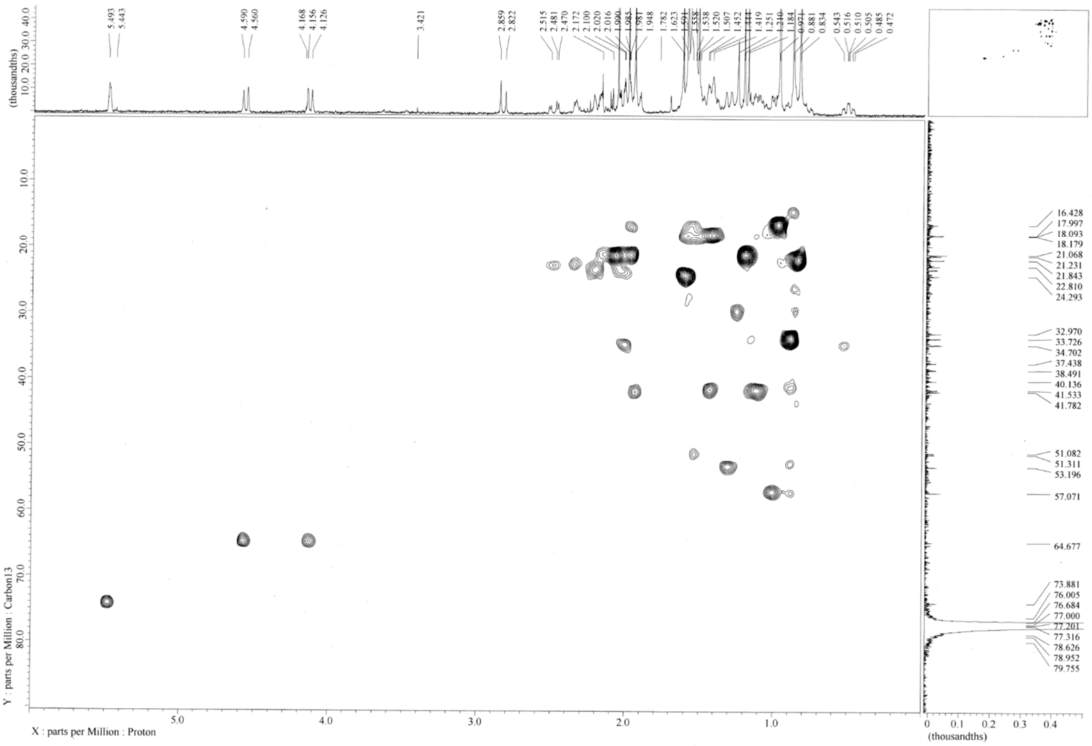

Figure S35. HSQC spectrum of compound **4** in $CDCl_3$.

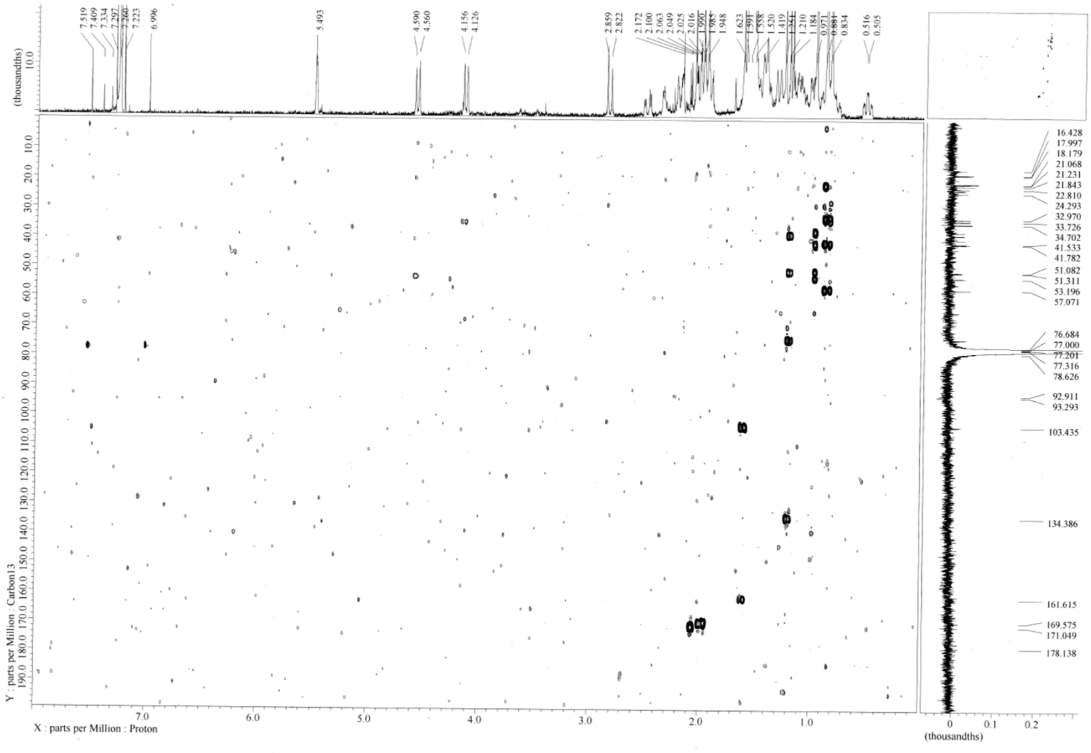

Figure S36. HMBC spectrum of compound **4** in $CDCl_3$.

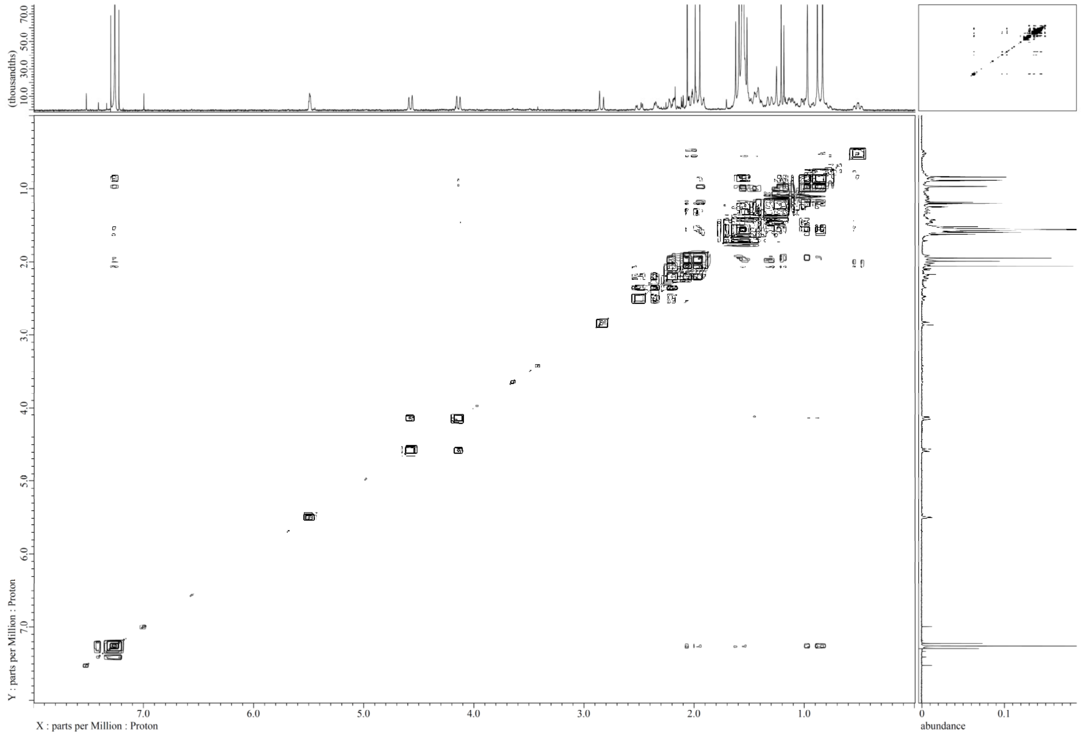

Figure S37. ^{1}H-^{1}H COSY spectrum of compound **4** in $CDCl_3$.

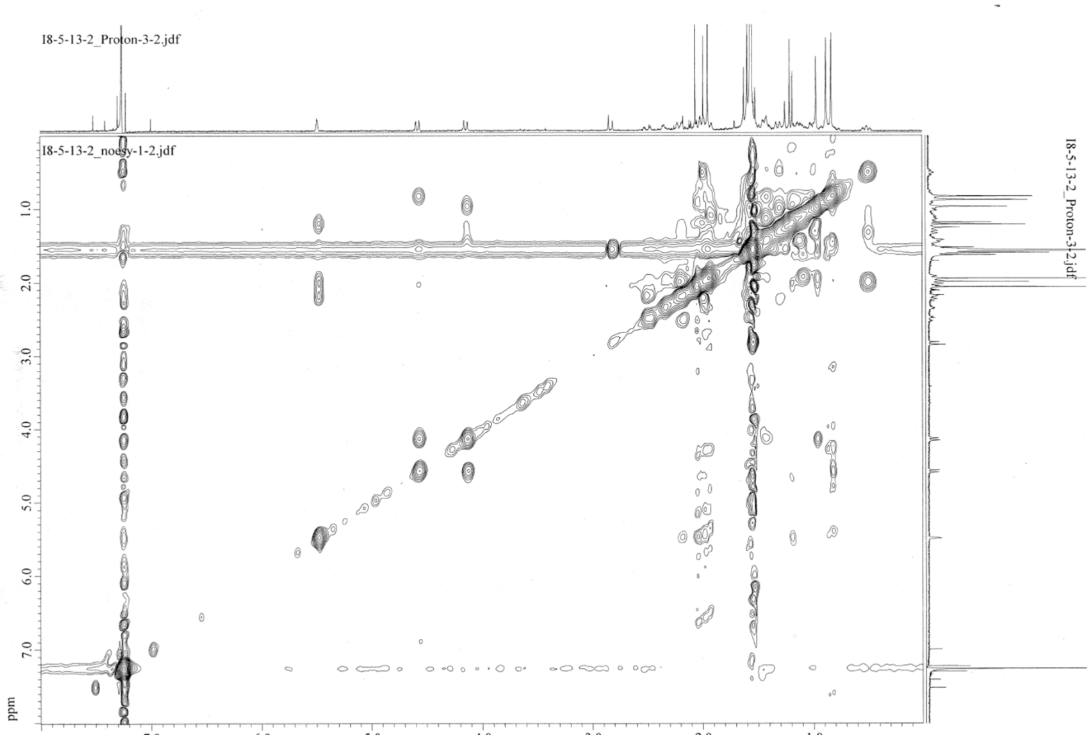

Figure S38. NOESY spectrum of compound **4** in $CDCl_3$

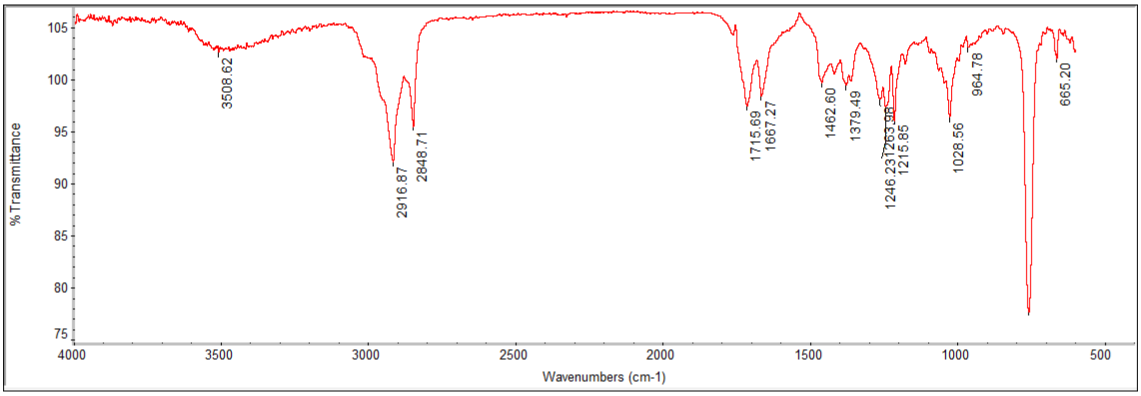

Figure S39. IR spectrum of compound **5.**

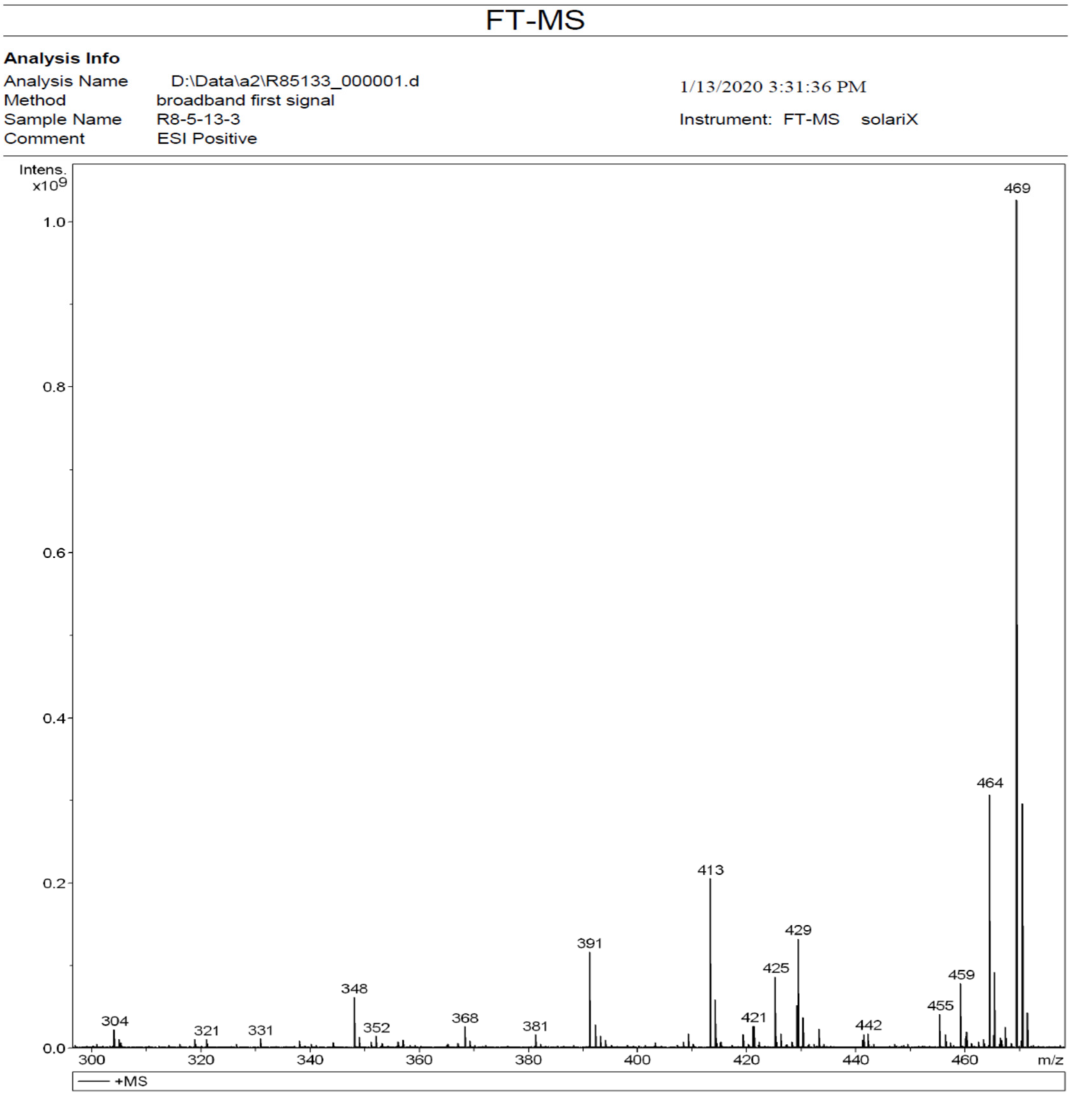

Figure S40. ESIMS spectrum of compound **5**.

Mass Spectrum SmartFormula Report

Analysis Info

Analysis Name	D:\Data\a2\R85133_000002.d	1/13/2020 3:30:43 PM
Method	broadband first signal	Operator: YU HSIAO-CHING
Sample Name	R8-5-13-3	Instrument: BRUKER FT-MS solariX
Comment	ESI Positive	

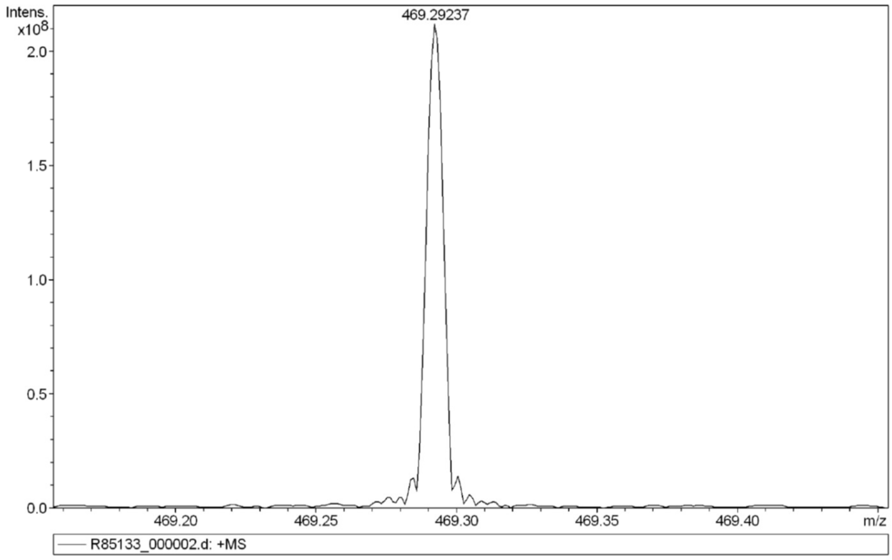

Meas. m/z	#	Formula	Score	m/z	err [mDa]	err [ppm]	mSigma	rdb	e⁻ Conf	N-Rule
469.29237	1	C 27 H 42 Na O 5	100.00	469.29245	0.07	0.15	7.2	6.5	even	ok

Figure S41. HRESIMS spectrum of compound **5**.

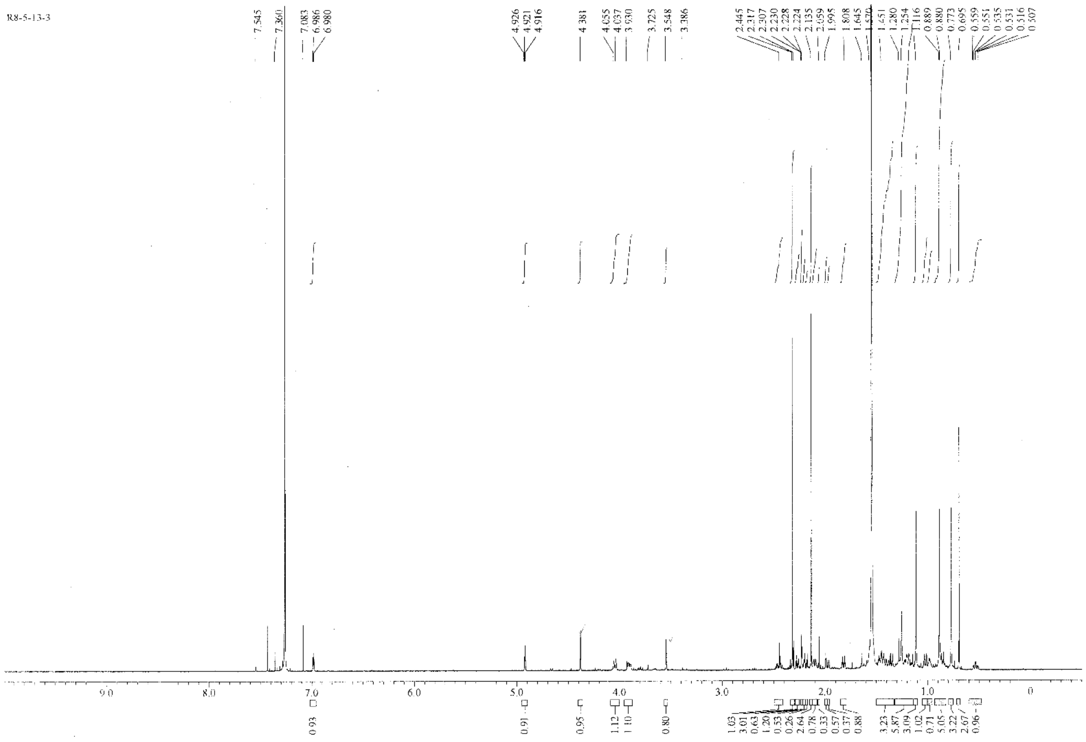

Figure S42. ^{1}H NMR spectrum (600 MHz) of compound **5** in $CDCl_3$.

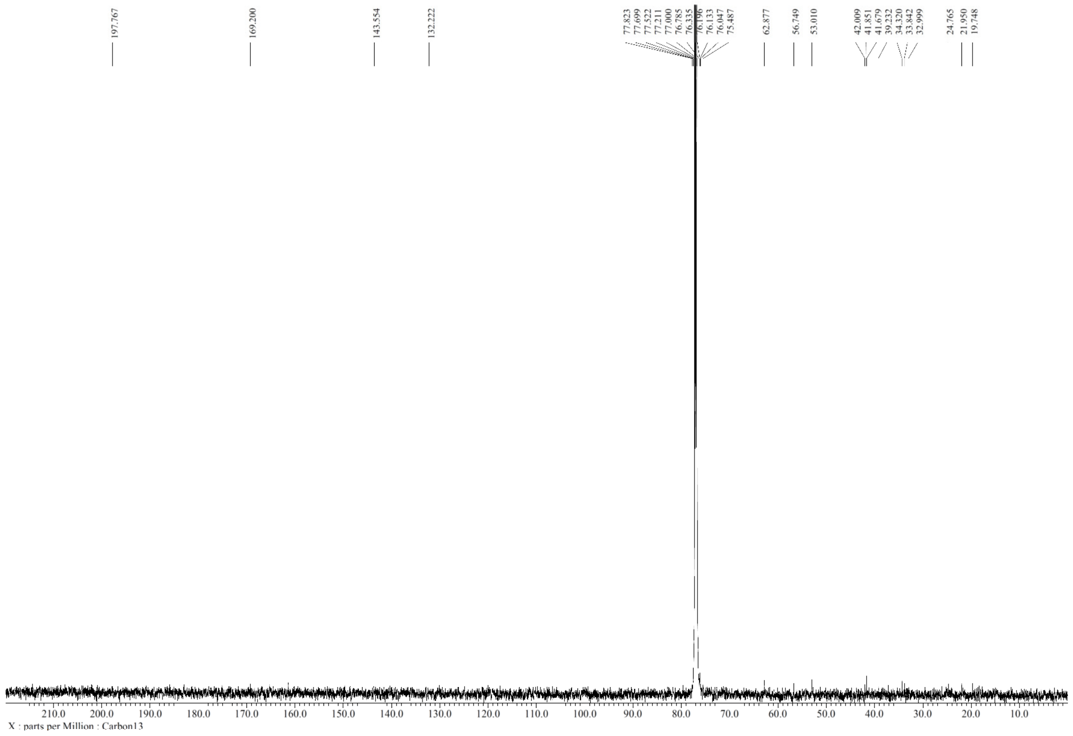

Figure S43. ^{13}C NMR spectrum (150 MHz) of compound **5** in $CDCl_3$.

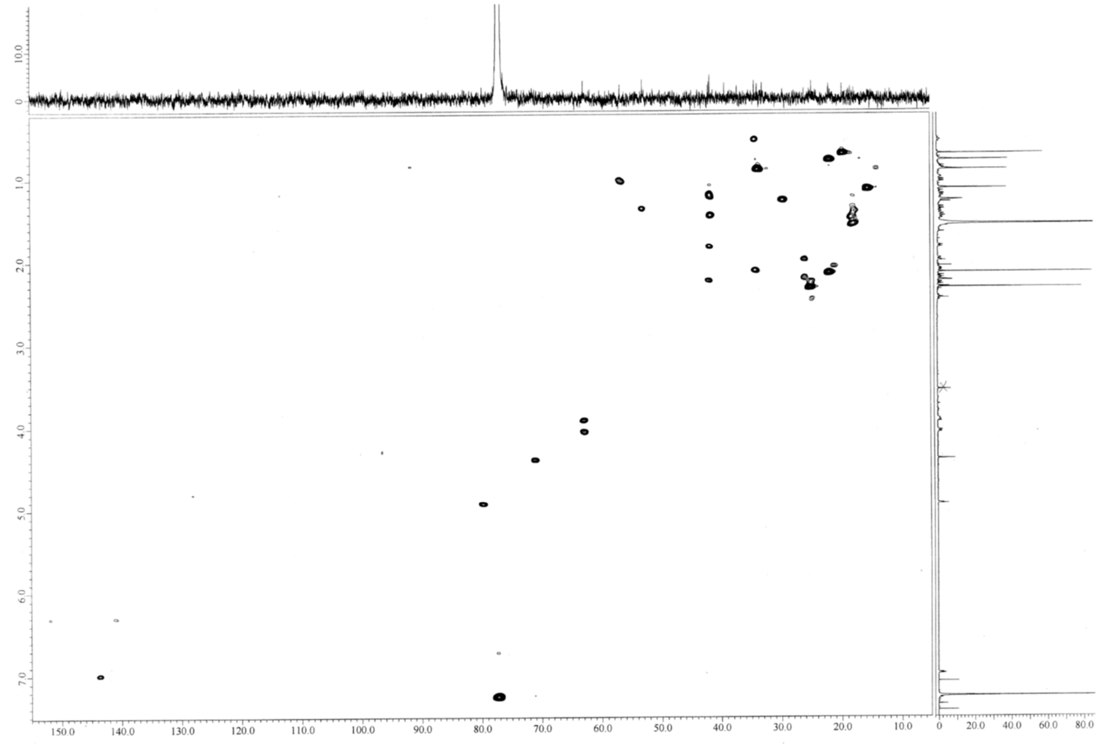

Figure S44. HSQC spectrum of compound **5** in $CDCl_3$.

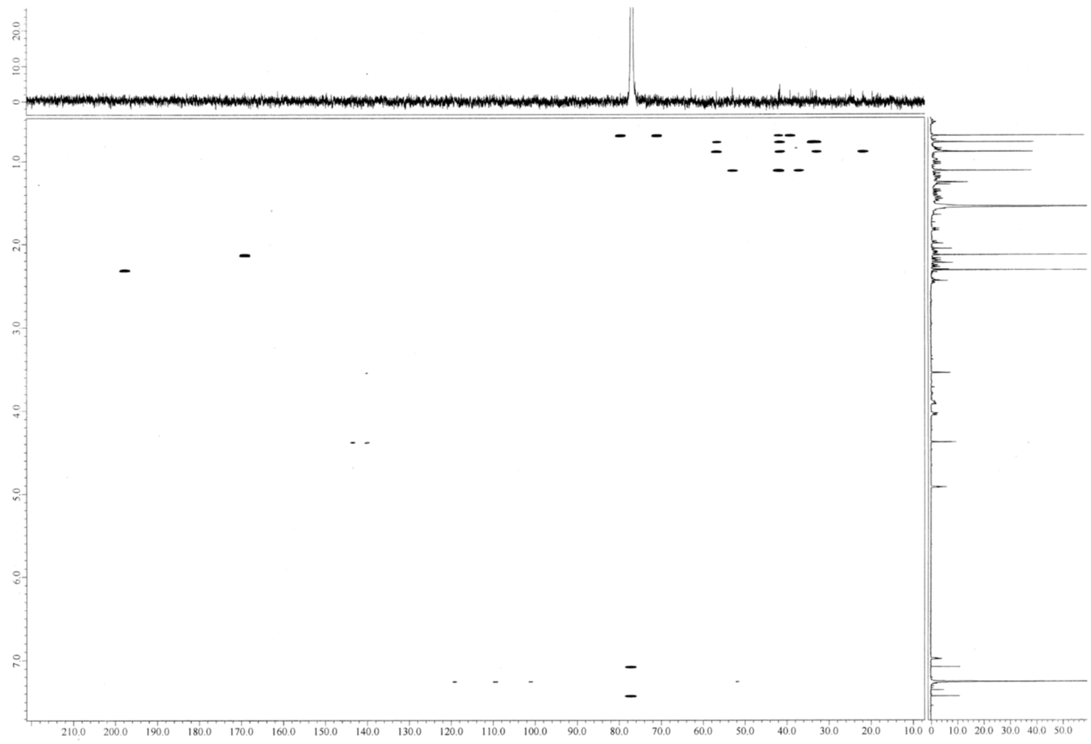

Figure S45. HMBC spectrum of compound **5** in $CDCl_3$.

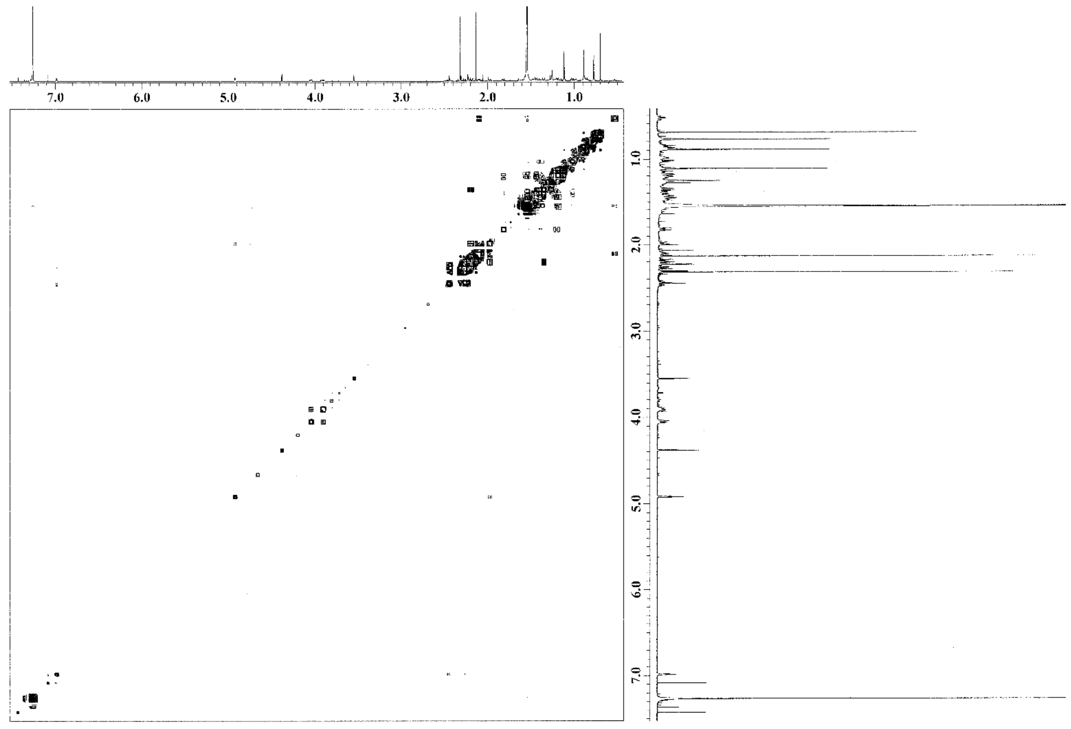

Figure S46. ^1H-^1H COSY spectrum of compound **5** in $CDCl_3$.

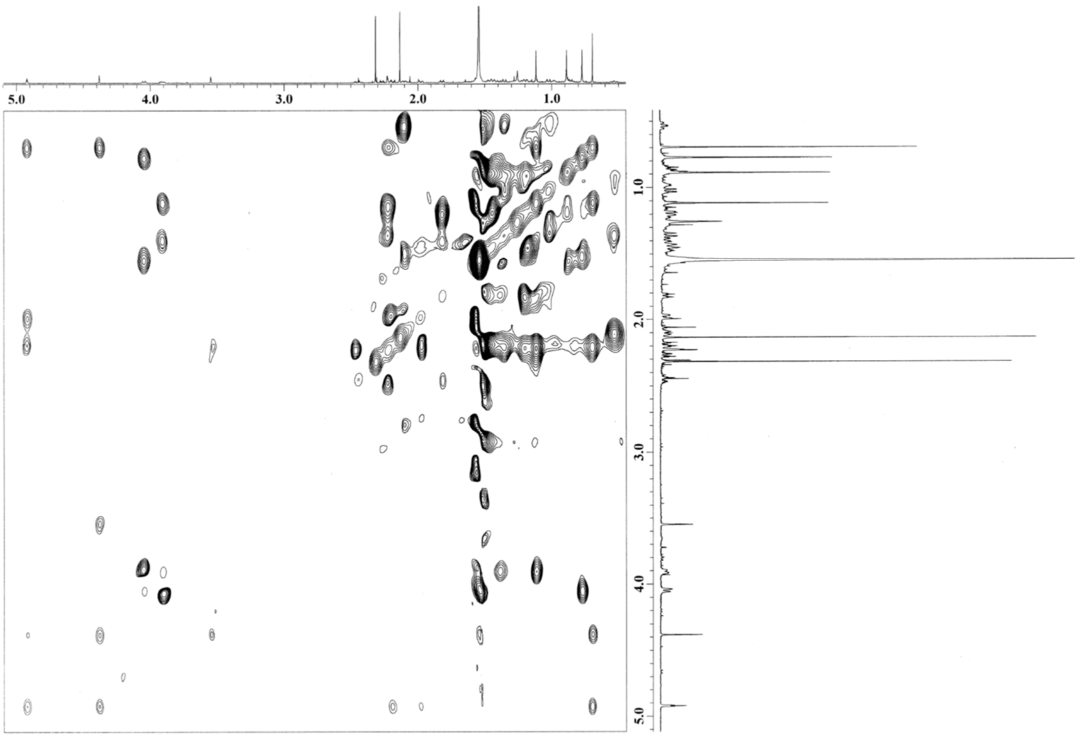

Figure S47. NOESY spectrum of compound **5** in $CDCl_3$

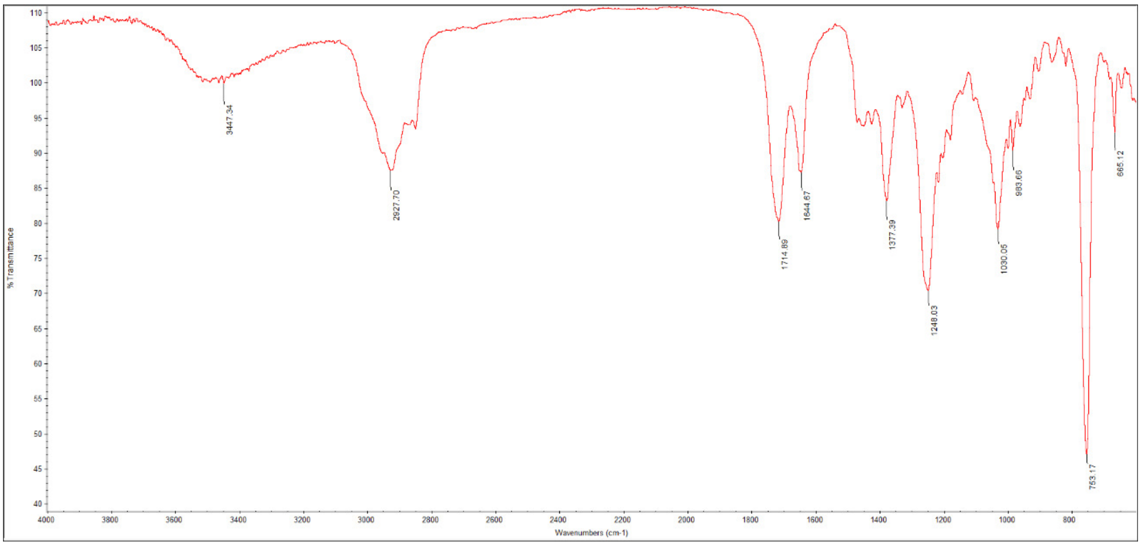

Figure S48. IR spectrum of compound **6.**

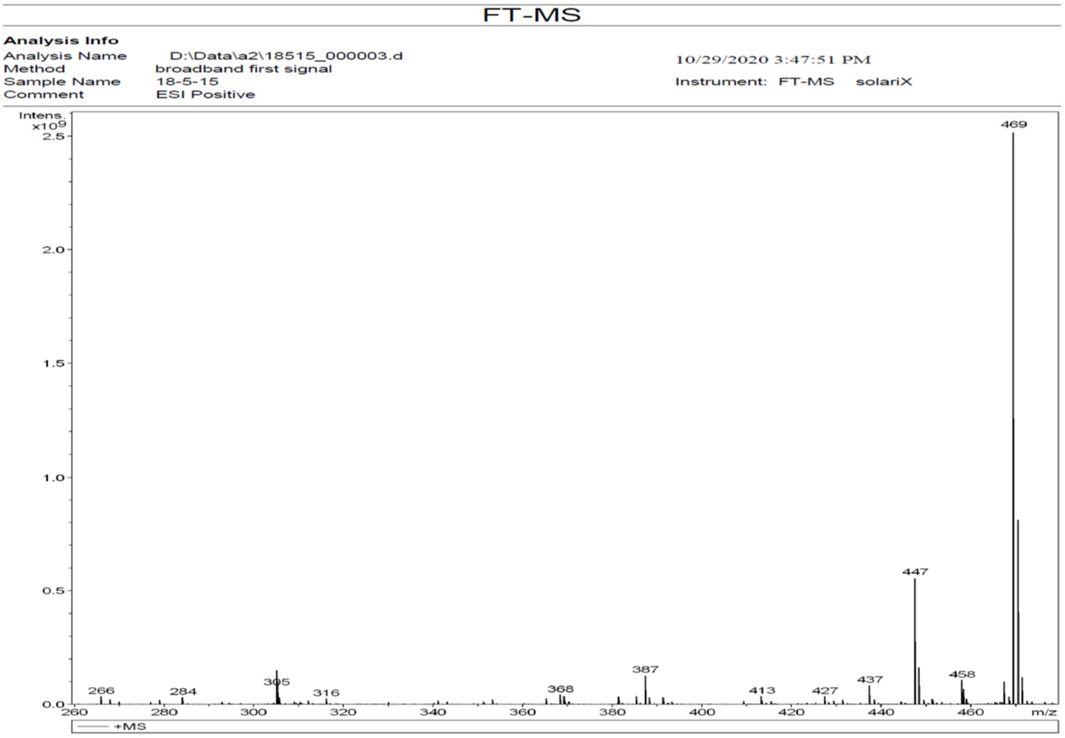

Figure S49. ESIMS spectrum of compound **6**.

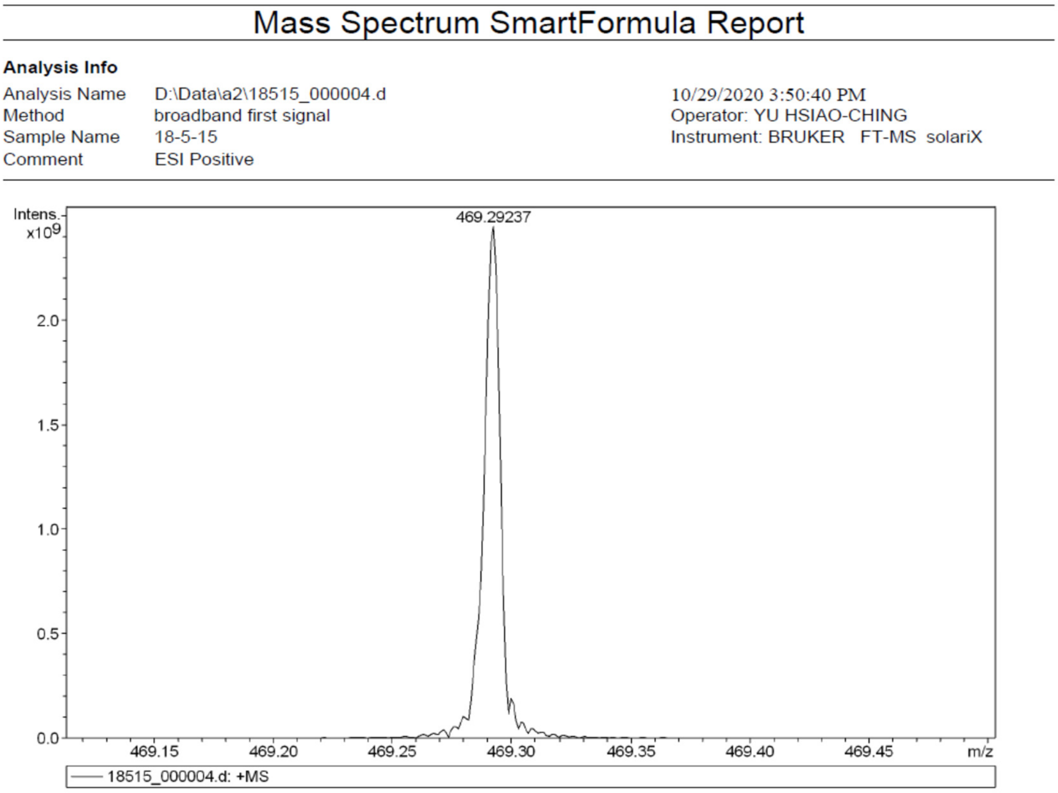

Meas. m/z	#	Formula	Score	m/z	err [mDa]	err [ppm]	mSigma	rdb	e⁻ Conf	N-Rule
469.29237	1	C 27 H 42 Na O 5	100.00	469.29245	0.07	0.15	10.4	6.5	even	ok

Figure S50. HRESIMS spectrum of compound **6**.

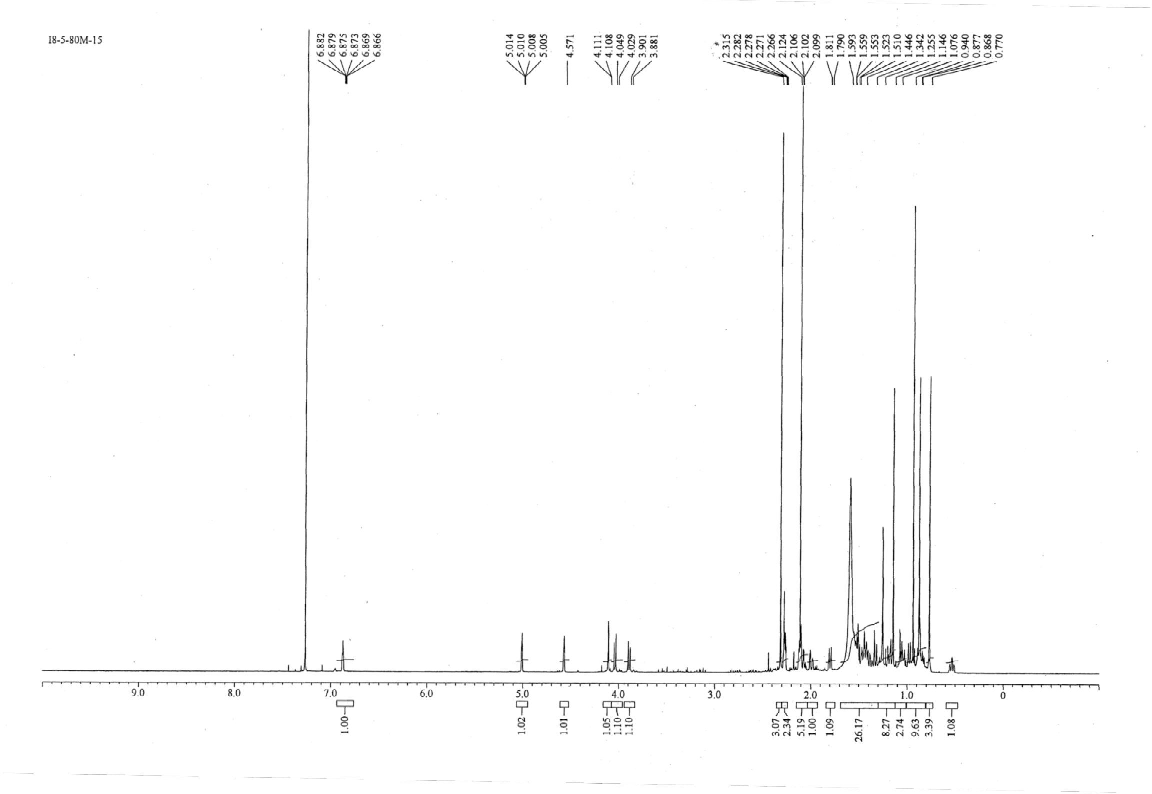

Figure S51. ^{1}H NMR spectrum (600 MHz) of compound **6** in $CDCl_3$.

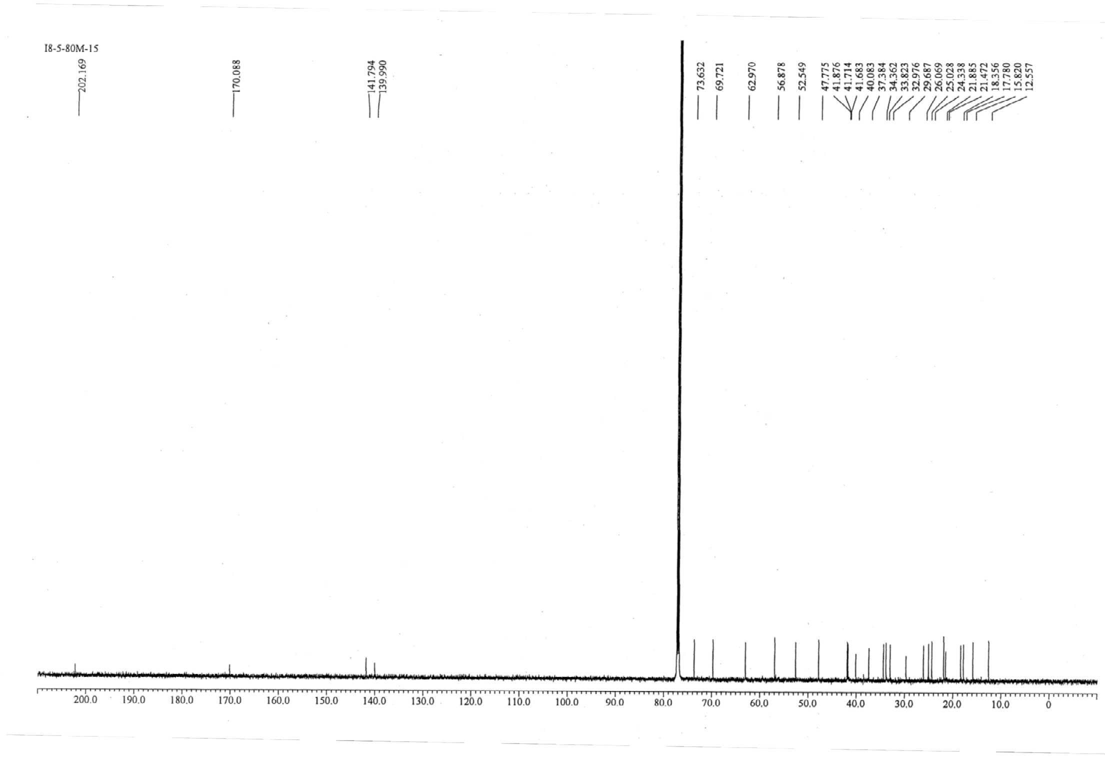

Figure S52. ^{13}C NMR spectrum (150 MHz) of compound **6** in $CDCl_3$.

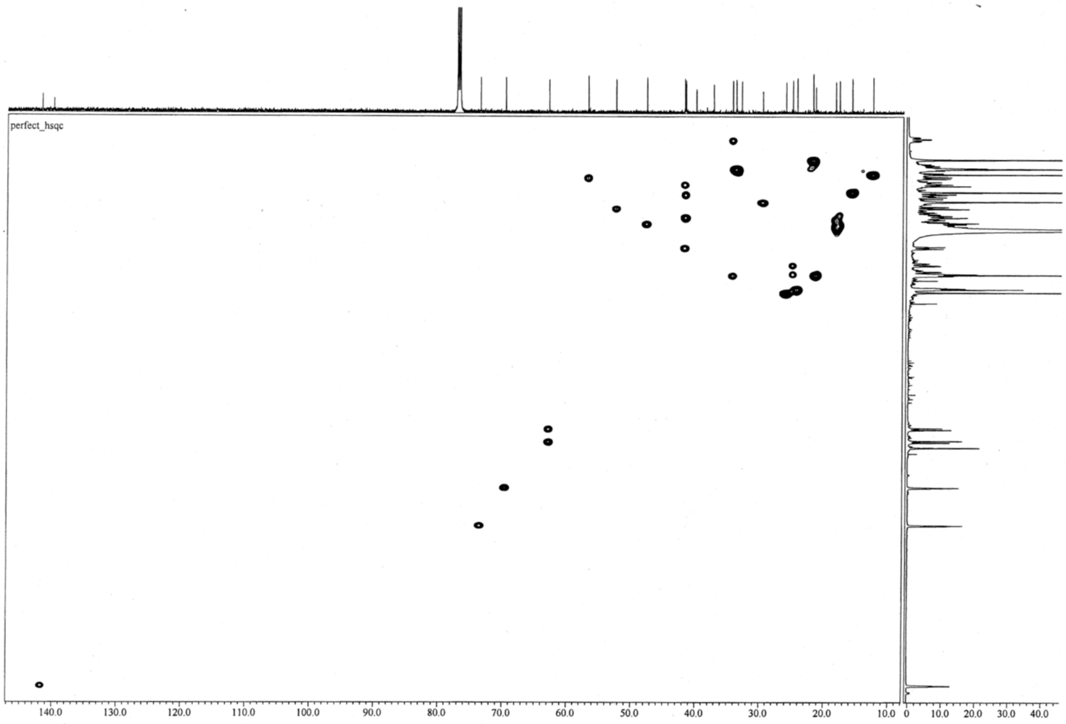

Figure S53. HSQC spectrum of compound **6** in $CDCl_3$.

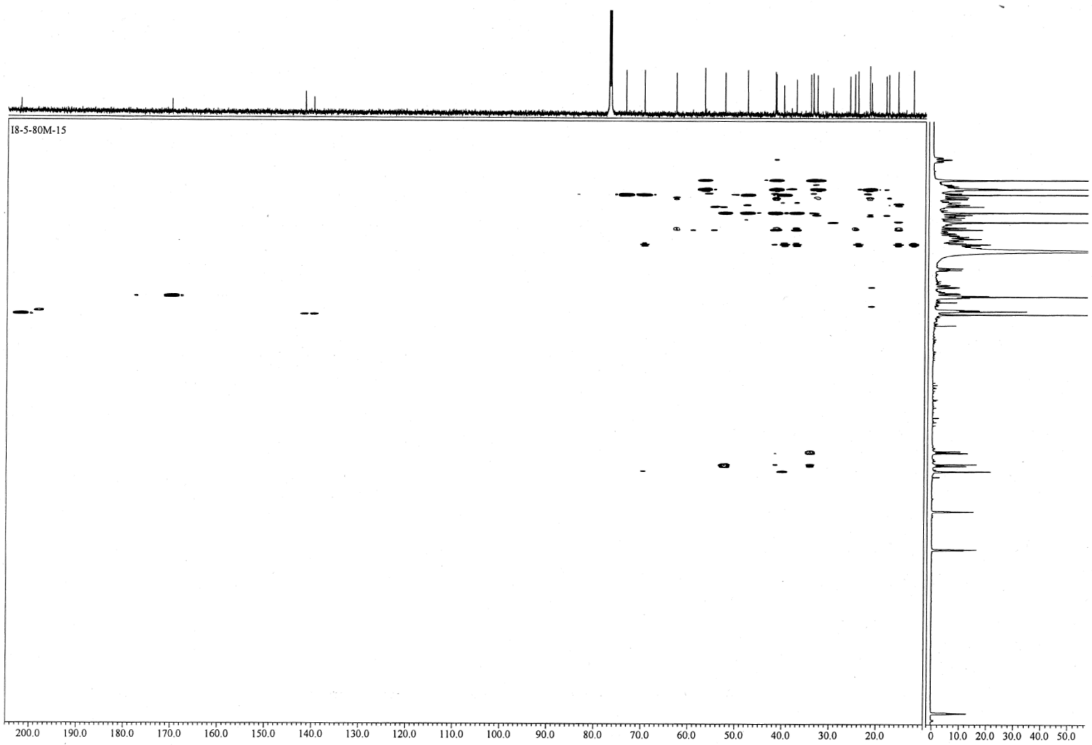

Figure S54. HMBC spectrum of compound **6** in $CDCl_3$.

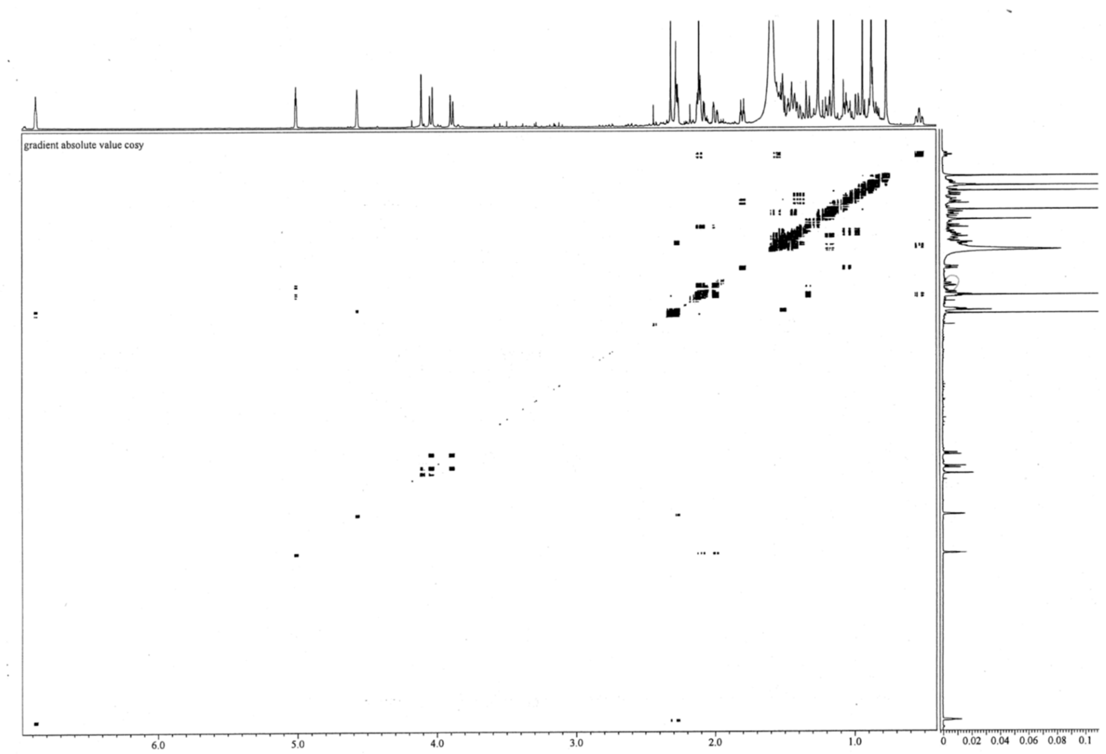

Figure S55. ^{1}H-^{1}H COSY spectrum of compound **6** in $CDCl_3$.

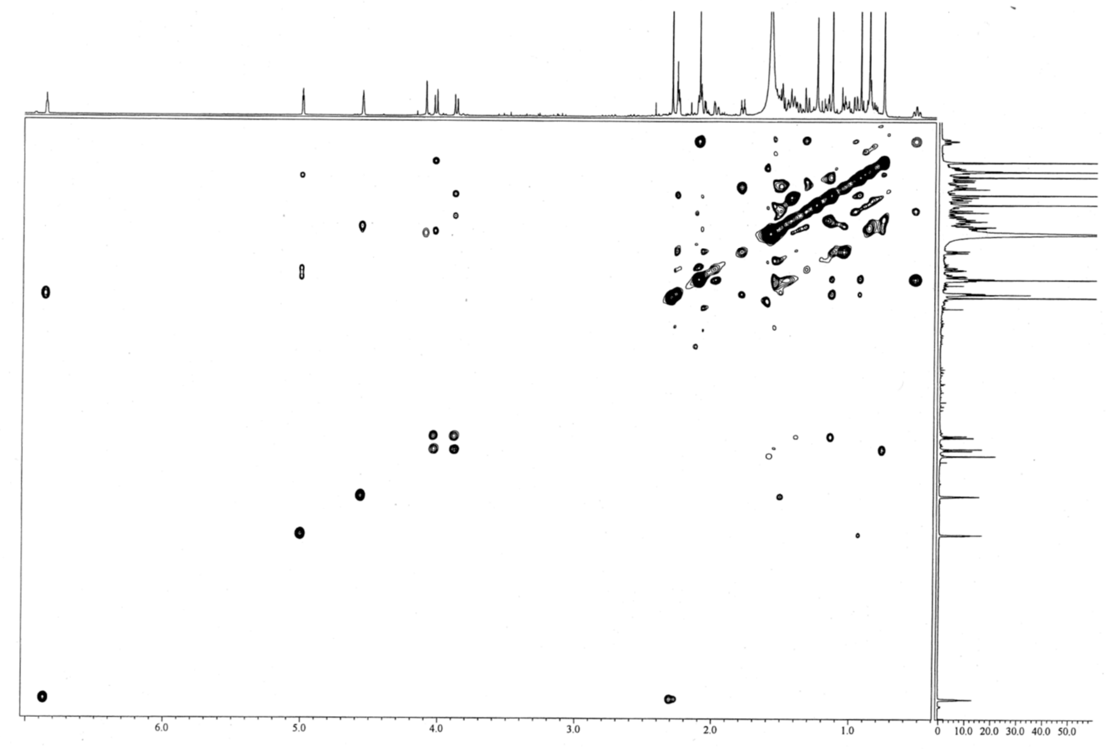

Figure S56. NOESY spectrum of compound **6** in $CDCl_3$

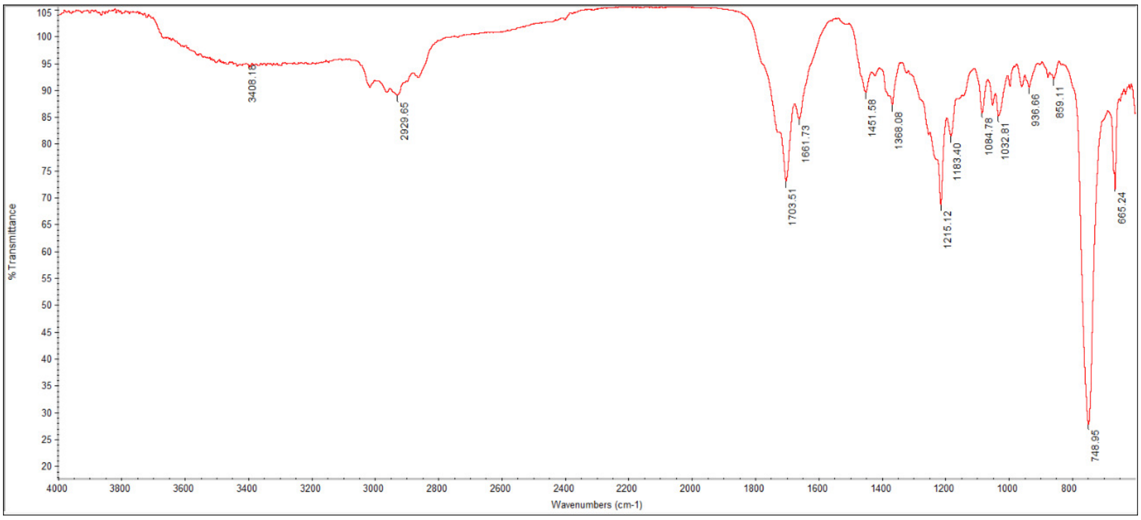

Figure S57. IR spectrum of compound **7.**

FT-MS

Analysis Info

Analysis Name	D:\Data\a2\1844_000003.d	10/29/2020 4:17:55 PM
Method	broadband first signal	
Sample Name	18-4-4	Instrument: FT-MS solariX
Comment	ESI Positive	

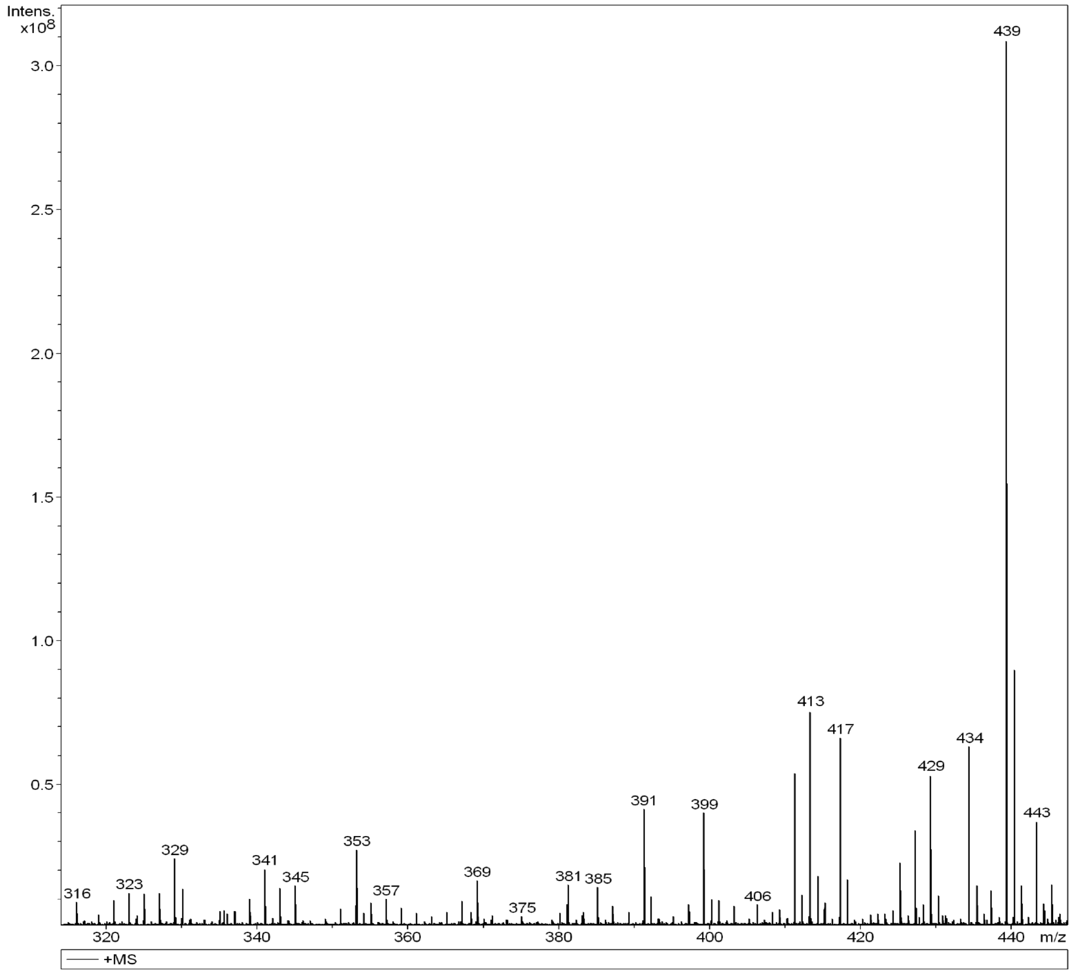

Figure S58. ESIMS spectrum of compound 7.

Mass Spectrum SmartFormula Report

Analysis Info

Analysis Name	D:\Data\a2\1844_000002.d	10/29/2020 4:16:52 PM
Method	broadband first signal	Operator: YU HSIAO-CHING
Sample Name	18-4-4	Instrument: BRUKER FT-MS solariX
Comment	ESI Positive	

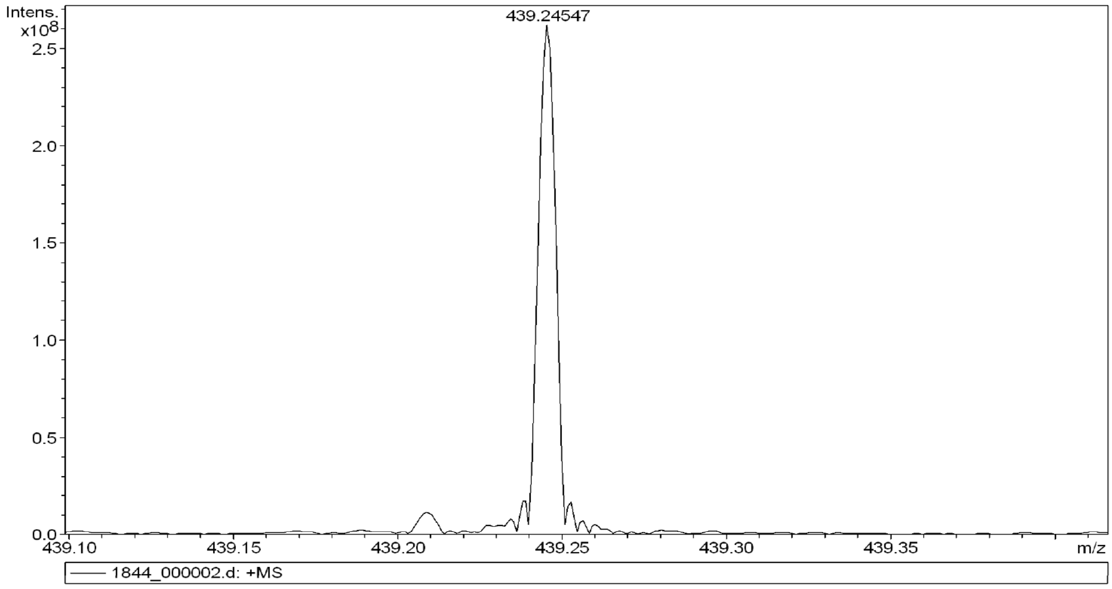

Meas. m/z	#	Formula	Score	m/z	err [mDa]	err [ppm]	mSigma	rdb	e⁻ Conf	N-Rule
439.24547	1	C 25 H 36 Na O 5	100.00	439.24550	0.03	0.07	4.9	7.5	even	ok

Figure S59. HRESIMS spectrum of compound **7**.

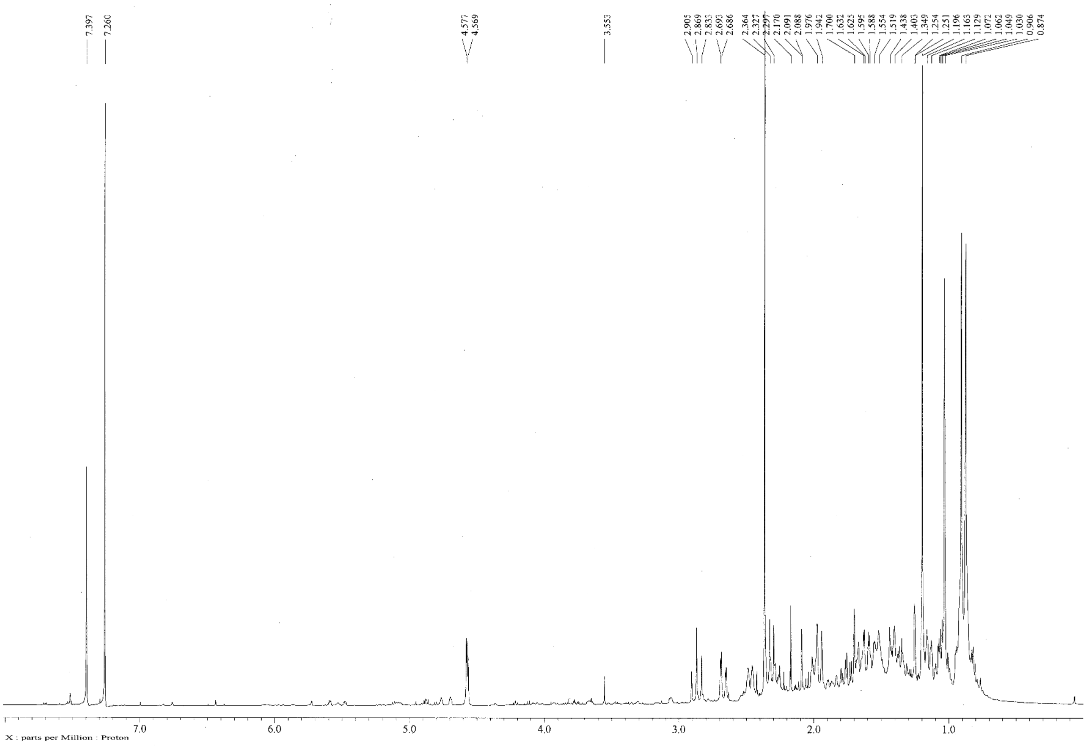

Figure S60. 1H NMR spectrum (400 MHz) of compound **7** in CDCl3.

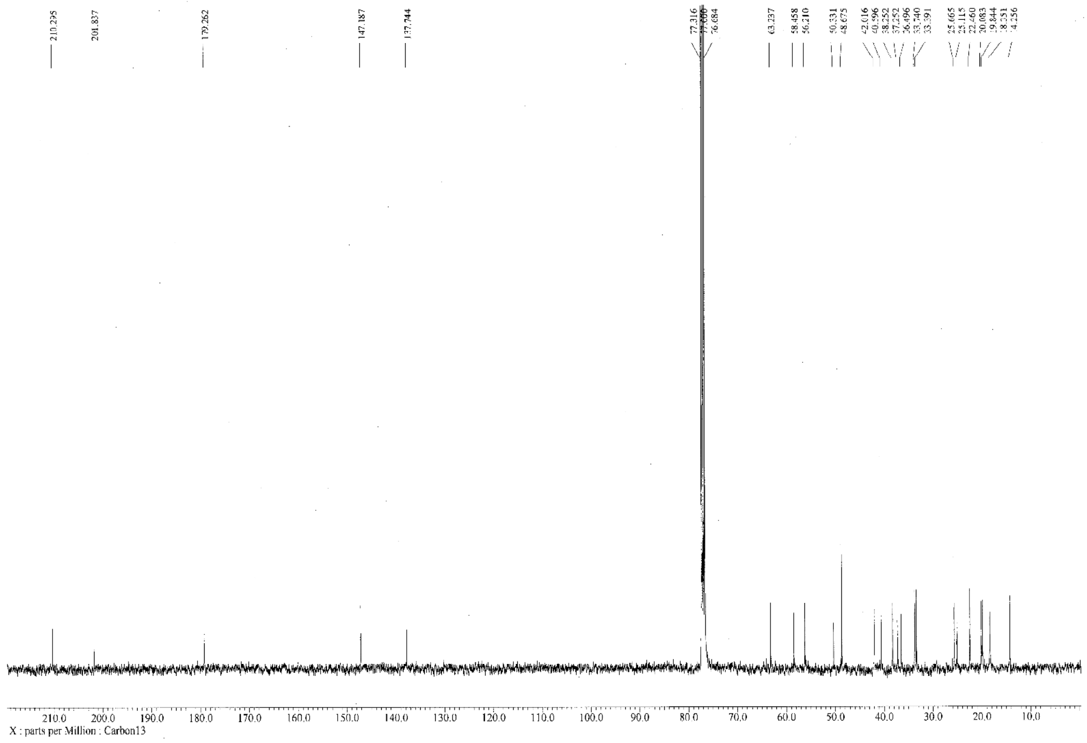

Figure S61. ^{13}C NMR spectrum (100 MHz) of compound **7** in $CDCl_3$.

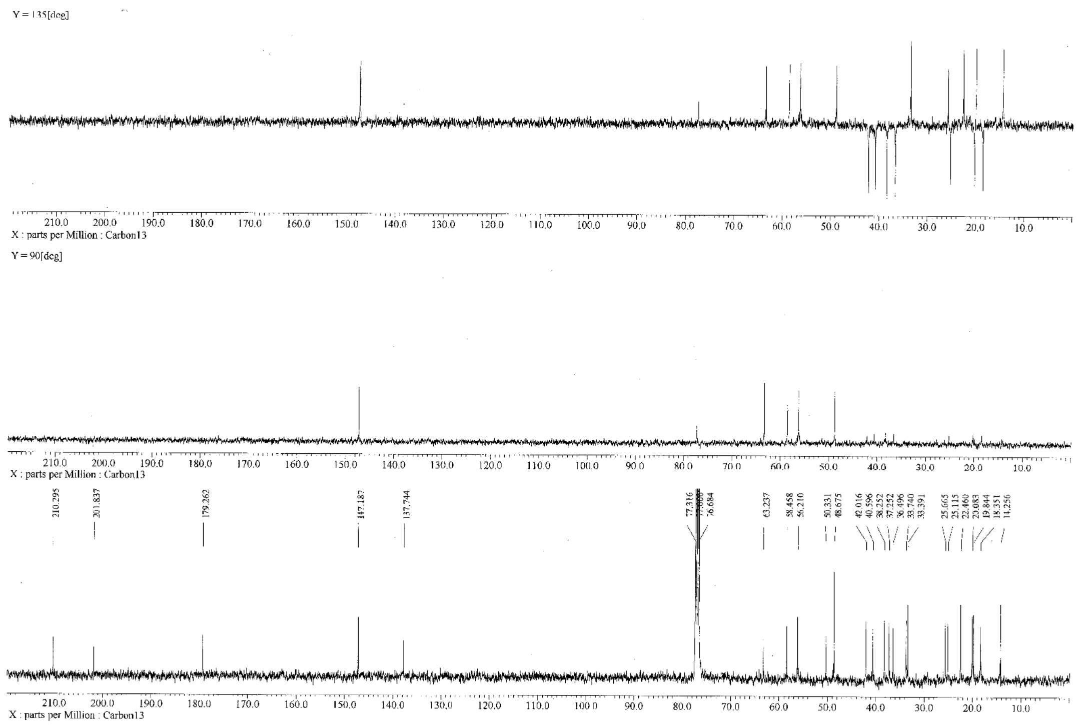

Figure S62. DEPT spectrum of compound **7** in $CDCl_3$.

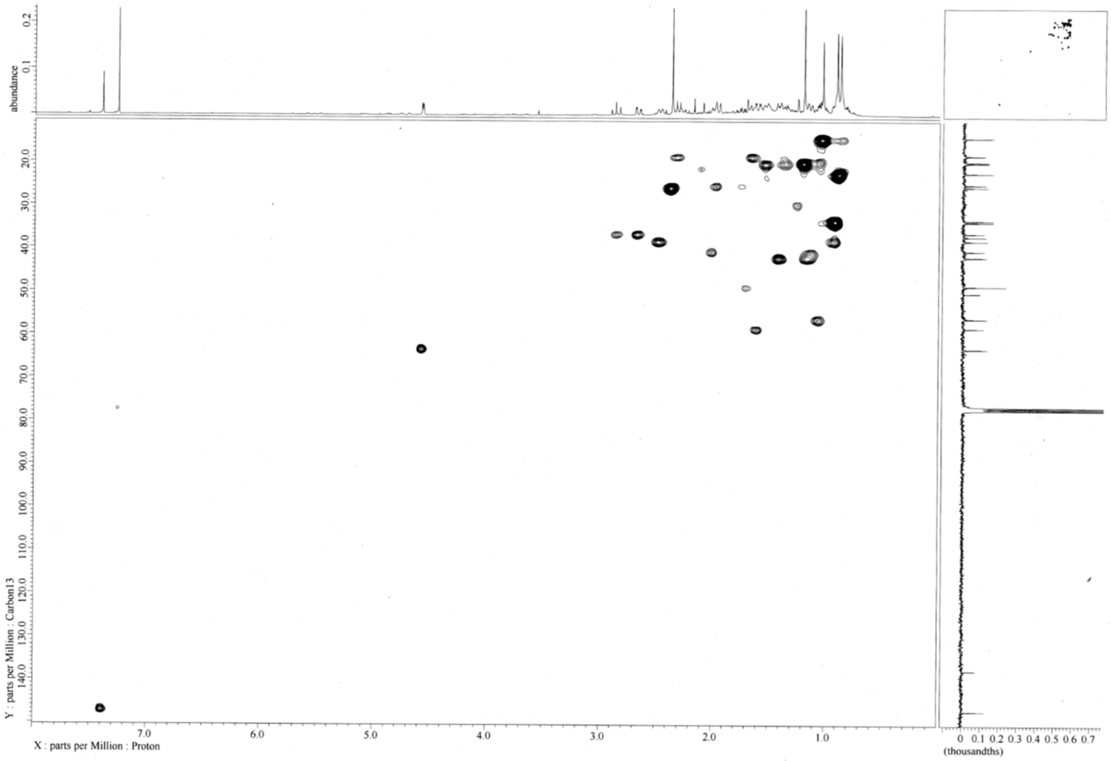

Figure S63. HSQC spectrum of compound **7** in $CDCl_3$.

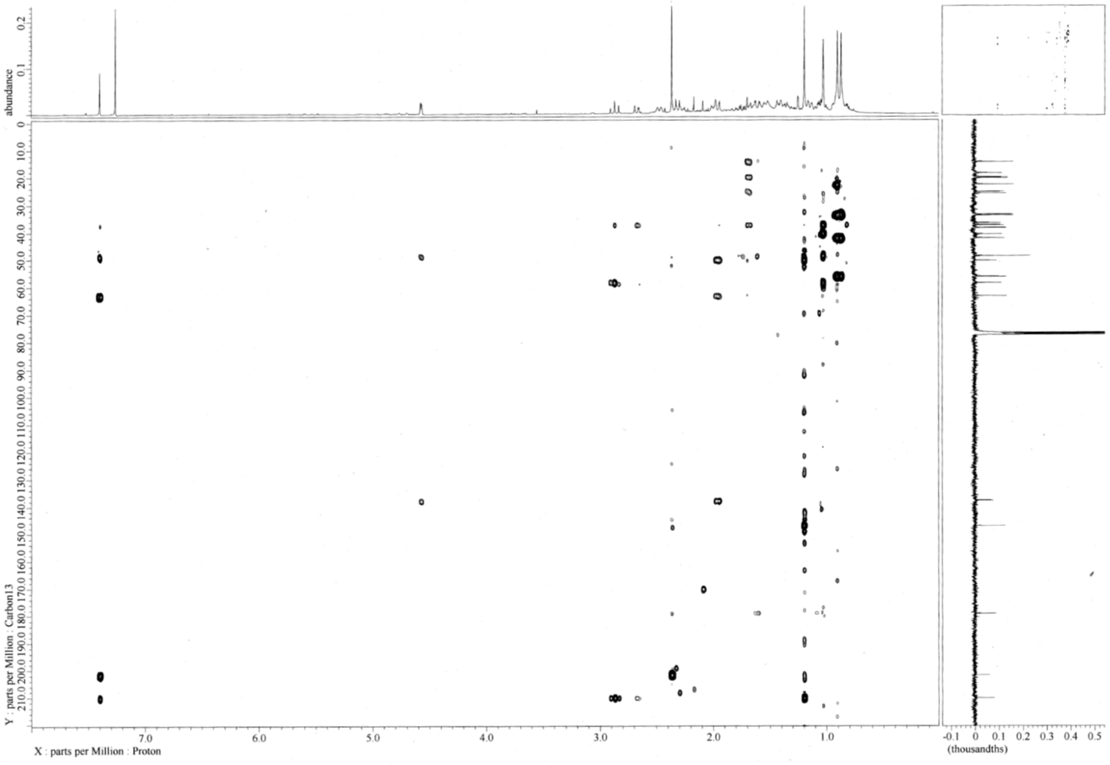

Figure S64. HMBC spectrum of compound **7** in $CDCl_3$.

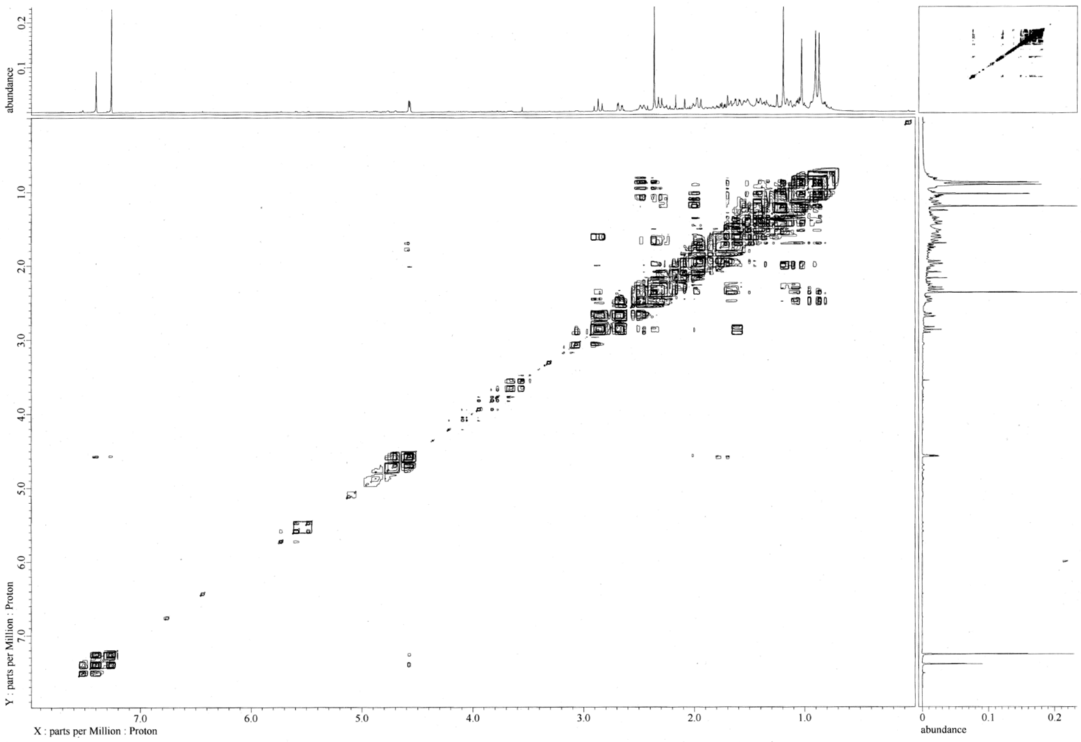

Figure S65. ^1H-^1H COSY spectrum of compound **7** in $CDCl_3$.

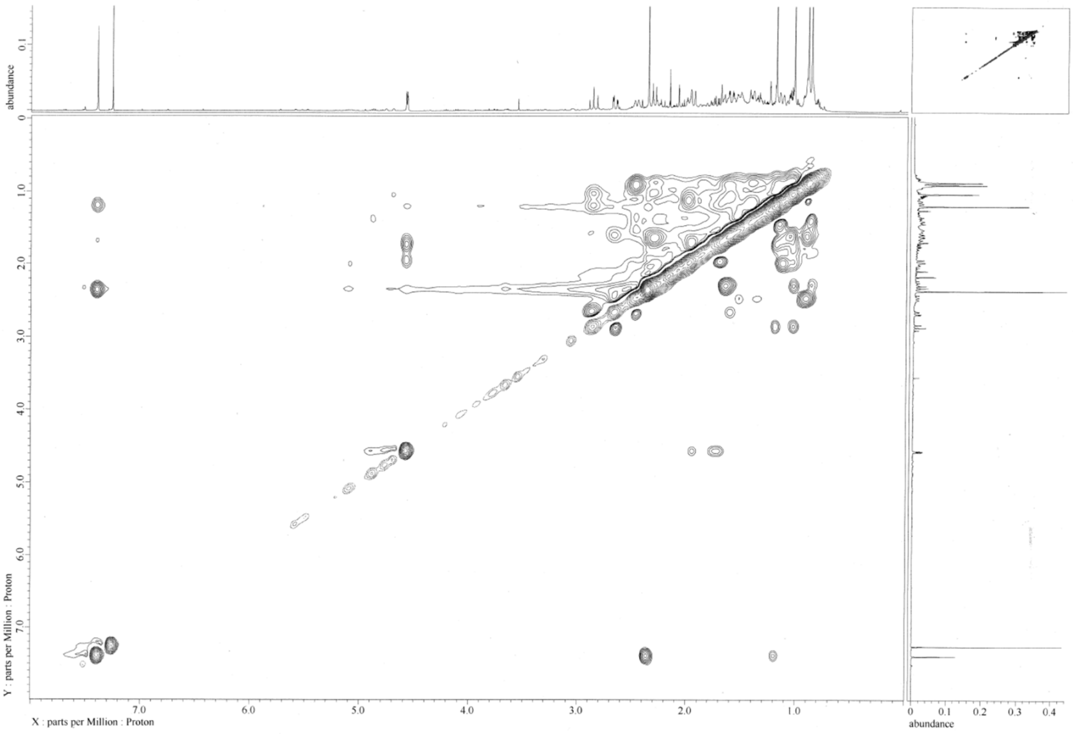

Figure S66. NOESY spectrum of compound **7** in $CDCl_3$
